# One-step extraction and determination of 513 psychoactive substances, drugs, and their metabolites from hair by LC–MS/MS

**DOI:** 10.1007/s00204-022-03343-w

**Published:** 2022-08-25

**Authors:** Jadwiga Musiał, Jolanta Powierska-Czarny, Jakub Czarny, Michał Raczkowski, Natalia Galant, Bogusław Buszewski, Renata Gadzała-Kopciuch

**Affiliations:** 1 Institute of Forensic Genetics, Al. Mickiewicza 3/4, 85-071 Bydgoszcz, Poland; 2 Department of Environmental Chemistry and Bioanalytics, Faculty of Chemistry, Nicolaus Copernicus University in Toruń, 7 Gagarin St., 87-100 Toruń, Poland

**Keywords:** New psychoactive substances (NPS), Drugs, Metabolites, Hair analysis, Liquid chromatography-tandem mass spectrometry (LC–MS/MS)

## Abstract

The emergence of new psychoactive substances on the market is a significant problem on a global scale. This type of substance in society is associated with many negative consequences, such as traffic accidents, accidents at work, rape, homicide, poisoning, or overdose deaths. The analysis of these substances in biological samples is very important for further legal action and saving lives. Therefore, laboratories face a tremendous challenge in tackling the evolving drug market. The paper describes the optimization of the analytical LC–MS/MS method to identify and determine 513 psychoactive substances in hair samples. A method of chromatographic separation was developed, and the working parameters of the mass spectrometer were selected for each analyte. The method has been validated, and the results are as follows: the limit of quantification of the developed method ranges from 0.025 to 1.25 ng/mg hair. The mean recovery of the tested analytes ranges from 80 to 120%. The achieved coefficient of variation in within-run precision ranged from 1.05 to 19.99%. The results achieved for BIAS are in the range of ± 20%.

**Supplementary Information:**

The online version contains supplementary material available at 10.1007/s00204-022-03343-w.

## Introduction

Every year we observe an increase in interest in psychoactive substances, and new psychoactive substances (NPS) appear more and more often. They are considered a legal alternative to alcohol or popular illegal drugs such as amphetamines, cocaine, and MDMA. In the World Drug Report 2017 of the United Nations Office on Drugs and Crime (UNODC), 729 NPS were reported in 2009–2016 (World Drug Report 2017). Most of them belong to the group of synthetic cathinones or synthetic cannabinoids (World Drug Report 2017). These substances are chemically similar to the natural compounds corresponding to these groups; this makes them difficult to identify and they often remain undetected during routine analyses. The spread of these substances on the market is associated with health and life-threatening effects. Laboratories are constantly developing new methods of analyzing such substances in samples of saliva, urine, blood, or plasma of people suspected of taking drugs. However, such samples are not always collected in good time after the event. They are often given to another person for the purpose of committing a crime. These are difficult to detect because, by the time the person who has been given the illegal substance realizes it and reports for testing, it can no longer be detected even though it has been taken.

An alternative to these standard matrices are hair samples. Hair grows on average 0.35 mm/day or 1–1.5 cm/month, depending on anatomical location, race, sex, and age (Rivier 2000), so the portion of a hair located 3 cm from the scalp was formed by cells in a hair follicle about three months earlier. Thus it is likely that the substance introduced into the hair will appear 3 cm from the scalp three months after the substance was taken (Rivier 2000). There are two known ways to incorporate a substance/drug into the hair. These are (1) adsorption from the external environment to and into the developed hair shaft, and (2) transport to the growing hair shaft through the blood supply to the hair follicle (Rivier 2000). Both drugs and psychoactive substances accumulate in the hair, making it possible to detect them long after the event. Depending on the length of the hair, a retrospective analysis up to several months back from the date of sample collection can be carried out. It is also possible to determine whether the substance has been taken once or repeatedly/regularly, which is essential particularly if the use of psychoactive substances was related to crime or criminal behavior. The advantages of this matrix also include non-invasive sampling. Moreover, both non-metabolized substances and metabolites produced in the organism are accumulated (Moffat et al. 2011). Testing hair is often the only way to determine metabolites derived from synthetic cannabinoids. Studies (Sobolevsky et al. 2010; Grigoryev et al. 2011, 2013; Adamowicz et al. 2013; Kavanagh et al. 2013) observed that some substances in this group are metabolized at a high rate, which results in the absence of a parent analyte in the urine.

Despite the enormous potential of this matrix, there are few studies on NPS analysis in hair samples (Gottardo et al. 2014; Salomone et al. 2014; Hutter et al. 2012; Rust et al. 2012; Martin et al. 2012, 2015; Wyman et al. 2013; Strano-Rossi et al. 2014; Lee et al. 2011; Marsh et al. 2014). Two of these studies attempted to determine synthetic cannabinoids by incubating a hair sample in concentrated sodium hydroxide to remove keratin (Gottardo et al. 2014; Salomone et al. 2014). For the extraction, the authors used hexane/ethyl acetate 90:10 (v/v), and the obtained organic phase was evaporated to dryness under a stream of nitrogen. The sample was then reconstituted in methanol and analyzed by liquid chromatography coupled to mass spectrometry. The downside of this method is the need for additional extraction, which extends the analysis time and increases the risk of analyte loss. Hutter et al. (2012) also determined synthetic cannabinoids in the hair by liquid chromatography coupled with mass spectrometry (LC–MS/MS). However, they used ethanol for the extraction. The sample was then evaporated to dryness and reconstituted in mobile phase 50:50 (v/v). A two-step extraction method was presented by Rust et al. (2012). In the first stage, they used methanol with ultrasonication, and in the second, methanol was acidified with hydrochloric acid. Then, as in the previous work, the sample was evaporated to dryness and analyzed by LC–MS/MS. Another study (Lee et al. 2011) analyzed benzodiazepines with solid-phase extraction (SPE); gas chromatography with mass spectrometry (GC–MS) was used for the determination. Gas chromatography was also applied in the study on the analysis of mephedrone in hair samples (Martin et al. 2012). SPE was also used to extract psilocin, bufetenine and LSD (Martin et al. 2015).

This variety of extraction methods emphasizes how complex matrix the hair is. The analysis of hair samples is an enormous challenge and, at the same time, great hope for the work of toxicologists. This study aimed to develop an analytical method that allows quick yet sensitive and specific hair sample analysis for drugs and psychoactive substances, including NPS and their metabolites. The developed method is characterized by a rapid, single-step extraction of a wide range (513) of analytes, which are determined by LC–MS/MS operating in the MRM mode. This method was developed to enable the fight against drug crime and addictions and aid research on detecting this type of substance in specific social groups such as drivers or students.

## Reagents and materials

The certified analytical standards of analyzed substances were purchased from Cayman Chemical, CHIRON, Lipomed Services to Health, and LGC Standards. To prepare samples and standard stock solutions, acetonitrile (ACN) for LC–MS, methanol (MeOH) for LC–MS, and formic acid for LC–MS, and dichloromethane were purchased from S. WITKO CHS, and ammonium formate for LC–MS was purchased from Sigma Aldrich. Working solutions were prepared by dilution of stock solution.

## Calibration curve in matrix procedure

A section of hair (2 × 1 cm) without test analytes was collected into two 5 mL syringes. The hair was washed three times with 3 mL of dichloromethane. A syringe protected with a hair-impermeable filter was shaken, and the hair was then allowed to dry. The dried hair was pulverized (5 min; 15,000 rpm). 20 mg of the powdered hair was weighed into an Eppendorf tube; then 0.5 mL of methanol was added to it. The sample prepared in this way was shaken for 1 h at 21 °C and 12,000 rpm. During the next step, the sample was placed in the freezer for 10 min, followed by centrifugation at 5 min and 2000 rpm. Ten μL of the internal standard (atrazine) at a concentration of 500 ng/mL, 50 μL of the mix of standards at the appropriate concentration (at six different concentration levels in the range 0.025–5 ng/mg), 50 μL of hair extract, and 390 μL of mobile phase A: B (90:10, v/v) were transferred to the filter basket and mixed by hand. The mixed sample was placed back in the freezer for 10 min and centrifuged for 3 min and 10,000 rpm. 200 µL of thus obtained extract was withdrawn into a reduced volume vial.

## Modification of the extraction method from hair samples

Many different parameters have been tested to develop a method of isolating psychoactive substances, drugs and their metabolites from hair samples. We were starting from the washing stage and ending with the filtration stage. The solvents used to wash the hair samples were verified, dichloromethane, dichloromethane and ethyl alcohol were checked. More favorable results are obtained when using only dichloromethane. In the next stage, the volume of this solvent used for washing and the number of repetitions was checked. In this case, washing was considered best three times with 3 ml of dichloromethane. The next stage that underwent modifications was drying the hair samples. Two options for drying the samples were tested. The first was sewing on tissue paper, and the second was drying with the use of an airtight dryer. In this case, lower losses of analytes were obtained for spontaneous drying on blotting paper. During the sample powdering stage, the amount of sample subjected to powdering and the number and size of the beads used for powdering were verified. The best results were obtained for a dose of about 20 mg and the use of one powdering ball with a diameter of 25 mm. In the next step, two extraction solvents were checked. It was methanol and acetonitrile. The use of acetonitrile did not contribute to the obtaining of better analysis results, and therefore, due to the harmfulness of the waste and the price of solvents, methanol was selected for further analysis. The tests also checked for re-freezing before the last centrifugation. However, the results showed that this freezing had no effect on the percent recovery of the analyte from the sample, so it was removed from the procedure.

## Hair sample preparation procedure

A section of hair removed from a tested person (1 cm) was placed in a syringe with a volume of 5 mL. The hair was washed three times with 3 mL of dichloromethane. A syringe protected with a hair-impermeable filter was shaken, and the hair was then allowed to dry. The dried hair was pulverized (5 min; 15,000 rpm). 20 mg of the powdered hair was weighed into an Eppendorf tube. Then 20 μL of the internal standard (atrazine) at a concentration of 2500 ng/mL and 0.5 mL of methanol were added to the Eppendorf. The sample prepared in this way was shaken for 1 h at 21 °C and 12,000 rpm. The sample was placed in the freezer for 10 min, followed by centrifugation at 5 min and 2000 rpm in the next step. Fifty µL of hair extract and 450 µL of mobile phase A: B (90:10, v/v) were transferred to a filter basket and mixed by hand. The mixed sample was placed back in the freezer for 10 min and centrifuged for 3 min and 10,000 rpm. 200 µL of thus obtained extract was withdrawn into a reduced volume vial.

## Chromatographic separation

Chromatographic separation was achieved by gradient elution on the liquid chromatography system consisted of an ExionLC AC Pump 2x, ExionLC Degaser, Exion AC Autosampler, and ExionLC Column Oven from AB SCIEX. Separation was carried out on a Kinetex C18 column (Phenomenex, 3.0 × 100 mm; 2.6 μm). A 20 μL sample was injected into the system at a flow rate of 0.5 mL/min. The gradient LC system was operated using ammonium formate 2 mM with 0.1% formic acid (mobile phase A) and 2 mM ammonium formate in MeOH with 0.1 formic acid (mobile phase B).

Optimum gradient elution for separation on chromatographic column was performed: 0–1 min (95% A, 5% B), 1–15 min (linear gradient to 5% A), 15–21 min (5% A, 95% B), 21–27 min (linear gradient to 95% A), 27–30 min (95% A, 5% B). Acetonitrile (ACN) was also checked in mobile phase B. The obtained results were not more favorable. The costs of the analyses were higher, and the waste generated during the analyses was more harmful. The influence of ammonium formate concentration on the results of the analyses was also checked. Values from 2 to 5 mM were checked. Increasing the ammonium formate concentration did not improve the separation of the analytes on the chromatography column. The optimized separation conditions of the analytes on the Kinetex C18 column (Phenomenex, 3.0 × 100 mm; 2.6 μm) were also checked on the other two columns Kinetex Biphenyl (Phenomenex, 3.0 × 100 mm; 2.6 μm) and Kinetex Phenyl-Hexyl (Phenomenex, 3.0 × 100 mm; 2.6 μm). The best results were obtained for the first column, and it was selected for further analysis. This gradient method allowed for the separation of all compounds except 3-MMC and 4-MMC in a 30 min run time. The retention times of all compounds were from 1.34 to 16.76 min and are presented in electronic supplementary material Table S1. The chromatogram obtained for the selected column and gradient is shown in electronic supplementary material Figure S1.

## Mass spectrometric detection

The analysis was performed on the mass spectrometer AB SCIEX 5500 QTRAP with electrospray ionization in the positive mode. Data acquisition, data handling and instrument control were performed by Analyst 1.6.3 and MultiQuant 3.0.3 software. Analytes were quantified in the double ion monitoring (MRM) mode. The spectrometric analysis parameters were optimized, and two MRM pairs were selected for each analyte according to the mass spectrometry standards. All results were based on the peak area ratio between the drug and the analytical standard. The MS conditions were set as followed: CUR: 30, CAD: medium, TEM: 400, GS1: 40, GS2: 70, dwell time ≥ 5 ms.

Analytical standards of all analyzed substances were subjected to individual optimization to select the best parameters of the mass spectrometer for each of them. Analyst 1.6.3 was used for this optimization. The conducted analyzes allowed for selecting parameters such as the ionization mode, Q1, Q3, declustering potential (DP), entrance potential (EP), collision energy (CE), collision cell exit potential (CXP). The positive ionization mode turned out to be better for the tested substances. The entrance potential (EP) for all analytes is 10. The MRM pairs and values DP, CE, CXP for each analyte have been collected in electronic supplementary material Table S1. In the developed method, MRM transitions for 517 analytes (two transitions for each analyte) were monitored only in specific detection windows that were defined on ± 0.5 min from the expected retention time.

## Validation parameters

This presented analytical method was validated according to the SWGTOX validation guidelines (Scientific Working Group for Forensic Toxicology 2013). Fortification of hair samples was performed by adding 20 μL of the standard mix with a concentration of 200 ng/mL (corresponding to a concentration of 0.2 ng/mg hair to the matrix together with 0.5 mL of methanol. A blank sample was prepared by adding to the basket 10 μL of atrazine at a 500 ng/mL concentration, 50 μL of the obtained hair extract containing no test analytes, and 440 μL of the mixture of mobile phases A: B (90:10, v/v). The following steps were performed according to the hair sample preparation procedure.

Linearity validation was performed by analyzing six separate calibration curves in the matrix with concentrations ranging from 0.025 to 1.250 ng/mg for cannabinoids and from 0.125 to 5 ng/mg for other analytes. A blank matrix and blank matrix containing only internal standards were analyzed with each batch but not included in the calibration curves. Linearity was described using a weighted linear regression plot of peak area ratio (PAR) vs. spiked analyte concentration. Calibration curves in the matrix were linear in the range of 0.025–1.25 ng/mg for cannabinoids and 0.125–5 ng/mg for other analytes. Correlation coefficients (*R*^2^) calculated for each analyte were ≥ 0.99. The calibration curve prepared in the matrix allowed eliminating the influence of the matrix effect in the actual samples.

To evaluate the precision and BIAS for our method, six replicates of calibration standards for three different concentrations from the calibration scale (0.025 ng/mg; 0.125 ng/mg, and 1.25 ng/mg for cannabinoids) and (0.125 ng/mg; 1.25 ng/mg and 5 ng/mg for other analytes) were used. For these parameters, an accuracy limit of ± 20% was used. The six repetitions were also used to determine the mean recovery. Accuracy was expressed as the average percentage recovery, and resulting values ranged from 80 to 120%.

According to the recommendations in the SWGTOX guideline (Scientific Working Group for Forensic Toxicology 2013), precision was evaluated by CV.

The determined CV for the tested analytes ranged from 1.05 to 19.99%, and BIAS ranged from − 20.00% to 20.00%. For precision and BIAS accuracy limit of ± 20% was used. The results of precision, BIAS, and recovery values for validated compounds are presented in electronic supplementary material Table S2.

The LOQ was determined to be the lowest calibration standard exhibited as S/N ratio ≥ 10. Proficiency tests were used to check the reproducibility of the analytical method. The estimated values of LOQ were from 0.025 to 1.25 ng/mg for 465 compounds, and the value was 0.5 ng/mL for other compounds. The LOQ values for each analyte are presented in electronic supplementary material Table S2.

**Fig. 1:**
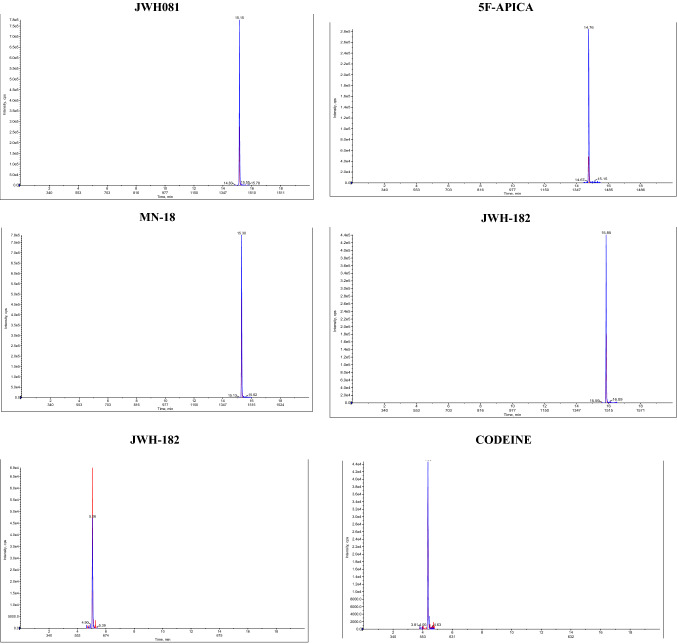

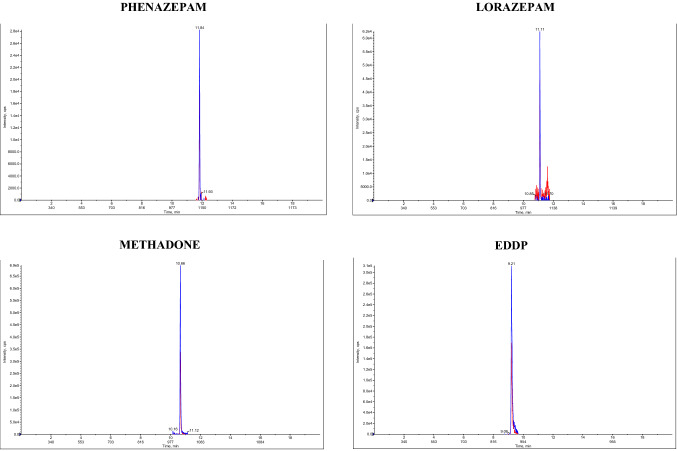
Peaks obtained for several analytes included in the standard mixture

Selectivity and specificity were assessed by spiking fortified samples with each analyte in a small mix to test for any interference. Hair without analytes was used to identify matrix interferences. It was possible to separate the isomers within the run time by gradient liquid chromatography. To verify the matrix effect, a standard curve in the matrix was used. The obtained points of the standard curve take into account the influence of the matrix on the analysis of actual samples. The mass spectrometer used enables the identification of the molecules of interest with high selectivity and specificity. Figure 1 shows the peaks obtained for several analytes (JWH-081, MN-18, 5F-APICA, JWH-182, 6-MAM, Codeine, Phenazepam, Lorazepam, Methadone, EDDP) included in the standard mixture. The gradient used ensures narrow peaks of the primary product ions for the defined retention time without any interference. The method was found to be selective for the tested compounds. The only exceptions are 3-MMC and 4-MMC, which do not separate on analysis. No interfering peaks were observed in the drug-free hair samples.

To check the stability, analyses of archival samples were performed. Four of the validated compounds did not meet the above criteria. The remaining 513 were included in the routine analysis of hair samples in our laboratory.

The developed method was verified by proficiency tests carried out by LGC Standards. During proficiency tests performed a quantitative analysis of two samples. In the first sample, the analyzed substances were Phenazepam, Morphine, 6MAM, and Codeine. In the second sample, however, these were Methadone, EDDP, Methamphetamine, and Lorazepam. The obtained results met the criteria of proficiency tests.

## Conclusions

The developed analytical method allowed us to introduce the analysis of hair samples to routine analyses in our laboratory. Use of methanol in the extraction process brought satisfactory results. With this procedure, we are able isolate as many as 513 psychoactive substances from hair samples. The performed validation has passed international proficiency tests. Due to the expected or required concentration levels in real samples, the method was developed with a division into one for synthetic cannabinoids with a lower LOQ and the other for analytes with a higher LOQ. Several of the analyzed substances did not meet the imposed validation requirements (FUB-PB-22, 2C-D, Oxymorphone, Stanozolol). This can be due to both the extraction process and the chromatographic separation method. Substances that did not meet the requirements were not included in the routine analyses, but the number of substances that can be detected with the new method is large enough to meet the current needs. However, it is essential to refine the method so that substances that have not been validated can be determined with the modified method. The method can be developed further to cover new psychoactive substances as they appear on the market. Analyzing hair samples is an excellent alternative to the commonly used matrices such as blood or urine, particularly as it also enables retrospective analyses.

## Supplementary Information

Below is the link to the electronic supplementary material.

Supplementary file1 (DOCX 353 KB)

## Data Availability

The data that support the findings of this study are available on request from the corresponding author.
