# Supplementary material for: One-step extraction and determination of 513 psychoactive substances, drugs, and their metabolites from hair by LC–MS/MS
Source: Arch Toxicol. 2022 Aug 25;96(11):2927–33. doi: 10.1007/s00204-022-03343-w (PMC9525419; doi:10.1007/s00204-022-03343-w)
Supplement: Supplementary file 1 — Supplementary file1 (DOCX 353 KB) [file 204_2022_3343_MOESM1_ESM.docx]

**Electronic Supplementary Materials**

***for***

**One-step extraction and determination of 513 psychoactive substances, drugs, and their metabolites from hair by LC-MS/MS**

Jadwiga Musiał^1,2*)^, Jolanta Powierska-Czarny^1)^, Jakub Czarny^1)^, Michał Raczkowski^1)^, Natalia Galant^1)^, Bogusław Buszewski^2)^, Renata Gadzała-Kopciuch^2*)^

*^1)^ Institute of Forensic Genetics, Al. Mickiewicza 3/4, 85-071 Bydgoszcz, Poland*

*^2)^Department of Environmental Chemistry and Bioanalytics, Faculty of Chemistry,*

*Nicolaus Copernicus University in Toruń, 7 Gagarin St., 87-100 Toruń, Poland*

[**j.musial@doktorant.umk.pl*](mailto:*j.musial@doktorant.umk.pl)*, *rgadz@umk.pl*

Table S1. Summary of operating parameters LC-MS/MS for identification of analytes, where: Q1 – precursor ion, Q3 – product ion, **t_R_** - retention time, DP - declustering potential, CE – collision energy, CXP - collision cell exit potential**.**

| **Q1**  **[M-H] ^+^ (m/z)** | **Q3**  **(m/z)** | **t_R_ (min)** | **Analyte** | **DP (V)** | **CE (V)** | **CXP (V)** |
| --- | --- | --- | --- | --- | --- | --- |
| 193.263 | 120.1 | 5.30 | 1-(2-METHOXYPHENYL)PIPERAZINE 1 | 106 | 43 | 8 |
| 193.263 | 150.1 | 5.30 | 1-(2-METHOXYPHENYL)PIPERAZINE 2 | 106 | 25 | 18 |
| 231.278 | 188 | 7.95 | 1-(3-TRIFLUOROMETHYLPHENYL)PIPERAZINE (TFMPP) 1 | 141 | 31 | 22 |
| 231.278 | 118 | 7.95 | 1-(3-TRIFLUOROMETHYLPHENYL)PIPERAZINE (TFMPP) 2 | 141 | 51 | 16 |
| 197.469 | 119.1 | 6.67 | 1-(4-CHLOROPHENYL)PIPERAZINE (pCPP) 1 | 121 | 33 | 14 |
| 197.469 | 118.1 | 6.67 | 1-(4-CHLOROPHENYL)PIPERAZINE (pCPP) 2 | 121 | 45 | 16 |
| 181.26 | 138.1 | 4.69 | 1-(4-FLUOROPHENYL)PIPERAZINE (FPP) 1 | 106 | 27 | 16 |
| 181.26 | 74 | 4.69 | 1-(4-FLUOROPHENYL)PIPERAZINE (FPP) 2 | 106 | 103 | 12 |
| 267.107 | 91 | 7.73 | 1.4-DIBENZYLPIPERAZINE (DBZP) 1 | 111 | 47 | 12 |
| 267.107 | 65.1 | 7.73 | 1.4-DIBENZYLPIPERAZINE (DBZP) 2 | 111 | 91 | 10 |
| 134.019 | 117.1 | 4.09 | 1-AMINOINDAN 1 | 56 | 15 | 14 |
| 134.019 | 115.1 | 4.09 | 1-AMINOINDAN 2 | 56 | 33 | 14 |
| 191.061 | 91 | 3.20 | 1-METHYL-4-BENZYLPIPERAZINE (MBZP) 1 | 86 | 31 | 12 |
| 191.061 | 65 | 3.20 | 1-METHYL-4-BENZYLPIPERAZINE (MBZP) 2 | 86 | 63 | 10 |
| 194.261 | 163 | 6.03 | 1-METHYLAMINO-1-(3.4-METHYLENEDIOXYPHENYL)PROPANE 1 | 61 | 13 | 22 |
| 194.261 | 105.1 | 6.03 | 1-METHYLAMINO-1-(3.4-METHYLENEDIOXYPHENYL)PROPANE 2 | 61 | 33 | 16 |
| 230.937 | 188 | 8.52 | 2.3-DICHLOROPHENYLPIPERAZINE (DCPP) 1 | 91 | 27 | 24 |
| 230.937 | 152 | 8.52 | 2.3-DICHLOROPHENYLPIPERAZINE (DCPP) 2 | 91 | 45 | 20 |
| 206.062 | 188.1 | 7.43 | 2.3-DIMETHYLETHCATHINONE (2.3-DMEC) 1 | 91 | 17 | 24 |
| 206.062 | 158.1 | 7.43 | 2.3-DIMETHYLETHCATHINONE (2.3-DMEC) 2 | 91 | 39 | 20 |
| 192.202 | 174 | 7.02 | 2.3-DIMETHYLMETHCATHINONE (2.3-DMMC) 1 | 66 | 17 | 20 |
| 192.202 | 159.1 | 7.02 | 2.3-DIMETHYLMETHCATHINONE (2.3-DMMC) 2 | 66 | 27 | 20 |
| 222.247 | 174.1 | 5.43 | 2.3-ETHYLONE ISOMER 1 | 86 | 23 | 8 |
| 222.247 | 146.1 | 5.43 | 2.3-ETHYLONE ISOMER 2 | 86 | 35 | 8 |
| 194.351 | 135 | 5.69 | 2.3-MDMA 1 | 91 | 25 | 18 |
| 194.351 | 77 | 5.69 | 2.3-MDMA 2 | 91 | 53 | 12 |
| 276.236 | 135 | 7.57 | 2.3-MDPV 1 | 91 | 31 | 16 |
| 276.236 | 126.1 | 7.57 | 2.3-MDPV 2 | 91 | 37 | 14 |
| 226.297 | 209.1 | 6.18 | 2.4.5-TRIMETHOXYAMPHETAMINE 1 | 76 | 15 | 10 |
| 226.297 | 179.1 | 6.18 | 2.4.5-TRIMETHOXYAMPHETAMINE 2 | 76 | 35 | 10 |
| 192.19 | 174.1 | 7.46 | 2.4-DIMETHYLMETHCATHINONE (2.4-DMMC) 1 | 76 | 17 | 20 |
| 192.19 | 159 | 7.46 | 2.4-DIMETHYLMETHCATHINONE (2.4-DMMC) 2 | 76 | 27 | 18 |
| 206.063 | 188.1 | 7.92 | 2.4-DMEC 1 | 41 | 17 | 12 |
| 206.063 | 158.1 | 7.92 | 2.4-DMEC 2 | 41 | 47 | 20 |
| 210.264 | 151.1 | 6.71 | 2.5-DMMA 1 | 56 | 23 | 20 |
| 210.264 | 121.1 | 6.71 | 2.5-DMMA 2 | 56 | 35 | 16 |
| 368.124 | 243 | 10.15 | 25B-NBF 1 | 106 | 27 | 12 |
| 370.116 | 245 | 10.15 | 25B-NBF 2 | 131 | 27 | 12 |
| 324.164 | 199 | 9.89 | 25C-NBF 1 | 111 | 25 | 10 |
| 324.164 | 184 | 9.89 | 25C-NBF 2 | 111 | 37 | 10 |
| 322.138 | 199 | 9.72 | 25C-NBOH 1 | 101 | 27 | 24 |
| 322.138 | 77 | 9.72 | 25C-NBOH 2 | 101 | 85 | 12 |
| 336.16 | 121.1 | 10.25 | 25C-NBOMe 1 | 96 | 25 | 14 |
| 336.16 | 91.1 | 10.25 | 25C-NBOMe 2 | 96 | 59 | 12 |
| 316.169 | 91 | 10.44 | 25D-NBOMe 1 | 81 | 57 | 12 |
| 316.169 | 121 | 10.44 | 25D-NBOMe 2 | 81 | 25 | 16 |
| 330.231 | 91 | 11.26 | 25E-NBOMe 1 | 106 | 61 | 12 |
| 330.231 | 121.1 | 11.26 | 25E-NBOMe 2 | 106 | 27 | 16 |
| 330.142 | 91 | 11.04 | 25G-NBOMe 1 | 81 | 61 | 12 |
| 330.142 | 121.1 | 11.04 | 25G-NBOMe 2 | 81 | 27 | 14 |
| 302.346 | 91 | 9.40 | 25H-NBOMe 1 | 81 | 55 | 12 |
| 302.346 | 121.1 | 9.40 | 25H-NBOMe 2 | 81 | 23 | 18 |
| 428.183 | 91 | 10.88 | 25-I-NB2OMe 1 | 121 | 75 | 12 |
| 428.183 | 121 | 10.88 | 25-I-NB2OMe 2 | 121 | 27 | 16 |
| 428.176 | 121.1 | 10.82 | 25I-NB3OMe 1 | 131 | 33 | 14 |
| 428.176 | 91.1 | 10.82 | 25I-NB3OMe 2 | 131 | 75 | 12 |
| 428.177 | 121.1 | 10.75 | 25I-NB4OMe 1 | 91 | 21 | 16 |
| 428.177 | 78 | 10.75 | 25I-NB4OMe 2 | 91 | 113 | 10 |
| 416.016 | 290.8 | 10.58 | 25I-NBF 1 | 121 | 29 | 12 |
| 416.016 | 276 | 10.58 | 25I-NBF 2 | 121 | 43 | 14 |
| 442.154 | 135.1 | 10.71 | 25I-NBMD 1 | 116 | 31 | 16 |
| 442.154 | 77 | 10.71 | 25I-NBMD 2 | 116 | 93 | 12 |
| 414.121 | 291 | 10.42 | 25I-NBOH 1 | 101 | 31 | 12 |
| 414.121 | 307.9 | 10.42 | 25I-NBOH 2 | 101 | 23 | 14 |
| 347.2 | 91 | 9.05 | 25N-NBOMe 1 | 86 | 59 | 12 |
| 347.2 | 121.1 | 9.05 | 25N-NBOMe 2 | 86 | 23 | 16 |
| 362.264 | 91 | 10.77 | 25T2-NBOMe 1 | 121 | 59 | 10 |
| 362.264 | 121.1 | 10.77 | 25T2-NBOMe 2 | 121 | 27 | 14 |
| 348.035 | 91.1 | 10.05 | 25T-NBOMe 1 | 96 | 63 | 14 |
| 348.035 | 121.1 | 10.05 | 25T-NBOMe 2 | 96 | 27 | 16 |
| 150.209 | 91 | 6.08 | 2-AMINO-1-PHENYLBUTANE 1 | 61 | 23 | 12 |
| 150.209 | 65 | 6.08 | 2-AMINO-1-PHENYLBUTANE 2 | 61 | 49 | 10 |
| 134.257 | 117.1 | 3.87 | 2-AMINOINDANE 1 | 51 | 19 | 14 |
| 134.257 | 115.1 | 3.87 | 2-AMINOINDANE 2 | 51 | 33 | 14 |
| 214.186 | 169 | 7.04 | 2-BROMOAMPHETAMINE 1 | 61 | 27 | 8 |
| 216.192 | 170.9 | 7.04 | 2-BROMOAMPHETAMINE 2 | 56 | 27 | 20 |
| 228.188 | 169 | 7.10 | 2-BROMOMETHAMPHETAMINE 1 | 71 | 29 | 20 |
| 230.177 | 171 | 7.10 | 2-BROMOMETHAMPHETAMINE 2 | 71 | 27 | 10 |
| 259.957 | 243 | 7.70 | 2C-B 1 | 51 | 17 | 12 |
| 259.957 | 227.9 | 7.70 | 2C-B 2 | 51 | 29 | 10 |
| 283.976 | 267 | 7.99 | 2C-B_FLY 1 | 106 | 21 | 12 |
| 283.976 | 188.1 | 7.99 | 2C-B_FLY 2 | 106 | 33 | 24 |
| 216.441 | 199 | 7.28 | 2C-C 1 | 76 | 15 | 24 |
| 216.441 | 184 | 7.28 | 2C-C 2 | 76 | 27 | 22 |
| 195.583 | 179 | 7.50 | 2C-D 1 | 71 | 15 | 24 |
| 195.583 | 164.1 | 7.50 | 2C-D 2 | 71 | 25 | 20 |
| 210.052 | 178 | 8.54 | 2C-G 1 | 76 | 23 | 22 |
| 210.052 | 163 | 8.54 | 2C-G 2 | 76 | 37 | 20 |
| 170.196 | 125 | 6.56 | 2-CHLOROAMPHETAMINE 1 | 61 | 25 | 16 |
| 170.196 | 89 | 6.56 | 2-CHLOROAMPHETAMINE 2 | 61 | 51 | 14 |
| 307.947 | 291 | 8.39 | 2C-I 1 | 66 | 19 | 12 |
| 307.947 | 276 | 8.39 | 2C-I 2 | 66 | 31 | 12 |
| 224.065 | 207.1 | 10.09 | 2C-P 1 | 66 | 15 | 10 |
| 224.065 | 192.1 | 10.09 | 2C-P 2 | 66 | 25 | 24 |
| 256.022 | 239.1 | 9.58 | 2C-T-7 1 | 71 | 17 | 10 |
| 256.022 | 91.1 | 9.58 | 2C-T-7 2 | 71 | 63 | 12 |
| 250.269 | 233.1 | 8.67 | 2C-TFM 1 | 91 | 17 | 10 |
| 250.269 | 218 | 8.67 | 2C-TFM 2 | 91 | 29 | 12 |
| 196.255 | 148.1 | 4.67 | 2-FEC 1 | 76 | 41 | 18 |
| 196.255 | 135.1 | 4.67 | 2-FEC 2 | 76 | 37 | 10 |
| 168.199 | 123.1 | 3.17 | 2-FIC 1 | 61 | 21 | 14 |
| 168.199 | 103.1 | 3.17 | 2-FIC 2 | 61 | 33 | 14 |
| 154.13 | 109 | 5.10 | 2-FLUOROAMPHETAMINE 1 | 61 | 23 | 14 |
| 154.13 | 137.1 | 5.10 | 2-FLUOROAMPHETAMINE 2 | 61 | 13 | 16 |
| 168.247 | 109.1 | 5.35 | 2-FLUOROMETHAMPHETAMINE (2-FMA) 1 | 61 | 25 | 12 |
| 168.247 | 83.1 | 5.35 | 2-FLUOROMETHAMPHETAMINE (2-FMA) 2 | 61 | 53 | 12 |
| 182.187 | 164.1 | 4.02 | 2-FLUOROMETHCATHINONE (2-FMC) 1 | 81 | 19 | 22 |
| 182.187 | 149 | 4.02 | 2-FLUOROMETHCATHINONE (2-FMC) 2 | 81 | 29 | 16 |
| 262.178 | 216.9 | 7.76 | 2-IODOAMPHETAMINE 1 | 81 | 27 | 12 |
| 262.178 | 90 | 7.76 | 2-IODOAMPHETAMINE 2 | 81 | 47 | 12 |
| 190.239 | 58 | 7.05 | 2-MAPB 1 | 51 | 19 | 8 |
| 190.239 | 91.1 | 7.05 | 2-MAPB 2 | 51 | 47 | 14 |
| 194.219 | 176.1 | 5.30 | 2-MeOMC 1 | 61 | 17 | 10 |
| 194.219 | 161.1 | 5.30 | 2-MeOMC 2 | 61 | 27 | 16 |
| 152.214 | 120.1 | 4.95 | 2-METHOXY-2-PHENYLETHYLAMINE 1 | 61 | 15 | 14 |
| 152.214 | 77 | 4.95 | 2-METHOXY-2-PHENYLETHYLAMINE 2 | 61 | 45 | 12 |
| 166.068 | 121.1 | 6.16 | 2-METHOXYAMPHETAMINE (2-MA) 1 | 56 | 21 | 16 |
| 166.068 | 149 | 6.16 | 2-METHOXYAMPHETAMINE (2-MA) 2 | 56 | 13 | 18 |
| 180.322 | 120.9 | 6.34 | 2-METHOXYMETHAMPHETAMINE (2-MeOMA) 1 | 66 | 23 | 14 |
| 180.322 | 91.1 | 6.34 | 2-METHOXYMETHAMPHETAMINE (2-MeOMA) 2 | 66 | 39 | 8 |
| 164.237 | 91 | 6.20 | 2-METHYLAMINO-1-PHENYLBUTANE 1 | 71 | 25 | 14 |
| 164.237 | 65 | 6.20 | 2-METHYLAMINO-1-PHENYLBUTANE 2 | 71 | 55 | 8 |
| 178.06 | 160.1 | 5.73 | 2-METHYLMETHCATHINONE (2-MMC) 1 | 76 | 17 | 22 |
| 178.06 | 145.1 | 5.73 | 2-METHYLMETHCATHINONE (2-MMC) 2 | 76 | 27 | 18 |
| 232.265 | 105.1 | 7.34 | 2-METHYL-PBP 1 | 116 | 33 | 12 |
| 232.265 | 91 | 7.34 | 2-METHYL-PBP 2 | 116 | 53 | 12 |
| 218.291 | 98.1 | 6.47 | 2-METHYL-PPP 1 | 116 | 31 | 16 |
| 218.291 | 119.1 | 6.47 | 2-METHYL-PPP 2 | 116 | 31 | 8 |
| 303.454 | 84.1 | 9.94 | 3.4-DICHLOROMETHYLPHENIDATE (3.4-CTMP) 1 | 121 | 27 | 12 |
| 303.454 | 56 | 9.94 | 3.4-DICHLOROMETHYLPHENIDATE (3.4-CTMP) 2 | 121 | 77 | 8 |
| 292.246 | 151 | 7.44 | 3.4-DIMETHOXY-ALPHA-PVP 1 | 116 | 35 | 22 |
| 292.246 | 126.1 | 7.44 | 3.4-DIMETHOXY-ALPHA-PVP 2 | 116 | 33 | 12 |
| 192.031 | 174.1 | 7.32 | 3.4-DIMETHYLMETHCATHINONE (3.4-DMMC) 1 | 81 | 17 | 20 |
| 192.031 | 159.2 | 7.32 | 3.4-DIMETHYLMETHCATHINONE (3.4-DMMC) 2 | 81 | 27 | 18 |
| 206.146 | 158.1 | 7.74 | 3.4-DMEC 1 | 86 | 43 | 14 |
| 206.146 | 115.1 | 7.74 | 3.4-DMEC 2 | 86 | 67 | 6 |
| 210.063 | 179.1 | 5.21 | 3.4-DMMA 1 | 61 | 17 | 22 |
| 210.063 | 151.1 | 5.21 | 3.4-DMMA 2 | 61 | 29 | 20 |
| 208.248 | 177.1 | 5.61 | 3.4-EDMA 1 | 61 | 19 | 10 |
| 208.248 | 149.1 | 5.61 | 3.4-EDMA 2 | 61 | 29 | 20 |
| 222.191 | 204 | 5.23 | 3.4-EDMC 1 | 61 | 19 | 10 |
| 222.191 | 189.1 | 5.23 | 3.4-EDMC 2 | 61 | 29 | 10 |
| 194.025 | 163.1 | 5.27 | 3.4-MDMA (ECSTAZY) 1 | 76 | 17 | 20 |
| 194.025 | 105.1 | 5.27 | 3.4-MDMA (ECSTAZY) 2 | 76 | 33 | 14 |
| 222.051 | 163 | 6.74 | 3.4-MDPA 1 | 81 | 19 | 20 |
| 222.051 | 105.1 | 6.74 | 3.4-MDPA 2 | 81 | 35 | 14 |
| 290.285 | 135.1 | 8.66 | 3.4-MDPHP 1 | 131 | 35 | 16 |
| 290.285 | 140.2 | 8.66 | 3.4-MDPHP 2 | 131 | 35 | 18 |
| 276.144 | 126.1 | 7.50 | 3.4-METHYLENDIOXYPYROVALERONE 1 | 76 | 31 | 12 |
| 276.144 | 135.1 | 7.50 | 3.4-METHYLENDIOXYPYROVALERONE 2 | 76 | 41 | 8 |
| 318.23 | 135.1 | 10.83 | 3.4-METHYLENEDIOXY_PV9 1 | 166 | 35 | 8 |
| 318.23 | 168.2 | 10.83 | 3.4-METHYLENEDIOXY_PV9 2 | 166 | 39 | 20 |
| 396.257 | 181.1 | 9.73 | 30C-NBOMe 1 | 61 | 23 | 10 |
| 396.257 | 148.1 | 9.73 | 30C-NBOMe 2 | 61 | 59 | 18 |
| 214.192 | 168.9 | 7.31 | 3-BROMOAMPHETAMINE 1 | 46 | 25 | 20 |
| 216.189 | 171.1 | 7.31 | 3-BROMOAMPHETAMINE 2 | 66 | 25 | 14 |
| 228.18 | 169 | 7.38 | 3-BROMOMETHAMPHETAMINE 1 | 86 | 27 | 22 |
| 230.202 | 170.9 | 7.38 | 3-BROMOMETHAMPHETAMINE 2 | 66 | 27 | 20 |
| 244.143 | 145 | 6.64 | 3-BROMOMETHCATHINONE (3-BMC) 1 | 86 | 23 | 20 |
| 242.128 | 145.1 | 6.64 | 3-BROMOMETHCATHINONE (3-BMC) 2 | 96 | 25 | 22 |
| 383.161 | 239 | 15.28 | 3-CAF 1 | 101 | 19 | 12 |
| 383.161 | 210 | 15.28 | 3-CAF 2 | 101 | 65 | 24 |
| 298.069 | 280.9 | 8.54 | 3C-B-FLY 1 | 66 | 21 | 12 |
| 300.079 | 283 | 8.54 | 3C-B-FLY 2 | 46 | 17 | 16 |
| 170.206 | 125.1 | 6.83 | 3-CHLOROAMPHETAMINE 1 | 66 | 25 | 14 |
| 170.206 | 89 | 6.83 | 3-CHLOROAMPHETAMINE 2 | 66 | 53 | 12 |
| 198.231 | 145.1 | 5.95 | 3-CHLOROMETHCATHINONE (3-CMC) 1 | 76 | 25 | 18 |
| 198.231 | 144.1 | 5.95 | 3-CHLOROMETHCATHINONE (3-CMC) 2 | 76 | 41 | 18 |
| 197.249 | 154.1 | 6.78 | 3-CHLOROPHENYLPIPERAZINE (mCPP) 1 | 101 | 27 | 18 |
| 197.249 | 118.1 | 6.78 | 3-CHLOROPHENYLPIPERAZINE (mCPP) 2 | 101 | 47 | 14 |
| 254.286 | 195.1 | 8.42 | 3C-P 1 | 56 | 19 | 10 |
| 254.286 | 107.1 | 8.42 | 3C-P 2 | 56 | 35 | 14 |
| 274.249 | 126.1 | 7.99 | 3-DESOXY-3.4-MDPV 1 | 126 | 31 | 18 |
| 274.249 | 133.1 | 7.99 | 3-DESOXY-3.4-MDPV 2 | 126 | 37 | 16 |
| 192.22 | 174.1 | 7.45 | 3-ETHYLMETHCATHINONE (3-EMC) 1 | 66 | 17 | 22 |
| 192.22 | 144.1 | 7.45 | 3-ETHYLMETHCATHINONE (3-EMC) 2 | 66 | 43 | 18 |
| 196.262 | 135.1 | 4.96 | 3-FEC 1 | 81 | 39 | 18 |
| 196.262 | 148.1 | 4.96 | 3-FEC 2 | 81 | 41 | 18 |
| 222.28 | 123.1 | 5.30 | 3-FLUORO-ALFA-PPP 1 | 86 | 31 | 12 |
| 222.28 | 98.1 | 5.30 | 3-FLUORO-ALFA-PPP 2 | 86 | 33 | 14 |
| 154.134 | 109 | 5.10 | 3-FLUOROAMPHETAMINE 1 | 76 | 25 | 14 |
| 154.134 | 137.1 | 5.10 | 3-FLUOROAMPHETAMINE 2 | 76 | 13 | 16 |
| 168.223 | 109.1 | 5.35 | 3-FLUOROMETHAMPHETAMINE (3-FMA) 1 | 76 | 27 | 18 |
| 168.223 | 83.1 | 5.35 | 3-FLUOROMETHAMPHETAMINE (3-FMA) 2 | 76 | 53 | 10 |
| 182.014 | 164.1 | 4.38 | 3-FLUOROMETHCATHINONE (3-FMC) 1 | 81 | 19 | 20 |
| 182.014 | 149.1 | 4.38 | 3-FLUOROMETHCATHINONE (3-FMC) 2 | 81 | 27 | 18 |
| 331.895 | 286.9 | 9.11 | 3-HYDROXYBROMAZEPAM 1 | 106 | 27 | 12 |
| 331.895 | 314.9 | 9.11 | 3-HYDROXYBROMAZEPAM 2 | 106 | 21 | 14 |
| 330.031 | 238 | 10.15 | 3-HYDROXYFLUNITRAZEPAM 1 | 96 | 43 | 12 |
| 330.031 | 284 | 10.15 | 3-HYDROXYFLUNITRAZEPAM 2 | 96 | 27 | 12 |
| 262.179 | 217 | 8.06 | 3-IODOAMPHETAMINE 1 | 66 | 25 | 10 |
| 262.179 | 117.1 | 8.06 | 3-IODOAMPHETAMINE 2 | 66 | 43 | 14 |
| 192.185 | 144.2 | 6.44 | 3-MEC 1 | 86 | 39 | 16 |
| 192.185 | 91.1 | 6.44 | 3-MEC 2 | 86 | 47 | 14 |
| 194.225 | 161.1 | 5.30 | 3-MeOMC 1 | 81 | 25 | 20 |
| 194.225 | 118.1 | 5.30 | 3-MeOMC 2 | 81 | 47 | 12 |
| 166.07 | 121.1 | 5.59 | 3-METHOXYAMPHETAMINE (3-MA) 1 | 41 | 21 | 16 |
| 166.07 | 91 | 5.59 | 3-METHOXYAMPHETAMINE (3-MA) 2 | 41 | 37 | 14 |
| 274.19 | 86.1 | 8.92 | 3-METHOXYPHENCYCLIDINE 1 | 46 | 17 | 10 |
| 274.19 | 121.1 | 8.92 | 3-METHOXYPHENCYCLIDINE 2 | 46 | 37 | 14 |
| 178.046 | 160.1 | 5.92 | 3-METHYLMETHCATHINONE (3-MMC) 1 | 86 | 17 | 20 |
| 178.046 | 145.1 | 5.92 | 3-METHYLMETHCATHINONE (3-MMC) 2 | 86 | 27 | 18 |
| 232.266 | 105.1 | 7.47 | 3-METHYL-PBP 1 | 106 | 31 | 14 |
| 232.266 | 91 | 7.47 | 3-METHYL-PBP 2 | 106 | 55 | 12 |
| 218.209 | 119.3 | 6.64 | 3-METHYL-PPP 1 | 116 | 31 | 6 |
| 218.209 | 91 | 6.64 | 3-METHYL-PPP 2 | 116 | 49 | 8 |
| 191.255 | 148.1 | 7.31 | 4.4'-DMAR 1 | 61 | 17 | 18 |
| 191.255 | 91 | 7.31 | 4.4'-DMAR 2 | 61 | 39 | 12 |
| 275.216 | 86 | 6.36 | 4-AcO-DET 1 | 81 | 23 | 12 |
| 275.216 | 160 | 6.36 | 4-AcO-DET 2 | 81 | 35 | 20 |
| 247.314 | 58.1 | 5.45 | 4-AcO-DMT 1 | 81 | 21 | 6 |
| 247.314 | 160.1 | 5.45 | 4-AcO-DMT 2 | 81 | 33 | 16 |
| 261.3 | 72 | 5.92 | 4-AcO-MET 1 | 76 | 21 | 10 |
| 261.3 | 160 | 5.92 | 4-AcO-MET 2 | 76 | 35 | 22 |
| 176.148 | 131.1 | 6.67 | 4-APB 1 | 66 | 25 | 16 |
| 176.148 | 91 | 6.67 | 4-APB 2 | 66 | 39 | 12 |
| 178.238 | 161.1 | 5.56 | 4-APDB 1 | 71 | 13 | 22 |
| 178.238 | 133.1 | 5.56 | 4-APDB 2 | 71 | 25 | 16 |
| 288.233 | 257 | 8.31 | 4-BROMO-2.5-DMMA 1 | 61 | 19 | 12 |
| 290.24 | 259 | 8.31 | 4-BROMO-2.5-DMMA 2 | 56 | 19 | 12 |
| 214.182 | 169 | 7.44 | 4-BROMOAMPHETAMINE 1 | 76 | 25 | 20 |
| 216.181 | 170.9 | 7.44 | 4-BROMOAMPHETAMINE 2 | 56 | 25 | 22 |
| 228.205 | 168.9 | 7.52 | 4-BROMOMETHAMPHETAMINE 1 | 91 | 29 | 20 |
| 230.2 | 170.9 | 7.52 | 4-BROMOMETHAMPHETAMINE 2 | 91 | 27 | 20 |
| 243.495 | 145.1 | 6.64 | 4-BROMOMETHCATHINONE (BREFEDRONE) 1 | 101 | 23 | 18 |
| 243.495 | 144.1 | 6.64 | 4-BROMOMETHCATHINONE (BREFEDRONE) 2 | 101 | 45 | 18 |
| 184.174 | 125.1 | 7.98 | 4-CAB 1 | 46 | 25 | 16 |
| 184.174 | 89 | 7.98 | 4-CAB 2 | 46 | 57 | 14 |
| 212.21 | 159.3 | 6.65 | 4-CEC 1 | 66 | 25 | 8 |
| 212.21 | 144.1 | 6.65 | 4-CEC 2 | 66 | 39 | 18 |
| 238.267 | 139.1 | 6.85 | 4-CHLORO-ALPHA-PPP 1 | 111 | 33 | 16 |
| 238.267 | 98.1 | 6.85 | 4-CHLORO-ALPHA-PPP 2 | 111 | 33 | 12 |
| 266.3 | 125.1 | 8.93 | 4-CHLORO-ALPHA-PVP 1 | 101 | 33 | 10 |
| 266.3 | 126.1 | 8.93 | 4-CHLORO-ALPHA-PVP 2 | 101 | 37 | 18 |
| 170.197 | 125 | 6.93 | 4-CHLOROAMPHETAMINE 1 | 51 | 25 | 12 |
| 170.197 | 89 | 6.93 | 4-CHLOROAMPHETAMINE 2 | 51 | 53 | 12 |
| 198 | 145.1 | 6.15 | 4-CHLOROMETHCATHINONE (4-CMC) 1 | 76 | 25 | 18 |
| 198.008 | 144.1 | 6.15 | 4-CHLOROMETHCATHINONE (4-CMC) 2 | 71 | 41 | 18 |
| 184.235 | 125.1 | 7.05 | 4-CMA 1 | 81 | 29 | 14 |
| 184.235 | 89 | 7.05 | 4-CMA 2 | 81 | 57 | 12 |
| 204.264 | 131.1 | 7.34 | 4-EAPB 1 | 76 | 29 | 10 |
| 204.264 | 91 | 7.34 | 4-EAPB 2 | 76 | 45 | 12 |
| 206.207 | 133.2 | 7.50 | 4-ETHYL-N.N-DMC 1 | 61 | 27 | 14 |
| 206.207 | 105.2 | 7.50 | 4-ETHYL-N.N-DMC 2 | 61 | 35 | 18 |
| 196.26 | 148.1 | 4.97 | 4-FEC 1 | 71 | 41 | 18 |
| 196.26 | 135.1 | 4.97 | 4-FEC 2 | 71 | 37 | 16 |
| 196.234 | 149.1 | 5.73 | 4-FLUORO BUPHEDRONE 1 | 76 | 31 | 18 |
| 196.234 | 148.1 | 5.73 | 4-FLUORO BUPHEDRONE 2 | 76 | 47 | 18 |
| 210.264 | 109 | 7.20 | 4-FLUORO PENTEDRONE 1 | 86 | 33 | 14 |
| 210.264 | 74 | 7.20 | 4-FLUORO PENTEDRONE 2 | 86 | 111 | 10 |
| 278.165 | 109 | 10.00 | 4-FLUORO PV8 1 | 126 | 33 | 16 |
| 278.165 | 95.1 | 10.00 | 4-FLUORO PV8 2 | 126 | 71 | 12 |
| 292.257 | 109.1 | 11.02 | 4-FLUORO PV9 1 | 116 | 35 | 14 |
| 292.257 | 95 | 11.02 | 4-FLUORO PV9 2 | 116 | 71 | 14 |
| 222.267 | 123.1 | 5.30 | 4-FLUORO-ALFA-PPP 1 | 86 | 31 | 8 |
| 222.267 | 98.1 | 5.30 | 4-FLUORO-ALFA-PPP 2 | 86 | 33 | 14 |
| 168.214 | 109.1 | 5.34 | 4-FLUOROMETHAMPHETAMINE (4-FMA) 1 | 81 | 27 | 12 |
| 168.214 | 137 | 5.34 | 4-FLUOROMETHAMPHETAMINE (4-FMA) 2 | 81 | 15 | 18 |
| 182.011 | 164.1 | 4.37 | 4-FLUOROMETHCATHINONE (4-FMC) 1 | 71 | 19 | 22 |
| 182.011 | 149 | 4.37 | 4-FLUOROMETHCATHINONE (4-FMC) 2 | 71 | 29 | 20 |
| 250.25 | 109 | 7.55 | 4F-PVP 1 | 91 | 31 | 12 |
| 250.25 | 126.1 | 7.55 | 4F-PVP 2 | 91 | 35 | 16 |
| 262.005 | 161.1 | 6.30 | 4-HYDROXY DiPT 1 | 171 | 31 | 20 |
| 262.005 | 114.1 | 6.30 | 4-HYDROXY DiPT 2 | 171 | 21 | 14 |
| 341.932 | 325 | 8.80 | 4-HYDROXYMIDAZOLAM 1 | 121 | 31 | 14 |
| 341.932 | 297 | 8.80 | 4-HYDROXYMIDAZOLAM 2 | 121 | 41 | 14 |
| 262.177 | 245 | 8.19 | 4-IODOAMPHETAMINE 1 | 56 | 15 | 12 |
| 262.177 | 216.9 | 8.19 | 4-IODOAMPHETAMINE 2 | 56 | 27 | 12 |
| 190.254 | 131.1 | 6.84 | 4-MAPB 1 | 76 | 27 | 18 |
| 190.254 | 91 | 6.84 | 4-MAPB 2 | 76 | 41 | 10 |
| 220.297 | 144.1 | 8.56 | 4-MEAP 1 | 66 | 43 | 18 |
| 220.297 | 105.1 | 8.56 | 4-MEAP 2 | 66 | 29 | 12 |
| 262.212 | 121.1 | 8.02 | 4-MEO-ALPHA-PVP 1 | 91 | 33 | 12 |
| 262.212 | 191.2 | 8.02 | 4-MEO-ALPHA-PVP 2 | 91 | 25 | 18 |
| 248.282 | 112.1 | 6.90 | 4-MeOPBP 1 | 101 | 31 | 14 |
| 248.282 | 121.1 | 6.90 | 4-MeOPBP 2 | 101 | 37 | 16 |
| 274.176 | 121.1 | 8.92 | 4-METHOXY PHENCYCLIDINE 1 | 61 | 39 | 14 |
| 274.176 | 189.1 | 8.92 | 4-METHOXY PHENCYCLIDINE 2 | 61 | 17 | 10 |
| 290.295 | 121.1 | 10.17 | 4-METHOXY PV8 1 | 126 | 35 | 16 |
| 290.295 | 154.2 | 10.17 | 4-METHOXY PV8 2 | 126 | 33 | 8 |
| 304.286 | 121.1 | 11.12 | 4-METHOXY PV9 1 | 101 | 35 | 14 |
| 304.286 | 168.2 | 11.12 | 4-METHOXY PV9 2 | 101 | 35 | 8 |
| 180.289 | 149 | 5.58 | 4-METHOXYMETHAMPHETAMINE (PMMA) 1 | 66 | 15 | 20 |
| 180.289 | 121.1 | 5.58 | 4-METHOXYMETHAMPHETAMINE (PMMA) 2 | 66 | 27 | 14 |
| 206.274 | 144.1 | 8.28 | 4-METHYL PENTEDRONE 1 | 86 | 47 | 18 |
| 206.274 | 105.1 | 8.28 | 4-METHYL PENTEDRONE 2 | 86 | 29 | 14 |
| 206.11 | 188.3 | 7.43 | 4-METHYL-ALPHA-ETHYLAMINOBUTIOPHENONE 1 | 56 | 19 | 22 |
| 206.11 | 144.2 | 7.43 | 4-METHYL-ALPHA-ETHYLAMINOBUTIOPHENONE 2 | 56 | 43 | 10 |
| 150.227 | 105.1 | 6.68 | 4-METHYLAMPHETAMINE 1 | 56 | 23 | 16 |
| 150.227 | 133.1 | 6.68 | 4-METHYLAMPHETAMINE 2 | 56 | 11 | 8 |
| 164.232 | 146.1 | 5.65 | 4-METHYLCATHINONE 1 | 71 | 15 | 18 |
| 164.232 | 131 | 5.65 | 4-METHYLCATHINONE 2 | 71 | 27 | 16 |
| 164.25 | 105.1 | 6.82 | 4-METHYLMETHAMPHETAMINE (4-MMA) 1 | 66 | 25 | 14 |
| 164.25 | 133.1 | 6.82 | 4-METHYLMETHAMPHETAMINE (4-MMA) 2 | 66 | 15 | 16 |
| 178.066 | 160.1 | 5.92 | 4-METHYLMETHCATHINONE (4-MMC) 1 | 71 | 17 | 20 |
| 178.066 | 145.1 | 5.92 | 4-METHYLMETHCATHINONE (4-MMC) 2 | 71 | 27 | 18 |
| 192.249 | 119.1 | 6.01 | 4-METHYL-N.N-DMC 1 | 101 | 27 | 16 |
| 192.249 | 72.1 | 6.01 | 4-METHYL-N.N-DMC 2 | 101 | 33 | 12 |
| 206.209 | 105 | 7.07 | 4-METHYL-N-METHYLBUPHEDRONE 1 | 51 | 29 | 16 |
| 206.209 | 90.9 | 7.07 | 4-METHYL-N-METHYLBUPHEDRONE 2 | 51 | 47 | 12 |
| 220.287 | 144.1 | 9.42 | 4-METHYL-N-METHYLHEXANOPHENONE 1 | 86 | 47 | 16 |
| 220.287 | 105.1 | 9.42 | 4-METHYL-N-METHYLHEXANOPHENONE 2 | 86 | 29 | 14 |
| 232.31 | 105.1 | 7.47 | 4-METHYL-PBP 1 | 81 | 33 | 14 |
| 232.31 | 91 | 7.47 | 4-METHYL-PBP 2 | 81 | 55 | 12 |
| 260.347 | 105.1 | 9.62 | 4-METHYL-PHP 1 | 131 | 33 | 14 |
| 260.347 | 91.1 | 9.62 | 4-METHYL-PHP 2 | 131 | 63 | 10 |
| 203.04 | 144.1 | 8.36 | 4-METHYL-α-ETHYLTRYPTAMINE 1 | 56 | 29 | 20 |
| 203.04 | 186 | 8.36 | 4-METHYL-α-ETHYLTRYPTAMINE 2 | 56 | 13 | 24 |
| 176.082 | 44 | 6.98 | 5-APB 1 | 46 | 23 | 8 |
| 176.082 | 91 | 6.98 | 5-APB 2 | 46 | 47 | 10 |
| 178.29 | 161 | 5.56 | 5-APDB 1 | 56 | 13 | 22 |
| 178.29 | 133.1 | 5.56 | 5-APDB 2 | 56 | 27 | 18 |
| 176.173 | 159.2 | 8.71 | 5-APDI 1 | 71 | 13 | 22 |
| 176.173 | 131.2 | 8.71 | 5-APDI 2 | 71 | 27 | 10 |
| 365.216 | 320.1 | 12.99 | 5-CHLORO AB-PINACA 1 | 91 | 21 | 14 |
| 367.248 | 322.2 | 12.99 | 5-CHLORO AB-PINACA 2 | 86 | 21 | 14 |
| 391.226 | 247.9 | 14.09 | 5-CHLORO-NNEI 1 | 86 | 31 | 12 |
| 391.226 | 144.1 | 14.09 | 5-CHLORO-NNEI 2 | 86 | 55 | 8 |
| 204.961 | 132.1 | 7.09 | 5-EAPB 1 | 86 | 29 | 16 |
| 204.961 | 131.6 | 7.09 | 5-EAPB 2 | 86 | 29 | 16 |
| 348.07 | 232.1 | 12.10 | 5F-ABICA 1 | 71 | 29 | 12 |
| 348.07 | 144.1 | 12.10 | 5F-ABICA 2 | 71 | 53 | 18 |
| 349.193 | 233.1 | 12.23 | 5F-AB-PINACA 1 | 86 | 33 | 10 |
| 349.193 | 304.1 | 12.23 | 5F-AB-PINACA 2 | 86 | 21 | 14 |
| 378.193 | 233.1 | 14.03 | 5F-ADB (5F-MDMB-PINACA) 1 | 116 | 33 | 10 |
| 378.193 | 318.2 | 14.03 | 5F-ADB (5F-MDMB-PINACA) 2 | 116 | 23 | 14 |
| 364.092 | 233.1 | 13.50 | 5F-AMB (5F-AMB-PINACA) 1 | 101 | 31 | 10 |
| 364.092 | 304.2 | 13.50 | 5F-AMB (5F-AMB-PINACA) 2 | 101 | 21 | 14 |
| 383.14 | 135.1 | 15.01 | 5F-APICA (STS-135) 1 | 156 | 39 | 16 |
| 383.14 | 77 | 15.01 | 5F-APICA (STS-135) 2 | 156 | 119 | 12 |
| 384.207 | 135.1 | 15.46 | 5F-APINACA (AKB-48-5F) 1 | 106 | 29 | 16 |
| 384.207 | 77.1 | 15.46 | 5F-APINACA (AKB-48-5F) 2 | 106 | 121 | 12 |
| 368.294 | 135.1 | 15.30 | 5-F-JWH-018 ADAMANTYL ANALOG 1 | 171 | 39 | 10 |
| 368.294 | 77 | 15.30 | 5-F-JWH-018 ADAMANTYL ANALOG 2 | 171 | 113 | 12 |
| 375.043 | 232.1 | 13.54 | 5-FLUORO MN-24 (5-FLUORO NNEI) 1 | 111 | 31 | 10 |
| 375.043 | 144.1 | 13.54 | 5-FLUORO MN-24 (5-FLUORO NNEI) 2 | 111 | 57 | 18 |
| 377.255 | 233.1 | 15.13 | 5-FLUORO THJ 1 | 91 | 29 | 12 |
| 377.255 | 145.1 | 15.13 | 5-FLUORO THJ 2 | 91 | 53 | 14 |
| 363.254 | 145.1 | 12.29 | 5-FLUORO-2-ADB-PINACA_ISOMER_2 1 | 116 | 47 | 10 |
| 363.254 | 318.1 | 12.29 | 5-FLUORO-2-ADB-PINACA_ISOMER_2 2 | 116 | 29 | 14 |
| 367.233 | 249.1 | 13.78 | 5-FLUORO-CUMYL-PICA 1 | 96 | 21 | 12 |
| 367.233 | 91 | 13.78 | 5-FLUORO-CUMYL-PICA 2 | 96 | 77 | 14 |
| 376.497 | 233.1 | 14.60 | 5-FLUORO-MN-18 1 | 126 | 23 | 10 |
| 376.497 | 145.1 | 14.60 | 5-FLUORO-MN-18 2 | 126 | 53 | 18 |
| 376.066 | 145.1 | 9.85 | 5-FLUORO-MN-21 (5-FLUORO-PCN) 1 | 201 | 55 | 18 |
| 376.066 | 117 | 9.85 | 5-FLUORO-MN-21 (5-FLUORO-PCN) 2 | 201 | 89 | 14 |
| 339.222 | 91 | 13.04 | 5-FLUORO-SDB-006 1 | 141 | 63 | 14 |
| 339.222 | 232 | 13.04 | 5-FLUORO-SDB-006 2 | 141 | 31 | 10 |
| 378.028 | 233.1 | 14.03 | 5F-NPB-22 1 | 81 | 23 | 10 |
| 378.028 | 145.1 | 14.03 | 5F-NPB-22 2 | 81 | 55 | 18 |
| 377.092 | 232.1 | 13.60 | 5F-PB-22 1 | 81 | 27 | 10 |
| 377.092 | 144.1 | 13.60 | 5F-PB-22 2 | 81 | 57 | 20 |
| 311.223 | 144.1 | 12.11 | 5-F-PENTYL-3-PYRIDINOYLINDOLE 1 | 136 | 51 | 18 |
| 311.223 | 89 | 12.11 | 5-F-PENTYL-3-PYRIDINOYLINDOLE 2 | 136 | 99 | 12 |
| 377.212 | 233.1 | 14.79 | 5F-SDB-005 1 | 121 | 15 | 10 |
| 377.212 | 145.1 | 14.79 | 5F-SDB-005 2 | 121 | 51 | 10 |
| 205.058 | 58.1 | 2.86 | 5-HYDROXY DMT 1 | 81 | 17 | 8 |
| 205.058 | 160.1 | 2.86 | 5-HYDROXY DMT 2 | 81 | 25 | 20 |
| 175.275 | 158.1 | 4.98 | 5-IT 1 | 61 | 13 | 18 |
| 175.275 | 130.1 | 4.98 | 5-IT 2 | 61 | 31 | 18 |
| 190.252 | 159.1 | 6.61 | 5-MAPB 1 | 56 | 15 | 16 |
| 190.252 | 131.1 | 6.61 | 5-MAPB 2 | 56 | 29 | 20 |
| 192.202 | 161.1 | 5.67 | 5-MAPDB 1 | 56 | 17 | 18 |
| 192.202 | 133.1 | 5.67 | 5-MAPDB 2 | 56 | 31 | 14 |
| 219.295 | 160.1 | 7.06 | 5-MeO-ALPHA-ET 1 | 71 | 27 | 18 |
| 219.295 | 117.1 | 7.06 | 5-MeO-ALPHA-ET 2 | 71 | 55 | 14 |
| 271.063 | 110.1 | 7.20 | 5-MeO-DALT 1 | 86 | 21 | 14 |
| 271.063 | 174.1 | 7.20 | 5-MeO-DALT 2 | 86 | 27 | 22 |
| 205.05 | 188 | 5.90 | 5-METHOXY AMT 1 | 56 | 15 | 24 |
| 205.05 | 147.1 | 5.90 | 5-METHOXY AMT 2 | 56 | 27 | 20 |
| 275.078 | 114.2 | 7.16 | 5-METHOXY DiPT 1 | 91 | 21 | 14 |
| 275.078 | 174.1 | 7.16 | 5-METHOXY DiPT 2 | 91 | 29 | 22 |
| 219.968 | 58.1 | 5.16 | 5-METHOXY DMT 1 | 146 | 21 | 8 |
| 219.968 | 175 | 5.16 | 5-METHOXY DMT 2 | 146 | 21 | 22 |
| 238.277 | 190.1 | 5.53 | 5-METHOXY METHYLONE 1 | 96 | 21 | 24 |
| 238.277 | 147.1 | 5.53 | 5-METHOXY METHYLONE 2 | 96 | 37 | 16 |
| 247.988 | 86.1 | 6.16 | 5-METHOXY MiPT 1 | 156 | 19 | 10 |
| 247.988 | 87.1 | 6.16 | 5-METHOXY MiPT 2 | 156 | 19 | 10 |
| 176.049 | 159.1 | 6.45 | 6-APB 1 | 66 | 13 | 20 |
| 176.049 | 131.1 | 6.45 | 6-APB 2 | 66 | 25 | 18 |
| 178.157 | 161 | 5.80 | 6-APDB 1 | 36 | 15 | 22 |
| 178.157 | 132.9 | 5.80 | 6-APDB 2 | 36 | 29 | 8 |
| 272.17 | 240.9 | 7.47 | 6-BROMO-MDMA 1 | 86 | 19 | 12 |
| 274.156 | 242.8 | 7.47 | 6-BROMO-MDMA 2 | 76 | 19 | 12 |
| 228.238 | 169 | 7.09 | 6-CHLORO-MDMA 1 | 61 | 29 | 12 |
| 228.238 | 77 | 7.09 | 6-CHLORO-MDMA 2 | 61 | 53 | 12 |
| 204.15 | 131.1 | 7.10 | 6-EAPB 1 | 56 | 29 | 16 |
| 204.15 | 159.1 | 7.10 | 6-EAPB 2 | 56 | 17 | 8 |
| 175.258 | 158.1 | 5.85 | 6-IT 1 | 51 | 15 | 8 |
| 175.258 | 130.1 | 5.85 | 6-IT 2 | 51 | 31 | 18 |
| 328.01 | 165 | 5.06 | 6-MAM 1 | 141 | 51 | 20 |
| 328.01 | 43 | 5.06 | 6-MAM 2 | 141 | 95 | 8 |
| 190.239 | 131.1 | 6.60 | 6-MAPB 1 | 71 | 27 | 10 |
| 190.239 | 91 | 6.60 | 6-MAPB 2 | 71 | 43 | 10 |
| 286.241 | 121.1 | 6.51 | 7-AMINOCLONAZEPAM 1 | 111 | 39 | 14 |
| 286.241 | 222.1 | 6.51 | 7-AMINOCLONAZEPAM 2 | 111 | 33 | 26 |
| 270.396 | 121.1 | 5.51 | 7-AMINODESMETHYLFLUNITRAZEPAM 1 | 146 | 37 | 14 |
| 270.396 | 77 | 5.51 | 7-AMINODESMETHYLFLUNITRAZEPAM 2 | 146 | 75 | 10 |
| 283.767 | 135.1 | 7.55 | 7-AMINOFLUNITRAZEPAM 1 | 121 | 37 | 18 |
| 283.767 | 77.1 | 7.55 | 7-AMINOFLUNITRAZEPAM 2 | 121 | 87 | 12 |
| 252.005 | 121.1 | 4.57 | 7-AMINONITRAZEPAM 1 | 111 | 35 | 16 |
| 252.005 | 77.1 | 4.57 | 7-AMINONITRAZEPAM 2 | 111 | 73 | 10 |
| 176.239 | 131.1 | 6.68 | 7-APB 1 | 46 | 25 | 14 |
| 176.239 | 77 | 6.68 | 7-APB 2 | 46 | 53 | 10 |
| 178.233 | 161.1 | 6.38 | 7-APDB 1 | 61 | 15 | 10 |
| 178.233 | 133.1 | 6.38 | 7-APDB 2 | 61 | 25 | 18 |
| 355.053 | 125.1 | 11.59 | A-796260 1 | 106 | 27 | 16 |
| 355.053 | 114.1 | 11.59 | A-796260 2 | 106 | 35 | 14 |
| 340.308 | 125.1 | 14.21 | A-834735 1 | 156 | 29 | 16 |
| 340.308 | 55 | 14.21 | A-834735 2 | 156 | 61 | 10 |
| 311.15 | 187.1 | 12.39 | A-836339 1 | 101 | 23 | 12 |
| 311.15 | 125.2 | 12.39 | A-836339 2 | 101 | 33 | 10 |
| 350.211 | 135.1 | 16.10 | AB-001 1 | 146 | 39 | 16 |
| 350.211 | 77.1 | 16.10 | AB-001 2 | 146 | 117 | 12 |
| 352.92 | 98.2 | 11.39 | AB005 1 | 116 | 53 | 12 |
| 352.92 | 112.1 | 11.39 | AB005 2 | 116 | 29 | 16 |
| 357.16 | 241.1 | 14.11 | AB-CHMINACA 1 | 91 | 37 | 12 |
| 357.16 | 312.1 | 14.11 | AB-CHMINACA 2 | 91 | 23 | 14 |
| 369.13 | 352 | 12.67 | AB-FUBINACA 1 | 81 | 13 | 14 |
| 369.13 | 109.1 | 12.67 | AB-FUBINACA 2 | 81 | 47 | 14 |
| 369.269 | 253 | 12.86 | AB-FUBINACA 2-FLUOROBENZYL ISOMER 1 | 86 | 33 | 12 |
| 369.269 | 324.2 | 12.86 | AB-FUBINACA 2-FLUOROBENZYL ISOMER 2 | 86 | 19 | 14 |
| 369.241 | 253 | 12.67 | AB-FUBINACA 3-FLUOROBENZYL ISOMER 1 | 66 | 35 | 12 |
| 369.241 | 324.1 | 12.67 | AB-FUBINACA 3-FLUOROBENZYL ISOMER 2 | 66 | 21 | 14 |
| 331.065 | 215.1 | 13.49 | AB-PINACA 1 | 86 | 33 | 10 |
| 331.065 | 286.1 | 13.49 | AB-PINACA 2 | 86 | 21 | 14 |
| 382.972 | 253 | 13.27 | ADB-FUBINACA 1 | 101 | 33 | 12 |
| 382.972 | 109.1 | 13.27 | ADB-FUBINACA 2 | 101 | 55 | 12 |
| 345.052 | 215 | 14.01 | ADB-PINACA 1 | 91 | 35 | 10 |
| 345.052 | 328.2 | 14.01 | ADB-PINACA 2 | 91 | 13 | 14 |
| 345.335 | 215.1 | 13.69 | ADB-PINACA ISOMER_1 1 | 86 | 33 | 10 |
| 345.335 | 300.2 | 13.69 | ADB-PINACA ISOMER_1 2 | 86 | 21 | 14 |
| 345.304 | 215.1 | 14.06 | ADB-PINACA ISOMER_2 1 | 71 | 35 | 10 |
| 345.304 | 300.1 | 14.06 | ADB-PINACA ISOMER_2 2 | 71 | 19 | 14 |
| 345.292 | 215.1 | 14.05 | ADB-PINACA ISOMER_3 1 | 71 | 33 | 10 |
| 345.292 | 300.2 | 14.05 | ADB-PINACA ISOMER_3 2 | 71 | 19 | 14 |
| 345.305 | 215.1 | 13.98 | ADB-PINACA ISOMER_4 1 | 81 | 33 | 10 |
| 345.305 | 300.1 | 13.98 | ADB-PINACA ISOMER_4 2 | 81 | 19 | 14 |
| 329.174 | 284 | 9.70 | AH-7921 1 | 91 | 23 | 12 |
| 329.174 | 173 | 9.70 | AH-7921 2 | 91 | 35 | 18 |
| 404.265 | 135.1 | 15.64 | AKB48_N-(4-FLUOROBENZYL)_ANALOG 1 | 136 | 29 | 16 |
| 404.265 | 77 | 15.64 | AKB48_N-(4-FLUOROBENZYL)_ANALOG 2 | 136 | 121 | 14 |
| 206.323 | 188.1 | 7.09 | ALFA-ETHYLAMINOPENTIOPHENONE 1 | 86 | 17 | 22 |
| 206.323 | 91 | 7.09 | ALFA-ETHYLAMINOPENTIOPHENONE 2 | 86 | 39 | 12 |
| 220.315 | 202.1 | 7.83 | ALFA-PROPYLAMINOPENTIOPHENONE 1 | 91 | 19 | 10 |
| 220.315 | 91.1 | 7.83 | ALFA-PROPYLAMINOPENTIOPHENONE 2 | 91 | 33 | 10 |
| 238.321 | 221.1 | 6.95 | ALLYLESCALINE 1 | 51 | 13 | 10 |
| 238.321 | 77 | 6.95 | ALLYLESCALINE 2 | 51 | 67 | 12 |
| 206.291 | 91.1 | 6.84 | ALPHA-DIMETHYLAMINOPENTIOPHENONE 1 | 101 | 27 | 14 |
| 206.291 | 77.1 | 6.84 | ALPHA-DIMETHYLAMINOPENTIOPHENONE 2 | 101 | 57 | 12 |
| 220.312 | 91 | 8.41 | ALPHA-ETHYLAMINOHEXANOPHENONE 1 | 106 | 33 | 12 |
| 220.312 | 130.1 | 8.41 | ALPHA-ETHYLAMINOHEXANOPHENONE 2 | 106 | 47 | 14 |
| 188.767 | 130.1 | 7.09 | ALPHA-ETHYLTRYPTAMINE 1 | 76 | 27 | 16 |
| 188.767 | 172.1 | 7.09 | ALPHA-ETHYLTRYPTAMINE 2 | 76 | 11 | 20 |
| 175.029 | 158.1 | 5.68 | ALPHA-METHYLTRYPTAMINE 1 | 56 | 15 | 20 |
| 175.029 | 143.1 | 5.68 | ALPHA-METHYLTRYPTAMINE 2 | 56 | 35 | 16 |
| 246.318 | 91 | 8.41 | ALPHA-PHP 1 | 76 | 33 | 10 |
| 246.318 | 77 | 8.41 | ALPHA-PHP 2 | 76 | 65 | 10 |
| 280.193 | 105.1 | 11.76 | ALPHA-PHTALIMIDOPROPIOPHENONE 1 | 116 | 31 | 14 |
| 280.193 | 77 | 11.76 | ALPHA-PHTALIMIDOPROPIOPHENONE 2 | 116 | 79 | 12 |
| 232.294 | 91.1 | 6.48 | ALPHA-PIPBP 1 | 106 | 33 | 8 |
| 232.294 | 77 | 6.48 | ALPHA-PIPBP 2 | 106 | 61 | 10 |
| 204.261 | 105.1 | 5.00 | ALPHA-PPP 1 | 71 | 31 | 14 |
| 204.261 | 98.1 | 5.00 | ALPHA-PPP 2 | 71 | 33 | 8 |
| 232.243 | 91.1 | 7.17 | ALPHA-PVP 1 | 71 | 39 | 8 |
| 232.243 | 126.1 | 7.17 | ALPHA-PVP 2 | 71 | 57 | 6 |
| 238.017 | 126.2 | 6.24 | ALPHA-PVT 1 | 91 | 29 | 16 |
| 238.017 | 97 | 6.24 | ALPHA-PVT 2 | 91 | 31 | 12 |
| 224.19 | 112.2 | 4.95 | ALPHA-PYRROLIDINOBUTHIOPHENONE 1 | 81 | 29 | 12 |
| 224.19 | 97 | 4.95 | ALPHA-PYRROLIDINOBUTHIOPHENONE 2 | 81 | 39 | 12 |
| 309.934 | 282 | 11.35 | ALPRAZOLAM 1 | 171 | 37 | 12 |
| 309.934 | 206 | 11.35 | ALPRAZOLAM 2 | 171 | 57 | 26 |
| 383.315 | 98 | 10.51 | AM1220 1 | 101 | 49 | 14 |
| 383.315 | 112.1 | 10.51 | AM1220 2 | 101 | 27 | 12 |
| 391.166 | 135.2 | 12.07 | AM-1248 1 | 131 | 39 | 16 |
| 391.166 | 112.1 | 12.07 | AM-1248 2 | 131 | 39 | 14 |
| 360.031 | 127.1 | 14.16 | AM-2201 1 | 171 | 65 | 16 |
| 360.031 | 155.1 | 14.16 | AM-2201 2 | 171 | 33 | 18 |
| 376.237 | 232.1 | 14.94 | AM2201 8-QUINOLINYL CARBOXAMIDE 1 | 86 | 23 | 12 |
| 376.237 | 144.1 | 14.94 | AM2201 8-QUINOLINYL CARBOXAMIDE 2 | 86 | 55 | 16 |
| 361.184 | 127.1 | 14.44 | AM2201 BENZIMIDAZOLE ANALOG 1 | 131 | 73 | 16 |
| 361.184 | 155.1 | 14.44 | AM2201 BENZIMIDAZOLE ANALOG 2 | 131 | 41 | 20 |
| 353.245 | 155.1 | 12.76 | AM2232 1 | 131 | 31 | 18 |
| 353.245 | 127 | 12.76 | AM2232 2 | 131 | 63 | 14 |
| 278.073 | 91 | 10.85 | AMITRIPTYLINE 1 | 101 | 33 | 12 |
| 278.073 | 105.1 | 10.85 | AMITRIPTYLINE 2 | 101 | 31 | 14 |
| 136.03 | 91 | 4.73 | AMPHETAMINE 1 | 51 | 23 | 12 |
| 136.03 | 119.1 | 4.73 | AMPHETAMINE 2 | 51 | 11 | 16 |
| 267.122 | 145.1 | 3.91 | ATENOLOL 1 | 101 | 35 | 20 |
| 267.122 | 56.1 | 3.91 | ATENOLOL 2 | 101 | 43 | 8 |
| 216.245 | 174 | 11.12 | ATRAZYNA 1 | 126 | 23 | 24 |
| 216.245 | 104 | 11.12 | ATRAZYNA 2 | 126 | 39 | 14 |
| 268.279 | 250.1 | 8.69 | AZACYCLONOL 1 | 81 | 17 | 20 |
| 268.279 | 91 | 8.69 | AZACYCLONOL 2 | 81 | 51 | 14 |
| 385.123 | 240.1 | 15.13 | BB-22 1 | 101 | 19 | 16 |
| 385.123 | 144 | 15.13 | BB-22 2 | 101 | 51 | 18 |
| 194.03 | 135 | 6.36 | BDB 1 | 56 | 25 | 18 |
| 194.03 | 177 | 6.36 | BDB 2 | 56 | 11 | 24 |
| 300.293 | 215.1 | 10.62 | BENOCYCLIDINE 1 | 66 | 17 | 10 |
| 300.293 | 147 | 10.62 | BENOCYCLIDINE 2 | 66 | 39 | 18 |
| 254.273 | 91.1 | 9.14 | BENZEDRONE 1 | 111 | 31 | 12 |
| 254.273 | 65.1 | 9.14 | BENZEDRONE 2 | 111 | 77 | 10 |
| 166.03 | 120 | 8.84 | BENZOCAINE 1 | 71 | 25 | 16 |
| 166.03 | 94 | 8.84 | BENZOCAINE 2 | 71 | 23 | 12 |
| 290.034 | 168.1 | 6.54 | BENZOYLECGONINE 1 | 91 | 27 | 20 |
| 290.034 | 77 | 6.54 | BENZOYLECGONINE 2 | 91 | 77 | 10 |
| 310.213 | 86.1 | 10.20 | BENZYDAMINE 1 | 106 | 23 | 10 |
| 310.213 | 58.1 | 10.20 | BENZYDAMINE 2 | 106 | 71 | 10 |
| 274.896 | 163 | 6.96 | bk-2C-B 1 | 81 | 39 | 39 |
| 274.896 | 178.1 | 6.96 | bk-2C-B 2 | 81 | 21 | 21 |
| 194.195 | 146 | 4.23 | bk-MDA 1 | 56 | 19 | 14 |
| 194.195 | 118.1 | 4.23 | bk-MDA 2 | 56 | 31 | 14 |
| 136.133 | 91 | 4.86 | BMPEA 1 | 46 | 25 | 10 |
| 136.133 | 119.1 | 4.86 | BMPEA 2 | 46 | 11 | 16 |
| 293.977 | 276.9 | 10.04 | BROMO-DRAGON-FLY 1 | 71 | 15 | 42 |
| 296.128 | 278.9 | 10.04 | BROMO-DRAGON-FLY 2 | 81 | 17 | 12 |
| 468.189 | 55 | 9.63 | BUPRENORPHINE 1 | 116 | 95 | 12 |
| 468.189 | 152 | 9.63 | BUPRENORPHINE 2 | 116 | 117 | 10 |
| 239.672 | 184 | 8.02 | BUPROPION 1 | 66 | 17 | 24 |
| 239.672 | 131.1 | 8.02 | BUPROPION 2 | 66 | 37 | 16 |
| 222.185 | 174.1 | 5.77 | BUTYLONE (bk-MBDB) 1 | 66 | 25 | 20 |
| 222.185 | 204.1 | 5.77 | BUTYLONE (bk-MBDB) 2 | 66 | 17 | 10 |
| 202.289 | 91 | 8.22 | CAMFETAMINE 1 | 76 | 43 | 14 |
| 202.289 | 67 | 8.22 | CAMFETAMINE 2 | 76 | 25 | 14 |
| 315.323 | 193 | 15.06 | CANNABIDIOL 1 | 121 | 29 | 10 |
| 315.323 | 123 | 15.06 | CANNABIDIOL 2 | 121 | 43 | 16 |
| 237.209 | 194.1 | 10.49 | CARBAMAZEPINE 1 | 111 | 27 | 24 |
| 237.209 | 192.1 | 10.49 | CARBAMAZEPINE 2 | 111 | 45 | 24 |
| 152.256 | 134.1 | 3.67 | CATHINE 1 | 51 | 13 | 22 |
| 152.256 | 117.1 | 3.67 | CATHINE 2 | 51 | 23 | 14 |
| 150.03 | 132.1 | 3.71 | CATHINONE 1 | 56 | 17 | 18 |
| 150.03 | 117.1 | 3.71 | CATHINONE 2 | 56 | 31 | 14 |
| 369.1 | 127 | 16.51 | CB-13 1 | 151 | 61 | 20 |
| 369.1 | 170.7 | 16.51 | CB-13 2 | 151 | 39 | 24 |
| 358.214 | 214.1 | 15.81 | CBL-018 1 | 76 | 21 | 10 |
| 358.214 | 144.1 | 15.81 | CBL-018 2 | 76 | 49 | 20 |
| 394.203 | 188.9 | 14.95 | CI2201 1 | 151 | 37 | 24 |
| 396.214 | 191 | 14.95 | CI2201 2 | 151 | 35 | 24 |
| 301.995 | 260 | 11.09 | CLOBAZAM 1 | 151 | 29 | 12 |
| 301.995 | 225 | 11.09 | CLOBAZAM 2 | 151 | 45 | 26 |
| 315.043 | 86.1 | 11.59 | CLOMIPRAMINE 1 | 106 | 23 | 12 |
| 315.043 | 58 | 11.59 | CLOMIPRAMINE 2 | 106 | 65 | 8 |
| 316.097 | 270.1 | 10.73 | CLONAZEPAM 1 | 151 | 35 | 12 |
| 316.097 | 214.3 | 10.73 | CLONAZEPAM 2 | 151 | 45 | 22 |
| 318.094 | 196.1 | 8.04 | COCAETHYLENE 1 | 101 | 27 | 24 |
| 318.094 | 82 | 8.04 | COCAETHYLENE 2 | 101 | 39 | 12 |
| 304.198 | 182.1 | 7.06 | COCAINE 1 | 106 | 27 | 22 |
| 304.198 | 77.1 | 7.06 | COCAINE 2 | 106 | 79 | 12 |
| 300.058 | 152 | 4.32 | CODEINE 1 | 136 | 85 | 18 |
| 300.058 | 115.1 | 4.32 | CODEINE 2 | 136 | 95 | 16 |
| 349.217 | 231.1 | 14.69 | CUMYL-PICA 1 | 86 | 21 | 12 |
| 349.217 | 91 | 14.69 | CUMYL-PICA 2 | 86 | 73 | 12 |
| 254.299 | 236.2 | 7.95 | D2PM 1 | 81 | 19 | 12 |
| 254.299 | 130.1 | 7.95 | D2PM 2 | 81 | 39 | 8 |
| 286.956 | 179.9 | 10.20 | DEMOXEPAM 1 | 131 | 31 | 22 |
| 286.956 | 269 | 10.20 | DEMOXEPAM 2 | 131 | 37 | 12 |
| 289.917 | 140 | 11.56 | DESALKYLFLURAZEPAM 1 | 116 | 39 | 18 |
| 289.917 | 141 | 11.56 | DESALKYLFLURAZEPAM 2 | 116 | 39 | 18 |
| 310.255 | 281 | 11.39 | DESCHLOROETIZOLAM 1 | 151 | 33 | 12 |
| 310.255 | 256 | 11.39 | DESCHLOROETIZOLAM 2 | 151 | 31 | 12 |
| 218.287 | 91 | 6.21 | DESCHLORO-N-ETHYL-KETAMINE 1 | 96 | 39 | 10 |
| 218.287 | 145.1 | 6.21 | DESCHLORO-N-ETHYL-KETAMINE 2 | 96 | 23 | 8 |
| 267.085 | 72.1 | 10.70 | DESIPRAMINE 1 | 71 | 21 | 10 |
| 267.085 | 44.1 | 10.70 | DESIPRAMINE 2 | 71 | 63 | 8 |
| 271.978 | 209.1 | 11.89 | DESMETHYLDIAZEPAM (NORDIAZEPAM) 2 | 141 | 39 | 26 |
| 271.978 | 140 | 11.89 | DESMETHYLDIAZEPAM (NORDIAZEPAM) 1 | 141 | 39 | 18 |
| 299.977 | 254.1 | 10.31 | DESMETHYLFLUNITRAZEPAM 1 | 116 | 35 | 12 |
| 299.977 | 198 | 10.31 | DESMETHYLFLUNITRAZEPAM 2 | 116 | 53 | 24 |
| 272.254 | 167.1 | 5.08 | DESOMORPHINE 1 | 126 | 49 | 20 |
| 272.254 | 152.1 | 5.08 | DESOMORPHINE 2 | 126 | 73 | 18 |
| 238.301 | 91 | 8.68 | DESOXY-D2PM 1 | 101 | 33 | 12 |
| 238.301 | 117.1 | 8.68 | DESOXY-D2PM 2 | 101 | 23 | 8 |
| 252.331 | 91.1 | 9.03 | DESOXYPIPRADROL (2-DPMP) 1 | 91 | 49 | 12 |
| 252.331 | 65 | 9.03 | DESOXYPIPRADROL (2-DPMP) 2 | 91 | 85 | 10 |
| 272.289 | 128.1 | 9.23 | DEXTROMETHORPHAN 1 | 126 | 81 | 16 |
| 272.289 | 171 | 9.23 | DEXTROMETHORPHAN 2 | 126 | 51 | 20 |
| 285.397 | 154 | 12.30 | DIAZEPAM 1 | 166 | 37 | 20 |
| 285.397 | 193 | 12.30 | DIAZEPAM 2 | 166 | 43 | 24 |
| 320.306 | 228 | 12.33 | DICLAZEPAM 1 | 166 | 43 | 28 |
| 320.306 | 89 | 12.33 | DICLAZEPAM 2 | 166 | 103 | 12 |
| 322.115 | 121.1 | 11.03 | DICLOFENSINE 1 | 151 | 33 | 16 |
| 322.115 | 279 | 11.03 | DICLOFENSINE 2 | 151 | 29 | 14 |
| 206.061 | 105.1 | 5.24 | DIETHYLCATHINONE (AMFEPRAMONE) 1 | 81 | 31 | 12 |
| 206.061 | 100.1 | 5.24 | DIETHYLCATHINONE (AMFEPRAMONE) 2 | 81 | 29 | 14 |
| 302.062 | 199 | 4.26 | DIHYDROCODEINE 1 | 121 | 43 | 26 |
| 302.062 | 128.1 | 4.26 | DIHYDROCODEINE 2 | 121 | 81 | 16 |
| 279.175 | 120.1 | 6.17 | DIMETHOCAINE 1 | 106 | 31 | 14 |
| 279.175 | 65 | 6.17 | DIMETHOCAINE 2 | 106 | 81 | 10 |
| 222.209 | 72.2 | 4.77 | DIMETHYLONE (bk-MDDMA) 1 | 86 | 25 | 10 |
| 222.209 | 91 | 4.77 | DIMETHYLONE (bk-MDDMA) 2 | 86 | 49 | 12 |
| 256.071 | 167.1 | 9.27 | DIPHENHYDRAMINE 1 | 56 | 17 | 22 |
| 256.071 | 165.1 | 9.27 | DIPHENHYDRAMINE 2 | 56 | 57 | 22 |
| 266.099 | 181.1 | 8.71 | DIPHENIDINE 1 | 76 | 25 | 22 |
| 266.099 | 103.1 | 8.71 | DIPHENIDINE 2 | 76 | 47 | 12 |
| 244.891 | 114.2 | 7.30 | DiPT 1 | 81 | 21 | 14 |
| 244.891 | 144.1 | 7.30 | DiPT 2 | 81 | 31 | 20 |
| 266.292 | 248.1 | 7.33 | DL-4662 1 | 86 | 19 | 12 |
| 266.292 | 188 | 7.33 | DL-4662 2 | 86 | 35 | 22 |
| 322.068 | 304.9 | 8.92 | DOI 1 | 61 | 17 | 12 |
| 322.068 | 277 | 8.92 | DOI 2 | 61 | 27 | 12 |
| 296.089 | 202 | 10.16 | DOTHIEPIN 1 | 86 | 75 | 26 |
| 296.089 | 220.9 | 10.16 | DOTHIEPIN 2 | 86 | 61 | 28 |
| 279.918 | 107.1 | 9.52 | DOXEPIN 1 | 101 | 31 | 14 |
| 279.918 | 77 | 9.52 | DOXEPIN 2 | 101 | 73 | 10 |
| 271.067 | 167.1 | 5.77 | DOXYLAMINE 1 | 61 | 49 | 22 |
| 271.067 | 182 | 5.77 | DOXYLAMINE 2 | 61 | 23 | 22 |
| 388.297 | 183.1 | 14.95 | EAM-2201 1 | 81 | 35 | 10 |
| 388.297 | 153 | 14.95 | EAM-2201 2 | 81 | 65 | 20 |
| 278.507 | 234.2 | 9.25 | EDDP 1 | 86 | 41 | 10 |
| 278.507 | 249.1 | 9.25 | EDDP 2 | 86 | 33 | 10 |
| 410.225 | 155 | 15.66 | EG-2201 1 | 181 | 33 | 22 |
| 410.225 | 127.1 | 15.66 | EG-2201 2 | 181 | 71 | 16 |
| 326.4 | 223.1 | 5.32 | ERGOMETRINE 1 | 116 | 31 | 10 |
| 326.4 | 208.1 | 5.32 | ERGOMETRINE 2 | 116 | 39 | 10 |
| 295.921 | 268 | 10.99 | ESTAZOLAM 1 | 101 | 33 | 12 |
| 295.921 | 206 | 10.99 | ESTAZOLAM 2 | 101 | 55 | 24 |
| 264.918 | 146.1 | 11.91 | ETAQUALONE 1 | 116 | 37 | 18 |
| 264.918 | 77 | 11.91 | ETAQUALONE 2 | 116 | 89 | 12 |
| 180.064 | 162.1 | 4.72 | ETHCATHINONE METABOLITE 1 | 76 | 17 | 20 |
| 180.064 | 115.1 | 4.72 | ETHCATHINONE METABOLITE 2 | 76 | 39 | 14 |
| 222.228 | 174.1 | 5.14 | ETHYLONE (bk-MDEA) 1 | 71 | 25 | 20 |
| 222.228 | 204.1 | 5.14 | ETHYLONE (bk-MDEA) 2 | 71 | 19 | 10 |
| 248.328 | 84.1 | 8.17 | ETHYLPHENIDATE 1 | 111 | 25 | 12 |
| 248.328 | 56 | 8.17 | ETHYLPHENIDATE 2 | 111 | 69 | 8 |
| 343.91 | 315 | 11.69 | ETIZOLAM 1 | 121 | 35 | 14 |
| 343.91 | 224.1 | 11.69 | ETIZOLAM 2 | 121 | 65 | 10 |
| 236.294 | 188.1 | 6.18 | EUTYLONE (bk-EBDB) 1 | 96 | 25 | 24 |
| 236.294 | 189.1 | 6.18 | EUTYLONE (bk-EBDB) 2 | 96 | 29 | 22 |
| 396.194 | 109.1 | 15.21 | FDU-PB-22 1 | 96 | 47 | 12 |
| 396.194 | 252 | 15.21 | FDU-PB-22 2 | 96 | 17 | 12 |
| 232.018 | 159 | 8.34 | FENFLURAMINE 1 | 86 | 31 | 20 |
| 232.018 | 109 | 8.34 | FENFLURAMINE 2 | 86 | 57 | 14 |
| 337.171 | 188 | 8.81 | FENTANYL 1 | 131 | 29 | 10 |
| 337.171 | 105 | 8.81 | FENTANYL 2 | 131 | 57 | 12 |
| 334.716 | 226.1 | 11.77 | FLUBROMAZEPAM 1 | 161 | 39 | 10 |
| 334.716 | 186 | 11.77 | FLUBROMAZEPAM 2 | 161 | 41 | 22 |
| 303.922 | 212 | 11.99 | FLUDIAZEPAM 1 | 141 | 43 | 26 |
| 303.922 | 89 | 11.99 | FLUDIAZEPAM 2 | 141 | 93 | 12 |
| 303.986 | 258.1 | 9.20 | FLUMAZENIL 1 | 81 | 23 | 12 |
| 303.986 | 217 | 9.20 | FLUMAZENIL 2 | 81 | 35 | 26 |
| 313.814 | 268 | 10.83 | FLUNITRAZEPAM 1 | 136 | 35 | 12 |
| 313.814 | 239 | 10.83 | FLUNITRAZEPAM 2 | 136 | 47 | 12 |
| 310.021 | 44.1 | 11.20 | FLUOXETINE 1 | 51 | 43 | 8 |
| 310.021 | 148.1 | 11.20 | FLUOXETINE 2 | 51 | 11 | 18 |
| 388.023 | 315 | 9.32 | FLURAZEPAM 1 | 91 | 31 | 14 |
| 388.023 | 107 | 9.32 | FLURAZEPAM 2 | 91 | 111 | 14 |
| 350.227 | 109.1 | 14.95 | FUB-144 1 | 111 | 61 | 14 |
| 350.227 | 125.1 | 14.95 | FUB-144 2 | 111 | 29 | 14 |
| 380.203 | 155.1 | 14.47 | FUB-JWH-018 1 | 176 | 33 | 8 |
| 380.203 | 109 | 14.47 | FUB-JWH-018 2 | 176 | 63 | 18 |
| 398.186 | 253 | 13.65 | FUB-NPB-22 1 | 86 | 23 | 12 |
| 398.186 | 109.1 | 13.65 | FUB-NPB-22 2 | 86 | 47 | 8 |
| 396.975 | 109.1 | 15.21 | FUB-PB-22 1 | 116 | 45 | 14 |
| 396.975 | 253.1 | 15.21 | FUB-PB-22 2 | 116 | 17 | 12 |
| 375.29 | 105.1 | 8.86 | FURANYLFENTANYL (Fu-F) 1 | 136 | 55 | 14 |
| 375.29 | 188.1 | 8.86 | FURANYLFENTANYL (Fu-F) 2 | 136 | 29 | 20 |
| 102.935 | 85 | 1.31 | GHB 1 | -75 | -10 | -12 |
| 102.935 | 57 | 1.31 | GHB 2 | -75 | -10 | -18 |
| 215.051 | 174 | 7.45 | HARMALINE 1 | 61 | 29 | 10 |
| 215.051 | 171.9 | 7.45 | HARMALINE 2 | 61 | 37 | 18 |
| 212.795 | 170.1 | 7.85 | HARMINE 1 | 121 | 41 | 20 |
| 212.795 | 198 | 7.85 | HARMINE 2 | 121 | 31 | 26 |
| 387.195 | 243.1 | 15.91 | HU-210 1 | 146 | 25 | 12 |
| 387.195 | 43 | 15.91 | HU-210 2 | 146 | 71 | 8 |
| 300.05 | 199 | 4.83 | HYDROCODONE 1 | 131 | 39 | 24 |
| 300.05 | 128.1 | 4.83 | HYDROCODONE 2 | 131 | 77 | 16 |
| 312.288 | 122.1 | 7.91 | IBOGAINE 1 | 281 | 43 | 14 |
| 312.288 | 77 | 7.91 | IBOGAINE 2 | 281 | 119 | 12 |
| 281.078 | 86.1 | 10.55 | IMIPRAMINE 1 | 66 | 21 | 12 |
| 281.078 | 58.1 | 10.55 | IMIPRAMINE 2 | 66 | 59 | 8 |
| 192.055 | 91 | 6.81 | ISOPENTEDRONE 1 | 71 | 33 | 12 |
| 192.055 | 161 | 6.81 | ISOPENTEDRONE 2 | 71 | 17 | 22 |
| 343.221 | 126.6 | 15.44 | JWH 018 BENZIMIDAZOLE ANALOG 1 | 121 | 109 | 16 |
| 343.221 | 155.1 | 15.44 | JWH 018 BENZIMIDAZOLE ANALOG 2 | 121 | 41 | 20 |
| 384.202 | 155.1 | 15.80 | JWH-011 1 | 151 | 37 | 16 |
| 384.202 | 127.1 | 15.80 | JWH-011 2 | 151 | 71 | 14 |
| 342.097 | 127.1 | 15.16 | JWH-016 1 | 146 | 67 | 16 |
| 342.097 | 155.1 | 15.16 | JWH-016 2 | 146 | 33 | 20 |
| 342.063 | 127.1 | 14.91 | JWH-018 1 | 146 | 65 | 16 |
| 342.063 | 155 | 14.91 | JWH-018 2 | 146 | 33 | 20 |
| 365.291 | 135.1 | 15.72 | JWH-018 ADAMANTYL CARBOXAMIDE 1 | 156 | 37 | 20 |
| 365.291 | 77 | 15.72 | JWH-018 ADAMANTYL CARBOXAMIDE 2 | 156 | 109 | 12 |
| 370.197 | 127.1 | 15.90 | JWH-020 1 | 156 | 67 | 16 |
| 370.197 | 155 | 15.90 | JWH-020 2 | 156 | 35 | 20 |
| 340.223 | 155.1 | 14.75 | JWH-022 1 | 136 | 31 | 8 |
| 340.223 | 127.1 | 14.75 | JWH-022 2 | 136 | 61 | 16 |
| 306.155 | 127.1 | 14.85 | JWH-031 1 | 131 | 51 | 8 |
| 306.155 | 76.9 | 14.85 | JWH-031 2 | 131 | 95 | 6 |
| 300.22 | 127.1 | 13.63 | JWH-071 1 | 111 | 57 | 16 |
| 300.22 | 155.1 | 13.63 | JWH-071 2 | 111 | 29 | 8 |
| 328.077 | 127.1 | 14.71 | JWH-073 1 | 131 | 63 | 16 |
| 328.077 | 155.1 | 14.71 | JWH-073 2 | 131 | 31 | 20 |
| 358.107 | 185 | 14.96 | JWH-080 1 | 61 | 33 | 24 |
| 358.107 | 127.1 | 14.96 | JWH-080 2 | 61 | 69 | 16 |
| 372.132 | 185.1 | 15.37 | JWH-081 1 | 156 | 35 | 24 |
| 372.132 | 114.1 | 15.37 | JWH-081 2 | 156 | 93 | 14 |
| 386.111 | 185 | 15.50 | JWH-098 1 | 146 | 35 | 22 |
| 386.111 | 114.1 | 15.50 | JWH-098 2 | 146 | 101 | 14 |
| 370.219 | 155.2 | 15.65 | JWH-116 1 | 121 | 33 | 16 |
| 370.219 | 127.1 | 15.65 | JWH-116 2 | 121 | 69 | 6 |
| 356.234 | 169.1 | 15.53 | JWH-122 1 | 126 | 33 | 20 |
| 356.234 | 115.1 | 15.53 | JWH-122 2 | 126 | 91 | 14 |
| 367.929 | 154.8 | 15.59 | JWH-145 1 | 91 | 35 | 22 |
| 367.929 | 127.1 | 15.59 | JWH-145 2 | 91 | 69 | 18 |
| 396.312 | 155.1 | 16.20 | JWH-146 1 | 141 | 27 | 18 |
| 396.312 | 127 | 16.20 | JWH-146 2 | 141 | 71 | 22 |
| 382.164 | 155 | 15.91 | JWH-147 1 | 136 | 27 | 20 |
| 382.164 | 127.1 | 15.91 | JWH-147 2 | 136 | 69 | 16 |
| 370.172 | 169.1 | 15.65 | JWH-149 1 | 156 | 35 | 26 |
| 370.172 | 115.2 | 15.65 | JWH-149 2 | 156 | 95 | 14 |
| 306.156 | 91.1 | 14.55 | JWH-167 1 | 86 | 31 | 12 |
| 306.156 | 214.2 | 14.55 | JWH-167 2 | 86 | 35 | 30 |
| 328.281 | 141.1 | 16.76 | JWH-175 1 | 106 | 29 | 18 |
| 328.281 | 115.1 | 16.76 | JWH-175 2 | 106 | 83 | 14 |
| 384.268 | 197 | 16.06 | JWH-182 1 | 196 | 33 | 10 |
| 384.268 | 141 | 16.06 | JWH-182 2 | 196 | 61 | 14 |
| 399.123 | 169.1 | 11.73 | JWH-193 1 | 116 | 31 | 10 |
| 399.123 | 115 | 11.73 | JWH-193 2 | 116 | 97 | 6 |
| 415.117 | 185.1 | 11.51 | JWH-198 1 | 181 | 33 | 22 |
| 415.117 | 114.2 | 11.51 | JWH-198 2 | 181 | 35 | 10 |
| 385.076 | 155.1 | 10.92 | JWH-200 1 | 121 | 29 | 20 |
| 385.076 | 127.1 | 10.92 | JWH-200 2 | 121 | 71 | 16 |
| 336.263 | 121.1 | 14.46 | JWH-201 1 | 151 | 35 | 16 |
| 336.263 | 77 | 14.46 | JWH-201 2 | 151 | 77 | 10 |
| 384.326 | 183.1 | 15.91 | JWH-213 1 | 181 | 35 | 22 |
| 384.326 | 153.1 | 15.91 | JWH-213 2 | 181 | 61 | 18 |
| 386.153 | 155 | 15.47 | JWH-307 1 | 66 | 27 | 24 |
| 386.153 | 127.1 | 15.47 | JWH-307 2 | 66 | 81 | 16 |
| 418.325 | 155 | 16.09 | JWH-309 1 | 141 | 29 | 20 |
| 418.325 | 127.1 | 16.09 | JWH-309 2 | 141 | 77 | 16 |
| 386.132 | 155.1 | 15.59 | JWH-368 1 | 106 | 29 | 20 |
| 386.132 | 127 | 15.59 | JWH-368 2 | 106 | 69 | 16 |
| 402.279 | 155.1 | 15.69 | JWH-369 1 | 126 | 29 | 20 |
| 402.279 | 127 | 15.69 | JWH-369 2 | 126 | 65 | 18 |
| 360.213 | 173 | 15.41 | JWH-412 1 | 161 | 33 | 10 |
| 360.213 | 145.1 | 15.41 | JWH-412 2 | 161 | 63 | 18 |
| 422.179 | 235 | 14.95 | JWH-424 1 | 131 | 37 | 12 |
| 420.174 | 233 | 14.95 | JWH-424 2 | 111 | 37 | 12 |
| 238.083 | 125 | 6.41 | KETAMINE 1 | 61 | 37 | 16 |
| 238.083 | 89 | 6.41 | KETAMINE 2 | 61 | 73 | 12 |
| 324.164 | 223 | 8.19 | LAMPA 1 | 116 | 33 | 28 |
| 324.164 | 208 | 8.19 | LAMPA 2 | 116 | 41 | 26 |
| 235.081 | 86.1 | 5.70 | LIDOCAINE 1 | 126 | 23 | 10 |
| 235.081 | 58.1 | 5.70 | LIDOCAINE 2 | 126 | 51 | 10 |
| 464.983 | 252 | 9.21 | LOPRAZOLAM 1 | 126 | 57 | 12 |
| 464.983 | 111.1 | 9.21 | LOPRAZOLAM 2 | 126 | 35 | 14 |
| 321.924 | 275.9 | 11.33 | LORAZEPAM 1 | 111 | 29 | 12 |
| 321.924 | 303.9 | 11.33 | LORAZEPAM 2 | 111 | 21 | 14 |
| 335.939 | 289.9 | 11.79 | LORMETAZEPAM 1 | 86 | 29 | 14 |
| 335.939 | 177.9 | 11.79 | LORMETAZEPAM 2 | 86 | 45 | 12 |
| 324.277 | 223 | 8.02 | LSD 1 | 101 | 33 | 28 |
| 324.277 | 207 | 8.02 | LSD 2 | 101 | 57 | 26 |
| 374.126 | 169 | 14.60 | MAM-2201 1 | 126 | 35 | 22 |
| 374.126 | 115.1 | 14.60 | MAM-2201 2 | 126 | 97 | 14 |
| 278.114 | 191 | 10.71 | MAPROTILINE 1 | 116 | 47 | 24 |
| 278.114 | 189 | 10.71 | MAPROTILINE 2 | 116 | 83 | 22 |
| 208.04 | 135 | 6.39 | MBDB 1 | 51 | 27 | 18 |
| 208.04 | 77 | 6.39 | MBDB 2 | 51 | 55 | 10 |
| 192.204 | 175.1 | 5.49 | MDAT 1 | 66 | 15 | 10 |
| 192.204 | 117.1 | 5.49 | MDAT 2 | 66 | 35 | 8 |
| 221.272 | 135 | 2.36 | MDBP 1 | 61 | 23 | 10 |
| 221.272 | 77 | 2.36 | MDBP 2 | 61 | 53 | 12 |
| 208.054 | 163 | 5.80 | MDEA 1 | 66 | 19 | 20 |
| 208.054 | 105 | 5.80 | MDEA 2 | 66 | 35 | 14 |
| 385.124 | 240.1 | 15.12 | MDMB-CHMICA (MMB-CHMINACA) 1 | 81 | 27 | 12 |
| 385.124 | 144 | 15.12 | MDMB-CHMICA (MMB-CHMINACA) 2 | 81 | 53 | 18 |
| 386.303 | 241.1 | 15.49 | MDMB-CHMINACA 1 | 156 | 33 | 12 |
| 386.303 | 145 | 15.49 | MDMB-CHMINACA 2 | 156 | 57 | 18 |
| 262.024 | 161 | 6.30 | MDPBP 1 | 86 | 31 | 20 |
| 262.024 | 112.1 | 6.30 | MDPBP 2 | 86 | 35 | 14 |
| 329.969 | 284 | 11.40 | MECLONAZEPAM 1 | 91 | 37 | 14 |
| 329.969 | 238 | 11.40 | MECLONAZEPAM 2 | 91 | 57 | 12 |
| 271.019 | 206.9 | 9.31 | MEDAZEPAM 1 | 126 | 37 | 26 |
| 271.019 | 165.1 | 9.31 | MEDAZEPAM 2 | 126 | 61 | 20 |
| 180.261 | 163.2 | 9.37 | MEMANITINE 1 | 56 | 19 | 8 |
| 180.261 | 107.2 | 9.37 | MEMANITINE 2 | 56 | 33 | 10 |
| 248.14 | 220.1 | 7.50 | MEPERIDINE 1 | 121 | 31 | 20 |
| 248.14 | 91.1 | 7.50 | MEPERIDINE 2 | 121 | 59 | 8 |
| 190.139 | 44 | 5.33 | MEPHTETRAMINE (MTTA) 1 | 101 | 31 | 8 |
| 190.139 | 147.1 | 5.33 | MEPHTETRAMINE (MTTA) 2 | 101 | 13 | 18 |
| 314.241 | 214.1 | 10.12 | MEPIRAPIM 1 | 66 | 21 | 10 |
| 314.241 | 144.1 | 10.12 | MEPIRAPIM 2 | 66 | 47 | 8 |
| 219.029 | 158.1 | 8.76 | MEPROBAMATE 1 | 66 | 11 | 20 |
| 219.029 | 55.1 | 8.76 | MEPROBAMATE 2 | 66 | 31 | 8 |
| 212.183 | 195.1 | 5.11 | MESCALINE 1 | 76 | 13 | 24 |
| 212.183 | 77 | 5.11 | MESCALINE 2 | 76 | 63 | 12 |
| 310.094 | 265.1 | 10.75 | METHADONE 1 | 66 | 21 | 12 |
| 310.094 | 105 | 10.75 | METHADONE 2 | 66 | 35 | 14 |
| 150.063 | 91 | 4.96 | METHAMPHETAMINE 1 | 51 | 25 | 12 |
| 150.063 | 119.1 | 4.96 | METHAMPHETAMINE 2 | 51 | 15 | 16 |
| 301.06 | 121.2 | 12.42 | METHANDIENONE 1 | 131 | 35 | 14 |
| 301.06 | 149.1 | 12.42 | METHANDIENONE 2 | 131 | 21 | 18 |
| 251.007 | 132.1 | 11.03 | METHAQUALONE 1 | 131 | 37 | 18 |
| 251.007 | 91 | 11.03 | METHAQUALONE 2 | 131 | 57 | 12 |
| 164.056 | 131.1 | 4.07 | METHCATHINONE 1 | 61 | 27 | 18 |
| 164.056 | 130.1 | 4.07 | METHCATHINONE 2 | 61 | 41 | 16 |
| 193.998 | 176 | 5.29 | METHEDRONE (bk-PMMA) 1 | 56 | 15 | 10 |
| 193.998 | 161 | 5.29 | METHEDRONE (bk-PMMA) 2 | 56 | 31 | 6 |
| 263.072 | 221 | 11.80 | METHOHEXITAL 1 | 116 | 19 | 10 |
| 263.072 | 77.1 | 11.80 | METHOHEXITAL 2 | 116 | 67 | 10 |
| 248.059 | 121.1 | 7.08 | METHOXETAMINE 1 | 76 | 37 | 16 |
| 248.059 | 203 | 7.08 | METHOXETAMINE 2 | 76 | 19 | 26 |
| 297.324 | 129.1 | 9.25 | METHOXPHENIDINE 1 | 101 | 27 | 16 |
| 297.324 | 117.1 | 9.25 | METHOXPHENIDINE 2 | 101 | 31 | 14 |
| 116.066 | 57 | 5.74 | METHYLHEXANAMINE 1 | 71 | 17 | 8 |
| 116.066 | 41.1 | 5.74 | METHYLHEXANAMINE 2 | 71 | 31 | 6 |
| 208.272 | 160 | 4.56 | METHYLONE (bk-MDMA) 1 | 86 | 25 | 20 |
| 208.272 | 132.1 | 4.56 | METHYLONE (bk-MDMA) 2 | 86 | 37 | 14 |
| 234.3 | 84.1 | 7.11 | METHYLPHENIDATE 1 | 71 | 23 | 10 |
| 234.3 | 56 | 7.11 | METHYLPHENIDATE 2 | 71 | 65 | 8 |
| 208.3 | 91 | 6.34 | MEXEDRONE 1 | 76 | 47 | 16 |
| 208.3 | 119 | 6.34 | MEXEDRONE 2 | 76 | 29 | 6 |
| 265.493 | 208.1 | 9.27 | MIANSERIN 1 | 146 | 29 | 24 |
| 265.493 | 58.1 | 9.27 | MIANSERIN 2 | 146 | 45 | 10 |
| 326.065 | 291.1 | 9.28 | MIDAZOLAM 1 | 136 | 37 | 12 |
| 326.065 | 248.9 | 9.28 | MIDAZOLAM 2 | 136 | 51 | 30 |
| 399.094 | 174 | 9.16 | MITRAGYNINE 1 | 136 | 41 | 22 |
| 399.094 | 159.1 | 9.16 | MITRAGYNINE 2 | 136 | 63 | 20 |
| 178.131 | 161 | 6.85 | MMAI 1 | 61 | 15 | 22 |
| 178.131 | 103 | 6.85 | MMAI 2 | 61 | 53 | 14 |
| 345.251 | 214.1 | 14.15 | MMB018 1 | 66 | 19 | 10 |
| 345.251 | 144 | 14.15 | MMB018 2 | 66 | 51 | 20 |
| 363.251 | 232.1 | 13.10 | MMB2201 1 | 81 | 21 | 10 |
| 363.251 | 144.1 | 13.10 | MMB2201 2 | 81 | 53 | 16 |
| 358.061 | 215.1 | 15.53 | MN-18 1 | 126 | 25 | 10 |
| 358.061 | 145 | 15.53 | MN-18 2 | 126 | 49 | 18 |
| 440.336 | 114.1 | 12.76 | MN-25 1 | 141 | 41 | 12 |
| 440.336 | 261.1 | 12.76 | MN-25 2 | 141 | 33 | 12 |
| 454.347 | 114.1 | 13.18 | MN-25-2-METHYL DERIVATIVE 1 | 151 | 41 | 18 |
| 454.347 | 275.1 | 13.18 | MN-25-2-METHYL DERIVATIVE 2 | 151 | 31 | 14 |
| 387.261 | 241.1 | 15.46 | MO-CHMINACA 1 | 121 | 27 | 22 |
| 387.261 | 145.1 | 15.46 | MO-CHMINACA 2 | 121 | 47 | 8 |
| 286.043 | 152.1 | 2.36 | MORPHINE 1 | 176 | 79 | 20 |
| 286.043 | 128.1 | 2.36 | MORPHINE 2 | 176 | 77 | 16 |
| 232.059 | 105.1 | 7.46 | MPBP 1 | 116 | 33 | 14 |
| 232.059 | 91.1 | 7.46 | MPBP 2 | 116 | 57 | 12 |
| 349.38 | 181 | 10.78 | MT-45 1 | 176 | 37 | 24 |
| 349.38 | 77 | 10.78 | MT-45 2 | 176 | 93 | 12 |
| 191.273 | 105.1 | 3.89 | N-(3-METHYLBENZYL)PIPERAZINE 1 | 81 | 27 | 14 |
| 191.273 | 77 | 3.89 | N-(3-METHYLBENZYL)PIPERAZINE 2 | 81 | 55 | 10 |
| 178.273 | 105.1 | 5.31 | N.N-DIETHYLPHENETHYLAMINE 1 | 101 | 25 | 10 |
| 178.273 | 77 | 5.31 | N.N-DIETHYLPHENETHYLAMINE 2 | 101 | 55 | 12 |
| 250.302 | 100.1 | 7.20 | N.N-DIMETHYLPENTYLONE (bk-DMBDP) 1 | 101 | 27 | 12 |
| 250.302 | 135 | 7.20 | N.N-DIMETHYLPENTYLONE (bk-DMBDP) 2 | 101 | 31 | 14 |
| 189.572 | 58 | 4.73 | N.N-DMT 1 | 116 | 19 | 8 |
| 189.572 | 144.1 | 4.73 | N.N-DMT 2 | 116 | 23 | 18 |
| 250.245 | 208.1 | 9.24 | N-ACETYL-3.4-MDMC 1 | 76 | 19 | 22 |
| 250.245 | 160.1 | 9.24 | N-ACETYL-3.4-MDMC 2 | 76 | 35 | 18 |
| 282.065 | 140.9 | 10.07 | NAPHYRONE 1 | 51 | 37 | 10 |
| 282.065 | 127 | 10.07 | NAPHYRONE 2 | 51 | 59 | 24 |
| 282.125 | 127 | 9.59 | NAPHYRONE-1-NAPHTYL ISOMER 1 | 51 | 57 | 22 |
| 282.125 | 141 | 9.59 | NAPHYRONE-1-NAPHTYL ISOMER 2 | 51 | 37 | 10 |
| 298.241 | 91.1 | 8.71 | N-BENZYLNORBUTYLONE 1 | 96 | 41 | 14 |
| 298.241 | 65 | 8.71 | N-BENZYLNORBUTYLONE 2 | 96 | 91 | 16 |
| 194.253 | 121 | 6.10 | N-ETHYL-4-METHOXYAMPHETAMNE 1 | 66 | 29 | 16 |
| 194.253 | 91 | 6.10 | N-ETHYL-4-METHOXYAMPHETAMNE 2 | 66 | 45 | 14 |
| 192.296 | 130.1 | 5.78 | N-ETHYLBUPHEDRONE 1 | 66 | 39 | 10 |
| 192.296 | 91 | 5.78 | N-ETHYLBUPHEDRONE 2 | 66 | 35 | 14 |
| 192.099 | 105 | 4.85 | N-ETHYL-N-METHYLCATHINONE 1 | 76 | 29 | 6 |
| 192.099 | 77.2 | 4.85 | N-ETHYL-N-METHYLCATHINONE 2 | 76 | 57 | 14 |
| 299.364 | 165.1 | 12.84 | N-ETHYLNORDAZEPAM 1 | 216 | 59 | 20 |
| 299.364 | 77 | 12.84 | N-ETHYLNORDAZEPAM 2 | 216 | 89 | 10 |
| 252.18 | 125 | 7.10 | N-ETHYLNORKETAMINE 1 | 71 | 41 | 16 |
| 252.18 | 89.1 | 7.10 | N-ETHYLNORKETAMINE 2 | 71 | 75 | 14 |
| 315.994 | 270 | 12.19 | N-ETHYLOXAZEPAM 1 | 116 | 29 | 12 |
| 315.994 | 242 | 12.19 | N-ETHYLOXAZEPAM 2 | 116 | 47 | 12 |
| 250.269 | 232.2 | 7.42 | N-ETHYLPENTYLONE 1 | 71 | 21 | 22 |
| 250.269 | 202 | 7.42 | N-ETHYLPENTYLONE 2 | 71 | 27 | 12 |
| 296.187 | 250.1 | 10.91 | NIMETAZEPAM 1 | 171 | 35 | 12 |
| 296.187 | 221 | 10.91 | NIMETAZEPAM 2 | 171 | 45 | 26 |
| 309.822 | 92 | 7.76 | NITRACAINE 1 | 216 | 57 | 12 |
| 309.822 | 76 | 7.76 | NITRACAINE 2 | 216 | 91 | 12 |
| 281.974 | 236 | 10.55 | NITRAZEPAM 1 | 141 | 33 | 30 |
| 281.974 | 180.1 | 10.55 | NITRAZEPAM 2 | 141 | 51 | 24 |
| 376.276 | 232 | 14.93 | NM2201 1 | 131 | 21 | 12 |
| 376.276 | 144.1 | 14.93 | NM2201 2 | 131 | 51 | 18 |
| 148.043 | 117.1 | 4.24 | N-METHYL-2AI 1 | 81 | 23 | 14 |
| 148.043 | 115.1 | 4.24 | N-METHYL-2AI 2 | 81 | 37 | 14 |
| 136.182 | 105.1 | 3.68 | N-METHYL-PEA 1 | 51 | 19 | 18 |
| 136.182 | 77 | 3.68 | N-METHYL-PEA 2 | 51 | 43 | 12 |
| 175.508 | 144.1 | 4.73 | N-METHYLTRYPTAMINE 1 | 66 | 17 | 18 |
| 175.508 | 117.1 | 4.73 | N-METHYLTRYPTAMINE 2 | 66 | 37 | 14 |
| 178.233 | 105 | 4.29 | NN-DMC 1 | 91 | 29 | 14 |
| 178.233 | 72.1 | 4.29 | NN-DMC 2 | 91 | 29 | 10 |
| 357.005 | 214.2 | 14.48 | NNEI 1 | 81 | 29 | 10 |
| 357.005 | 144 | 14.48 | NNEI 2 | 81 | 53 | 20 |
| 286.954 | 245.1 | 10.65 | NORCLOBAZAM 1 | 141 | 27 | 12 |
| 286.954 | 210 | 10.65 | NORCLOBAZAM 2 | 141 | 43 | 26 |
| 301.026 | 72.1 | 11.72 | NORCLOMIPRAMINE 1 | 101 | 21 | 10 |
| 301.026 | 44.1 | 11.72 | NORCLOMIPRAMINE 2 | 101 | 65 | 8 |
| 224.176 | 125.1 | 6.37 | NORKETAMINE 1 | 51 | 13 | 12 |
| 224.176 | 207.2 | 6.37 | NORKETAMINE 2 | 51 | 21 | 12 |
| 264.066 | 91.1 | 10.99 | NORTRIPTYLINE 1 | 81 | 29 | 12 |
| 264.066 | 105.1 | 10.99 | NORTRIPTYLINE 2 | 81 | 27 | 14 |
| 178.103 | 119.1 | 6.64 | N-PROPYLAMPHETAMINE 1 | 86 | 17 | 16 |
| 178.103 | 65 | 6.64 | N-PROPYLAMPHETAMINE 2 | 86 | 61 | 10 |
| 242.25 | 181.1 | 9.83 | NRG-3 1 | 81 | 35 | 8 |
| 242.25 | 180.1 | 9.83 | NRG-3 2 | 81 | 53 | 16 |
| 197.229 | 154.1 | 6.76 | o-CPP 1 | 121 | 27 | 20 |
| 197.229 | 118.1 | 6.76 | o-CPP 2 | 121 | 45 | 8 |
| 235.279 | 100.1 | 6.08 | OCTACAINE 1 | 81 | 23 | 14 |
| 235.279 | 72.1 | 6.08 | OCTACAINE 2 | 81 | 51 | 10 |
| 384.309 | 270.2 | 11.55 | ORG-28611 1 | 71 | 25 | 12 |
| 384.309 | 174 | 11.55 | ORG-28611 2 | 71 | 47 | 22 |
| 288.031 | 242.1 | 11.31 | OXAZEPAM 1 | 91 | 31 | 12 |
| 288.031 | 269.9 | 11.31 | OXAZEPAM 2 | 91 | 21 | 12 |
| 302.251 | 284 | 2.59 | OXYMORPHONE 1 | 151 | 25 | 14 |
| 302.251 | 227.1 | 2.59 | OXYMORPHONE 2 | 151 | 37 | 12 |
| 152.016 | 110 | 3.78 | PARACETAMOL 1 | 81 | 21 | 14 |
| 152.016 | 65 | 3.78 | PARACETAMOL 2 | 81 | 39 | 10 |
| 330.014 | 70 | 10.51 | PAROXETINE 1 | 111 | 49 | 10 |
| 330.014 | 44.1 | 10.51 | PAROXETINE 2 | 111 | 71 | 8 |
| 359.081 | 214.2 | 14.60 | PB-22 1 | 71 | 21 | 10 |
| 359.081 | 144.1 | 14.60 | PB-22 2 | 71 | 51 | 16 |
| 248.346 | 91 | 9.17 | PCEEA 1 | 56 | 45 | 12 |
| 248.346 | 90.1 | 9.17 | PCEEA 2 | 56 | 13 | 12 |
| 248.342 | 91 | 8.94 | PCMPA 1 | 66 | 43 | 14 |
| 248.342 | 90.1 | 8.94 | PCMPA 2 | 66 | 15 | 12 |
| 218.345 | 91 | 9.07 | PCPr 1 | 46 | 39 | 6 |
| 218.345 | 159.1 | 9.07 | PCPr 2 | 46 | 15 | 22 |
| 192.062 | 91 | 6.81 | PENTEDRONE 1 | 81 | 31 | 12 |
| 192.062 | 132.1 | 6.81 | PENTEDRONE 2 | 81 | 25 | 16 |
| 194.308 | 176.1 | 6.88 | PENTEDRONE METABOLITE 1 | 81 | 17 | 22 |
| 194.308 | 91 | 6.88 | PENTEDRONE METABOLITE 2 | 81 | 43 | 12 |
| 235.658 | 188.1 | 7.12 | PENTYLONE (bk-MBDP) 1 | 136 | 25 | 24 |
| 235.658 | 218.1 | 7.12 | PENTYLONE (bk-MBDP) 2 | 136 | 19 | 10 |
| 350.845 | 206 | 12.06 | PHENAZEPAM 1 | 131 | 49 | 26 |
| 350.845 | 179 | 12.06 | PHENAZEPAM 2 | 131 | 63 | 22 |
| 244.103 | 86.1 | 8.43 | PHENCYCLIDINE (PCP) 1 | 56 | 17 | 12 |
| 244.103 | 91 | 8.43 | PHENCYCLIDINE (PCP) 2 | 56 | 43 | 12 |
| 231.014 | 42 | 8.46 | PHENOBARBITAL 1 | -100 | -10 | -44 |
| 231.014 | 188 | 8.46 | PHENOBARBITAL 2 | -100 | -10 | -14 |
| 150.067 | 91 | 5.93 | PHENTERMINE 1 | 41 | 27 | 12 |
| 150.067 | 133.1 | 5.93 | PHENTERMINE 2 | 41 | 13 | 16 |
| 253.004 | 182.1 | 10.17 | PHENYTOIN 1 | 116 | 25 | 24 |
| 253.004 | 104.1 | 10.17 | PHENYTOIN 2 | 116 | 45 | 12 |
| 379.06 | 135.1 | 10.20 | PRAVADOLINE 1 | 76 | 23 | 14 |
| 379.06 | 77.1 | 10.20 | PRAVADOLINE 2 | 76 | 89 | 12 |
| 325.365 | 271 | 13.48 | PRAZEPAM 1 | 126 | 31 | 12 |
| 325.365 | 140 | 13.48 | PRAZEPAM 2 | 126 | 49 | 18 |
| 160.078 | 142.1 | 4.61 | PREGABALIN 1 | 116 | 15 | 18 |
| 160.078 | 55 | 4.61 | PREGABALIN 2 | 116 | 29 | 8 |
| 237.063 | 100.1 | 4.01 | PROCAINE 1 | 81 | 21 | 12 |
| 237.063 | 120.1 | 4.01 | PROCAINE 2 | 81 | 37 | 14 |
| 218.162 | 91.1 | 7.95 | PROLINTANE 1 | 86 | 33 | 12 |
| 218.162 | 72.1 | 7.95 | PROLINTANE 2 | 86 | 23 | 10 |
| 285.029 | 86.1 | 10.13 | PROMETHAZINE 1 | 76 | 21 | 12 |
| 285.029 | 71.1 | 10.13 | PROMETHAZINE 2 | 76 | 63 | 10 |
| 342.045 | 116.1 | 10.65 | PROPAFENONE 1 | 86 | 29 | 14 |
| 342.045 | 72.1 | 10.65 | PROPAFENONE 2 | 86 | 47 | 10 |
| 260.069 | 56 | 9.06 | PROPRANOLOL 1 | 86 | 45 | 8 |
| 260.069 | 58.1 | 9.06 | PROPRANOLOL 2 | 86 | 45 | 10 |
| 155.882 | 69.1 | 7.99 | PROPYLHEXEDRINE 1 | 81 | 23 | 10 |
| 155.882 | 55 | 7.99 | PROPYLHEXEDRINE 2 | 81 | 37 | 8 |
| 367.248 | 259.1 | 15.96 | PSB-SB-1202 1 | 121 | 23 | 12 |
| 367.248 | 121.1 | 15.96 | PSB-SB-1202 2 | 121 | 23 | 8 |
| 356.299 | 283 | 12.52 | PTI-1 1 | 106 | 29 | 14 |
| 356.299 | 213.1 | 12.52 | PTI-1 2 | 106 | 47 | 10 |
| 400.341 | 283 | 12.77 | PTI-2 1 | 116 | 31 | 14 |
| 400.341 | 213.1 | 12.77 | PTI-2 2 | 116 | 51 | 10 |
| 260.116 | 91.1 | 9.63 | PV-8 1 | 96 | 33 | 12 |
| 260.116 | 77.1 | 9.63 | PV-8 2 | 96 | 73 | 10 |
| 274.307 | 91 | 10.68 | PV9 1 | 126 | 33 | 12 |
| 274.307 | 77 | 10.68 | PV9 2 | 126 | 73 | 12 |
| 396.09 | 144 | 12.62 | PX-1 1 | 76 | 59 | 18 |
| 396.09 | 231.9 | 12.62 | PX-1 2 | 76 | 31 | 10 |
| 397.253 | 233 | 12.76 | PX-2 1 | 86 | 33 | 12 |
| 397.253 | 352.2 | 12.76 | PX-2 2 | 86 | 21 | 14 |
| 353.943 | 167.1 | 9.35 | PYRAZOLAM 1 | 151 | 47 | 20 |
| 353.943 | 206 | 9.35 | PYRAZOLAM 2 | 151 | 41 | 26 |
| 384.09 | 253.1 | 9.53 | QUETIAPINE 1 | 116 | 31 | 12 |
| 384.09 | 221 | 9.53 | QUETIAPINE 2 | 116 | 51 | 28 |
| 322.05 | 135.1 | 14.62 | RCS-4 1 | 136 | 31 | 16 |
| 322.05 | 77 | 14.62 | RCS-4 2 | 136 | 73 | 10 |
| 326.3 | 121.1 | 8.12 | RH-34 1 | 91 | 25 | 14 |
| 326.3 | 91.1 | 8.12 | RH-34 2 | 91 | 57 | 10 |
| 236.144 | 188.2 | 6.86 | R-MMC 1 | 61 | 25 | 10 |
| 236.144 | 218.1 | 6.86 | R-MMC 2 | 61 | 17 | 18 |
| 303.805 | 138.1 | 4.73 | SCOPOLAMINE 1 | 96 | 29 | 18 |
| 303.805 | 156.1 | 4.73 | SCOPOLAMINE 2 | 96 | 23 | 20 |
| 359.177 | 215.2 | 15.57 | SDB-005 1 | 51 | 21 | 10 |
| 359.177 | 145 | 15.57 | SDB-005 2 | 51 | 45 | 20 |
| 321.153 | 91 | 14.09 | SDB-006 1 | 171 | 61 | 12 |
| 321.153 | 214.1 | 14.09 | SDB-006 2 | 171 | 29 | 10 |
| 306.988 | 276 | 11.51 | SERTRALINE 1 | 66 | 17 | 12 |
| 306.988 | 159 | 11.51 | SERTRALINE 2 | 66 | 37 | 20 |
| 475.047 | 58.1 | 10.22 | SILDENAFIL 1 | 91 | 103 | 10 |
| 475.047 | 100.1 | 10.22 | SILDENAFIL 2 | 91 | 35 | 12 |
| 329.138 | 81.1 | 13.96 | STANOZOLOL 1 | 241 | 79 | 12 |
| 329.138 | 95.1 | 13.96 | STANOZOLOL 2 | 241 | 51 | 12 |
| 222.343 | 107.1 | 7.32 | TAPENTADOL 1 | 86 | 35 | 14 |
| 222.343 | 77 | 7.32 | TAPENTADOL 2 | 86 | 63 | 12 |
| 301.993 | 256 | 11.62 | TEMAZEPAM 1 | 116 | 31 | 12 |
| 301.993 | 284.1 | 11.62 | TEMAZEPAM 2 | 116 | 19 | 12 |
| 265.226 | 176.1 | 9.01 | TETRACAINE 1 | 96 | 21 | 18 |
| 265.226 | 72 | 9.01 | TETRACAINE 2 | 96 | 37 | 10 |
| 315.093 | 193.1 | 15.93 | THC 1 | 106 | 31 | 24 |
| 315.093 | 123 | 15.93 | THC 2 | 106 | 43 | 16 |
| 345.2 | 299.2 | 14.97 | THCCOOH 1 | 106 | 23 | 10 |
| 345.2 | 193.1 | 14.97 | THCCOOH 2 | 120 | 45 | 10 |
| 142.098 | 125 | 3.73 | THIOPROPAMINE 1 | 51 | 13 | 8 |
| 142.098 | 97 | 3.73 | THIOPROPAMINE 2 | 51 | 25 | 12 |
| 359.247 | 215.2 | 15.98 | THJ 1 | 121 | 29 | 10 |
| 359.247 | 145 | 15.98 | THJ 2 | 121 | 49 | 16 |
| 343.076 | 215.1 | 15.53 | THJ-018 1 | 136 | 25 | 10 |
| 343.076 | 145.1 | 15.53 | THJ-018 2 | 136 | 45 | 18 |
| 361.067 | 233.1 | 14.62 | THJ-2201 (5-FLUORO THJ-018) 1 | 131 | 25 | 10 |
| 361.067 | 145.1 | 14.62 | THJ-2201 (5-FLUORO THJ-018) 2 | 131 | 49 | 18 |
| 224.246 | 179 | 5.68 | TILETAMINE 1 | 51 | 13 | 10 |
| 224.246 | 151.1 | 5.68 | TILETAMINE 2 | 51 | 23 | 18 |
| 264.304 | 58.1 | 6.88 | TRAMADOL 1 | 96 | 47 | 8 |
| 264.304 | 42.1 | 6.88 | TRAMADOL 2 | 96 | 113 | 8 |
| 372.082 | 176 | 8.24 | TRAZODONE 1 | 126 | 33 | 24 |
| 372.082 | 148 | 8.24 | TRAZODONE 2 | 126 | 45 | 20 |
| 343.99 | 309.1 | 11.35 | TRIAZOLAM 1 | 106 | 37 | 14 |
| 343.99 | 239.9 | 11.35 | TRIAZOLAM 2 | 106 | 57 | 30 |
| 294.788 | 100.2 | 10.95 | TRIMIPRAMINE 1 | 81 | 23 | 12 |
| 294.788 | 58.1 | 10.95 | TRIMIPRAMINE 2 | 81 | 61 | 8 |
| 329.037 | 284 | 9.09 | U-47700 1 | 56 | 25 | 14 |
| 331.017 | 286 | 9.09 | U-47700 2 | 96 | 25 | 14 |
| 312.107 | 125.1 | 15.56 | UR-144 1 | 96 | 29 | 16 |
| 312.107 | 214 | 15.56 | UR-144 2 | 96 | 33 | 10 |
| 328.092 | 125.1 | 13.77 | UR-144 metabolite 1 | 96 | 27 | 16 |
| 328.092 | 230.1 | 13.77 | UR-144 metabolite 2 | 96 | 33 | 10 |
| 377.154 | 105.1 | 13.30 | W-15 1 | 141 | 33 | 16 |
| 379.165 | 105.2 | 13.30 | W-15 2 | 166 | 33 | 12 |
| 457.176 | 135.1 | 12.36 | WIN 54.461 1 | 126 | 29 | 12 |
| 457.176 | 77 | 12.36 | WIN 54.461 2 | 126 | 95 | 12 |
| 427.117 | 155.1 | 13.63 | WIN 55.212-2 1 | 161 | 33 | 18 |
| 427.117 | 127.2 | 13.63 | WIN 55.212-2 2 | 161 | 75 | 12 |
| 330.293 | 125.1 | 14.69 | XLR-11 1 | 116 | 31 | 16 |
| 330.293 | 232.1 | 14.69 | XLR-11 2 | 116 | 33 | 10 |
| 352.216 | 125.1 | 14.79 | XLR12 1 | 141 | 31 | 8 |
| 352.216 | 254 | 14.79 | XLR12 2 | 141 | 35 | 12 |
| 259.148 | 161 | 11.88 | YANGONIN 1 | 121 | 29 | 22 |
| 259.148 | 89 | 11.88 | YANGONIN 2 | 121 | 93 | 10 |
| 305.911 | 264 | 9.91 | ZALEPLON 1 | 141 | 31 | 12 |
| 305.911 | 236.1 | 9.91 | ZALEPLON 2 | 141 | 37 | 10 |
| 308.323 | 235.1 | 7.76 | ZOLPIDEM 1 | 111 | 47 | 10 |
| 308.323 | 236 | 7.76 | ZOLPIDEM 2 | 111 | 37 | 30 |
| 389.014 | 244.9 | 6.70 | ZOPICLONE 1 | 81 | 23 | 12 |
| 389.014 | 112 | 6.70 | ZOPICLONE 2 | 81 | 79 | 16 |
| 401.024 | 221 | 12.12 | ZUCLOPENTHIXOL 1 | 101 | 73 | 26 |
| 401.024 | 231 | 12.12 | ZUCLOPENTHIXOL 2 | 101 | 49 | 28 |
| 341.989 | 324 | 9.92 | α-HYDROXYMIDAZOLAM 1 | 121 | 29 | 14 |
| 341.989 | 203 | 9.92 | α-HYDROXYMIDAZOLAM 2 | 121 | 37 | 26 |


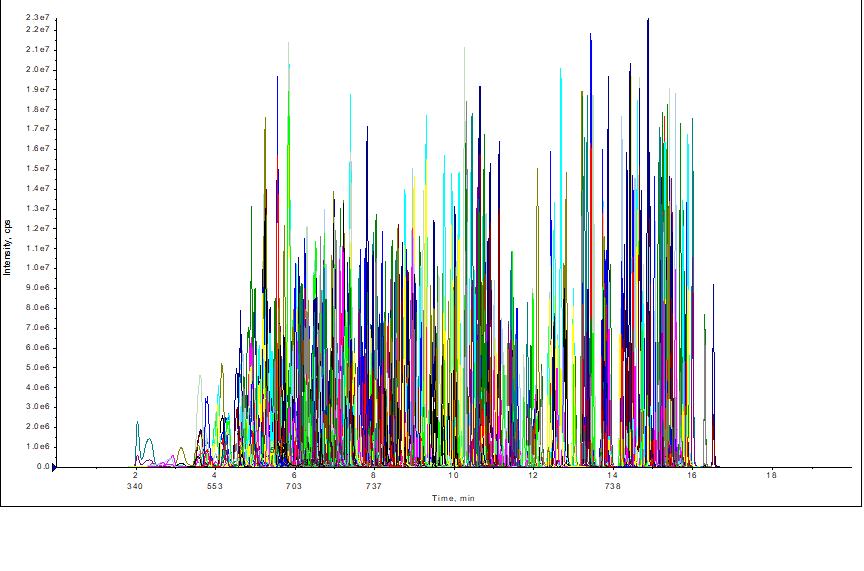


Figure S1. The chromatogram obtained for the Kinetex C18 column (Phenomenex 3.0 x 100 mm; 2.6 μm)

Table 2. The results of precision, BIAS and recovery values for identification of compounds.

| **ANALYTE** | **CONCENTRATION (ng/mg)** | **RECOVERY (%)** | **SD (%)** | **CV (%)** | **BIAS (%)** | **LOQ (ng/mg)** |
| --- | --- | --- | --- | --- | --- | --- |
| 1-(2-METHOXYPHENYL)PIPERAZINE | 0.025 | 134.2 | 10.0 | 7.43 | 34.2 | 0.125 |
|  | 0.125 | 99.0 | 9.2 | 9.29 | -1.0 |  |
|  | 1.25 | 100.3 | 12.9 | 12.89 | 0.3 |  |
|  | 5 | 112.2 | 4.7 | 4.21 | 12.2 |  |
| 1-(3-TRIFLUOROMETHYLPHENYL)PIPERAZINE (TFMPP) | 0.025 | 124.5 | 12.8 | 10.31 | 24.5 | 0.125 |
|  | 0.125 | 106.3 | 6.7 | 6.34 | 6.3 |  |
|  | 1.25 | 99.6 | 5.4 | 5.45 | -0.4 |  |
|  | 5 | 112.9 | 8.2 | 7.29 | 12.9 |  |
| 1-(4-CHLOROPHENYL)PIPERAZINE (pCPP) | 0.025 | 89.1 | 69.9 | 78.45 | -10.9 | 0.125 |
|  | 0.125 | 92.8 | 16.0 | 17.25 | -7.2 |  |
|  | 1.25 | 90.8 | 15.3 | 16.87 | -9.2 |  |
|  | 5 | 100.8 | 20.1 | 19.97 | 0.8 |  |
| 1-(4-FLUOROPHENYL)PIPERAZINE (FPP) | 0.025 | 124.9 | 19.1 | 15.30 | 24.9 | 0.125 |
|  | 0.125 | 95.6 | 5.9 | 6.20 | -4.4 |  |
|  | 1.25 | 94.8 | 8.8 | 9.28 | -5.2 |  |
|  | 5 | 95.8 | 6.8 | 7.11 | -4.2 |  |
| 1.4-DIBENZYLPIPERAZINE (DBZP) | 0.025 | 109.6 | 28.6 | 26.09 | 9.6 | 0.125 |
|  | 0.125 | 103.7 | 14.9 | 14.38 | 3.7 |  |
|  | 1.25 | 90.9 | 10.5 | 11.51 | -9.1 |  |
|  | 5 | 107.3 | 17.8 | 16.59 | 7.3 |  |
| 1-AMINOINDAN | 0.125 | 98.0 | 17.4 | 17.78 | -2.0 | 0.125 |
|  | 1.25 | 103.3 | 11.6 | 11.26 | 3.3 |  |
|  | 5 | 110.2 | 14.2 | 12.88 | 10.2 |  |
| 1-METHYL-4-BENZYLPIPERAZINE (MBZP) | 0.025 | 135.7 | 43.4 | 32.00 | 35.7 | 0.125 |
|  | 0.125 | 98.5 | 12.3 | 12.51 | -1.5 |  |
|  | 1.25 | 80.4 | 5.4 | 6.71 | -19.6 |  |
|  | 5 | 92.2 | 8.5 | 9.24 | -7.8 |  |
| 1-METHYLAMINO-1-(3.4-METHYLENEDIOXYPHENYL)PROPANE | 0.025 | 106.2 | 25.7 | 24.16 | 6.2 | 0.125 |
|  | 0.125 | 108.5 | 13.9 | 12.79 | 8.5 |  |
|  | 1.25 | 116.0 | 11.3 | 9.74 | 16.0 |  |
|  | 5 | 119.2 | 9.3 | 7.76 | 19.2 |  |
| 2.3-DICHLOROPHENYLPIPERAZINE (DCPP) | 0.025 | 125.2 | 26.0 | 20.76 | 25.2 | 0.125 |
|  | 0.125 | 109.1 | 10.3 | 9.40 | 9.1 |  |
|  | 1.25 | 95.7 | 10.0 | 10.49 | -4.3 |  |
|  | 5 | 87.7 | 7.6 | 8.71 | -12.3 |  |
| 2.3-DIMETHYLETHCATHINONE (2.3-DMEC) | 0.025 | 118.1 | 27.0 | 22.88 | 18.1 | 0.125 |
|  | 0.125 | 101.8 | 6.0 | 5.91 | 1.8 |  |
|  | 1.25 | 97.7 | 3.7 | 3.77 | -2.3 |  |
|  | 5 | 102.0 | 10.8 | 10.57 | 2.0 |  |
| 2.3-DIMETHYLMETHCATHINONE (2.3-DMMC) | 0.025 | 109.5 | 38.2 | 34.90 | 9.5 | 0.125 |
|  | 0.125 | 102.1 | 18.9 | 18.47 | 2.1 |  |
|  | 1.25 | 88.0 | 8.3 | 9.44 | -12.0 |  |
|  | 5 | 94.5 | 14.6 | 15.42 | -5.5 |  |
| 2.3-ETHYLONE ISOMER | 0.025 | 125.5 | 17.8 | 14.17 | 25.5 | 0.125 |
|  | 0.125 | 114.6 | 5.5 | 4.82 | 14.6 |  |
|  | 1.25 | 98.5 | 11.1 | 11.30 | -1.5 |  |
|  | 5 | 99.7 | 13.2 | 13.20 | -0.3 |  |
| 2.3-MDMA | 0.025 | 187.9 | 49.7 | 26.47 | 87.9 | 0.125 |
|  | 0.125 | 103.2 | 12.4 | 12.00 | 3.2 |  |
|  | 1.25 | 102.3 | 3.8 | 3.75 | 2.3 |  |
|  | 5 | 99.5 | 7.2 | 7.27 | -0.5 |  |
| 2.3-MDPV | 0.025 | 99.6 | 8.9 | 8.91 | -0.4 | 0.125 |
|  | 0.125 | 101.7 | 4.5 | 4.41 | 1.7 |  |
|  | 1.25 | 104.7 | 8.7 | 8.28 | 4.7 |  |
|  | 5 | 109.6 | 9.2 | 8.39 | 9.6 |  |
| 2.4.5-TRIMETHOXYAMPHETAMINE | 0.125 | 102.5 | 19.1 | 18.67 | 2.5 | 0.125 |
|  | 1.25 | 97.6 | 12.8 | 13.08 | -2.4 |  |
|  | 5 | 93.2 | 5.6 | 5.97 | -6.8 |  |
| 2.4-DIMETHYLMETHCATHINONE (2.4-DMMC) | 0.025 | 95.2 | 29.2 | 30.64 | -4.8 | 0.125 |
|  | 0.125 | 95.6 | 5.7 | 5.96 | -4.4 |  |
|  | 1.25 | 94.6 | 8.4 | 8.88 | -5.4 |  |
|  | 5 | 112.5 | 8.5 | 7.58 | 12.5 |  |
| 2.4-DMEC | 0.025 | 99.1 | 21.9 | 22.06 | -1.0 | 0.125 |
|  | 0.125 | 85.2 | 12.1 | 14.18 | -14.8 |  |
|  | 1.25 | 80.5 | 6.0 | 7.49 | -19.5 |  |
|  | 5 | 89.4 | 13.9 | 15.53 | -10.6 |  |
| 2.5-DMMA | 0.025 | 104.2 | 22.2 | 21.32 | 4.2 | 0.125 |
|  | 0.125 | 106.7 | 10.8 | 10.08 | 6.7 |  |
|  | 1.25 | 112.8 | 6.3 | 5.59 | 12.8 |  |
|  | 5 | 110.6 | 5.6 | 5.03 | 10.6 |  |
| 25B-NBF | 0.025 | 99.4 | 9.9 | 9.96 | -0.6 | 0.125 |
|  | 0.125 | 93.4 | 7.9 | 8.44 | -6.6 |  |
|  | 1.25 | 81.4 | 14.8 | 18.17 | -18.6 |  |
|  | 5 | 103.7 | 8.9 | 8.55 | 3.7 |  |
| 25C-NBF | 0.025 | 110.4 | 11.6 | 10.49 | 10.4 | 0.125 |
|  | 0.125 | 107.3 | 11.1 | 10.35 | 7.3 |  |
|  | 1.25 | 100.2 | 12.9 | 12.87 | 0.2 |  |
|  | 5 | 110.3 | 8.9 | 8.06 | 10.3 |  |
| 25C-NBOH | 0.025 | 108.2 | 20.8 | 19.19 | 8.2 | 0.125 |
|  | 0.125 | 98.7 | 15.5 | 15.74 | -1.3 |  |
|  | 1.25 | 87.6 | 12.0 | 13.71 | -12.4 |  |
|  | 5 | 101.4 | 10.7 | 10.55 | 1.4 |  |
| 25C-NBOMe | 0.025 | 101.7 | 12.7 | 12.48 | 1.7 | 0.125 |
|  | 0.125 | 99.8 | 10.5 | 10.57 | -0.2 |  |
|  | 1.25 | 88.5 | 14.0 | 15.79 | -11.5 |  |
|  | 5 | 111.7 | 7.2 | 6.44 | 11.7 |  |
| 25D-NBOMe | 0.025 | 109.2 | 9.5 | 8.68 | 9.2 | 0.125 |
|  | 0.125 | 107.2 | 8.5 | 7.89 | 7.2 |  |
|  | 1.25 | 91.0 | 9.2 | 10.15 | -9.0 |  |
|  | 5 | 101.7 | 13.0 | 12.77 | 1.7 |  |
| 25E-NBOMe | 0.025 | 102.6 | 12.6 | 12.26 | 2.6 | 0.125 |
|  | 0.125 | 108.2 | 5.0 | 4.64 | 8.2 |  |
|  | 1.25 | 89.9 | 12.5 | 13.86 | -10.1 |  |
|  | 5 | 113.7 | 9.6 | 8.41 | 13.7 |  |
| 25G-NBOMe | 0.025 | 110.6 | 9.1 | 8.23 | 10.6 | 0.125 |
|  | 0.125 | 107.9 | 4.9 | 4.57 | 7.9 |  |
|  | 1.25 | 97.7 | 9.8 | 10.00 | -2.3 |  |
|  | 5 | 113.0 | 12.1 | 10.70 | 13.0 |  |
| 25H-NBOMe | 0.025 | 110.4 | 10.9 | 9.87 | 10.4 | 0.125 |
|  | 0.125 | 110.8 | 5.4 | 4.88 | 10.8 |  |
|  | 1.25 | 101.3 | 4.8 | 4.78 | 1.3 |  |
|  | 5 | 111.1 | 7.4 | 6.62 | 11.1 |  |
| 25-I-NB2OMe | 0.025 | 102.1 | 11.6 | 11.32 | 2.1 | 0.125 |
|  | 0.125 | 102.2 | 18.5 | 18.10 | 2.2 |  |
|  | 1.25 | 81.4 | 11.6 | 14.22 | -18.6 |  |
|  | 5 | 115.1 | 14.2 | 12.35 | 15.1 |  |
| 25I-NB3OMe | 0.025 | 71.7 | 15.6 | 21.74 | -28.3 | 0.125 |
|  | 0.125 | 120.0 | 5.8 | 4.81 | 20.0 |  |
|  | 1.25 | 83.8 | 11.1 | 13.23 | -16.2 |  |
|  | 5 | 100.7 | 10.5 | 10.42 | 0.7 |  |
| 25I-NB4OMe | 0.025 | 99.2 | 14.7 | 14.78 | -0.8 | 0.125 |
|  | 0.125 | 119.6 | 17.1 | 14.33 | 19.6 |  |
|  | 1.25 | 91.5 | 12.2 | 13.31 | -8.5 |  |
|  | 5 | 118.9 | 5.8 | 4.89 | 18.9 |  |
| 25I-NBF | 0.025 | 89.5 | 14.9 | 16.64 | -10.6 | 0.125 |
|  | 0.125 | 108.1 | 12.0 | 11.14 | 8.1 |  |
|  | 1.25 | 92.3 | 7.3 | 7.95 | -7.7 |  |
|  | 5 | 110.5 | 11.7 | 10.60 | 10.5 |  |
| 25I-NBMD | 0.025 | 97.1 | 14.1 | 14.56 | -2.9 | 0.125 |
|  | 0.125 | 96.3 | 6.4 | 6.66 | -3.7 |  |
|  | 1.25 | 97.6 | 6.5 | 6.64 | -2.4 |  |
|  | 5 | 110.1 | 14.2 | 12.86 | 10.1 |  |
| 25I-NBOH | 0.025 | 106.0 | 7.0 | 6.58 | 6.0 | 0.125 |
|  | 0.125 | 102.7 | 9.6 | 9.34 | 2.7 |  |
|  | 1.25 | 83.5 | 4.8 | 5.72 | -16.5 |  |
|  | 5 | 98.3 | 6.9 | 7.00 | -1.7 |  |
| 25N-NBOMe | 0.025 | 90.2 | 12.6 | 13.97 | -9.8 | 0.125 |
|  | 0.125 | 105.5 | 9.9 | 9.35 | 5.5 |  |
|  | 1.25 | 96.8 | 8.9 | 9.16 | -3.2 |  |
|  | 5 | 108.6 | 8.0 | 7.38 | 8.6 |  |
| 25T2-NBOMe | 0.025 | 88.5 | 14.8 | 16.73 | -11.5 | 0.125 |
|  | 0.125 | 99.8 | 8.7 | 8.73 | -0.2 |  |
|  | 1.25 | 91.3 | 10.7 | 11.75 | -8.7 |  |
|  | 5 | 107.8 | 11.7 | 10.88 | 7.8 |  |
| 25T-NBOMe | 0.025 | 107.3 | 10.0 | 9.30 | 7.3 | 0.125 |
|  | 0.125 | 100.7 | 3.7 | 3.63 | 0.7 |  |
|  | 1.25 | 95.0 | 3.0 | 3.20 | -5.0 |  |
|  | 5 | 114.9 | 10.7 | 9.33 | 14.9 |  |
| 2-AMINO-1-PHENYLBUTANE | 0.025 | 113.1 | 19.7 | 17.42 | 13.1 | 0.125 |
|  | 0.125 | 99.7 | 15.6 | 15.66 | -0.3 |  |
|  | 1.25 | 99.0 | 12.2 | 12.30 | -1.0 |  |
|  | 5 | 106.5 | 15.6 | 14.61 | 6.5 |  |
| 2-AMINOINDANE | 0.025 | 125.3 | 32.1 | 25.61 | 25.3 | 0.125 |
|  | 0.125 | 104.1 | 9.0 | 8.61 | 4.1 |  |
|  | 1.25 | 97.1 | 6.6 | 6.74 | -2.9 |  |
|  | 5 | 102.7 | 8.9 | 8.67 | 2.7 |  |
| 2-BROMOAMPHETAMINE | 0.025 | 123.5 | 26.7 | 21.63 | 23.5 | 0.125 |
|  | 0.125 | 115.4 | 8.3 | 7.18 | 15.4 |  |
|  | 1.25 | 109.5 | 10.1 | 9.19 | 9.5 |  |
|  | 5 | 107.5 | 11.9 | 11.08 | 7.5 |  |
| 2-BROMOMETHAMPHETAMINE | 0.025 | 107.0 | 22.7 | 21.26 | 7.0 | 0.125 |
|  | 0.125 | 109.6 | 9.1 | 8.31 | 9.6 |  |
|  | 1.25 | 107.1 | 16.0 | 14.93 | 7.1 |  |
|  | 5 | 113.2 | 10.5 | 9.25 | 13.2 |  |
| 2C-B | 0.025 | 47.7 | 30.3 | 63.44 | -52.3 | 0.125 |
|  | 0.125 | 83.0 | 9.3 | 11.22 | -17.0 |  |
|  | 1.25 | 90.3 | 11.1 | 12.32 | -9.7 |  |
|  | 5 | 113.2 | 4.6 | 4.09 | 13.2 |  |
| 2C-B_FLY | 0.025 | 99.7 | 25.9 | 25.97 | -0.3 | 0.125 |
|  | 0.125 | 119.2 | 19.6 | 16.46 | 19.2 |  |
|  | 1.25 | 102.4 | 8.9 | 8.64 | 2.4 |  |
|  | 5 | 112.5 | 18.0 | 16.04 | 12.5 |  |
| 2C-C | 0.025 | 176.0 | 125.3 | 71.22 | 76.0 | 0.125 |
|  | 0.125 | 98.1 | 16.6 | 16.96 | -1.9 |  |
|  | 1.25 | 93.5 | 13.1 | 13.96 | -6.5 |  |
|  | 5 | 94.3 | 6.7 | 7.11 | -5.7 |  |
| 2C-D | 0.025 | 1513.1 | 929.0 | 61.40 | 1413.1 | - |
|  | 0.125 | 307.3 | 181.5 | 59.07 | 207.3 |  |
|  | 1.25 | 129.3 | 59.1 | 45.69 | 29.3 |  |
|  | 5 | 168.5 | 66.0 | 39.18 | 68.5 |  |
| 2C-G | 0.025 | 95.1 | 22.9 | 24.12 | -4.9 | 0.125 |
|  | 0.125 | 103.4 | 19.5 | 18.86 | 3.4 |  |
|  | 1.25 | 86.3 | 8.8 | 10.17 | -13.7 |  |
|  | 5 | 101.0 | 12.1 | 11.97 | 1.0 |  |
| 2-CHLOROAMPHETAMINE | 0.025 | 101.9 | 16.5 | 16.23 | 1.9 | 0.125 |
|  | 0.125 | 111.1 | 9.3 | 8.38 | 11.1 |  |
|  | 1.25 | 100.6 | 15.3 | 15.17 | 0.6 |  |
|  | 5 | 108.1 | 11.0 | 10.20 | 8.1 |  |
| 2C-I | 0.025 | 114.7 | 35.3 | 30.77 | 14.7 | 0.125 |
|  | 0.125 | 87.6 | 14.1 | 16.14 | -12.4 |  |
|  | 1.25 | 88.3 | 11.2 | 12.64 | -11.7 |  |
|  | 5 | 114.8 | 12.3 | 10.73 | 14.8 |  |
| 2C-P | 0.025 | 179.1 | 121.1 | 67.61 | 79.1 | 0.125 |
|  | 0.125 | 109.0 | 14.4 | 13.26 | 9.0 |  |
|  | 1.25 | 92.0 | 12.6 | 13.70 | -8.0 |  |
|  | 5 | 113.0 | 16.7 | 14.75 | 13.0 |  |
| 2C-T-7 | 0.025 | 125.1 | 30.6 | 24.48 | 25.1 | 0.125 |
|  | 0.125 | 94.6 | 11.2 | 11.81 | -5.4 |  |
|  | 1.25 | 105.4 | 13.0 | 12.30 | 5.4 |  |
|  | 5 | 118.1 | 18.8 | 15.94 | 18.1 |  |
| 2C-TFM | 0.025 | 125.6 | 31.0 | 24.69 | 25.6 | 0.125 |
|  | 0.125 | 115.7 | 12.0 | 10.41 | 15.7 |  |
|  | 1.25 | 92.5 | 18.5 | 19.99 | -7.5 |  |
|  | 5 | 100.0 | 14.9 | 14.88 | 0.0 |  |
| 2-FEC | 0.025 | 81.3 | 24.2 | 29.82 | -18.7 | 0.125 |
|  | 0.125 | 94.5 | 13.7 | 14.54 | -5.5 |  |
|  | 1.25 | 80.3 | 5.7 | 7.08 | -19.7 |  |
|  | 5 | 83.2 | 4.9 | 5.91 | -16.8 |  |
| 2-FIC | 0.025 | 104.4 | 21.3 | 20.36 | 4.4 | 0.125 |
|  | 0.125 | 99.5 | 13.2 | 13.31 | -0.5 |  |
|  | 1.25 | 110.4 | 9.2 | 8.30 | 10.4 |  |
|  | 5 | 110.2 | 7.3 | 6.62 | 10.2 |  |
| 2-FLUOROAMPHETAMINE | 0.025 | 110.4 | 9.2 | 8.36 | 10.4 | 0.125 |
|  | 0.125 | 103.2 | 10.9 | 10.57 | 3.2 |  |
|  | 1.25 | 107.8 | 15.5 | 14.38 | 7.8 |  |
|  | 5 | 111.0 | 7.2 | 6.45 | 11.0 |  |
| 2-FLUOROMETHAMPHETAMINE (2-FMA) | 0.025 | 120.8 | 17.3 | 14.35 | 20.8 | 0.125 |
|  | 0.125 | 113.0 | 8.1 | 7.21 | 13.0 |  |
|  | 1.25 | 102.7 | 5.3 | 5.14 | 2.7 |  |
|  | 5 | 105.2 | 7.8 | 7.36 | 5.2 |  |
| 2-FLUOROMETHCATHINONE (2-FMC) | 0.025 | 97.7 | 20.8 | 21.28 | -2.3 | 0.125 |
|  | 0.125 | 96.8 | 4.8 | 4.93 | -3.2 |  |
|  | 1.25 | 89.0 | 4.0 | 4.48 | -11.0 |  |
|  | 5 | 86.2 | 9.2 | 10.66 | -13.8 |  |
| 2-IODOAMPHETAMINE | 0.025 | 109.0 | 25.8 | 23.66 | 9.0 | 0.125 |
|  | 0.125 | 111.1 | 12.6 | 11.35 | 11.1 |  |
|  | 1.25 | 88.7 | 6.2 | 6.95 | -11.3 |  |
|  | 5 | 96.7 | 8.7 | 9.02 | -3.3 |  |
| 2-MAPB | 0.025 | 85.2 | 22.2 | 26.08 | -14.8 | 0.125 |
|  | 0.125 | 98.8 | 15.9 | 16.09 | -1.2 |  |
|  | 1.25 | 93.3 | 12.8 | 13.70 | -6.7 |  |
|  | 5 | 92.4 | 10.4 | 11.22 | -7.6 |  |
| 2-MeOMC | 0.025 | 100.1 | 22.4 | 22.40 | 0.1 | 0.125 |
|  | 0.125 | 111.9 | 7.0 | 6.27 | 11.9 |  |
|  | 1.25 | 94.2 | 10.5 | 11.10 | -5.8 |  |
|  | 5 | 103.3 | 9.2 | 8.86 | 3.3 |  |
| 2-METHOXY-2-PHENYLETHYLAMINE | 0.025 | 79.4 | 15.2 | 19.09 | -20.6 | 0.125 |
|  | 0.125 | 90.9 | 11.6 | 12.81 | -9.1 |  |
|  | 1.25 | 99.6 | 8.0 | 8.02 | -0.4 |  |
|  | 5 | 100.6 | 9.5 | 9.44 | 0.6 |  |
| 2-METHOXYAMPHETAMINE (2-MA) | 0.025 | 111.2 | 12.3 | 11.07 | 11.2 | 0.125 |
|  | 0.125 | 96.7 | 11.0 | 11.41 | -3.3 |  |
|  | 1.25 | 98.9 | 12.8 | 12.95 | -1.1 |  |
|  | 5 | 109.3 | 7.3 | 6.69 | 9.3 |  |
| 2-METHOXYMETHAMPHETAMINE (2-MeOMA) | 0.025 | 45.6 | 15.5 | 33.87 | -54.4 | 0.125 |
|  | 0.125 | 101.7 | 18.9 | 18.60 | 1.7 |  |
|  | 1.25 | 81.6 | 7.0 | 8.61 | -18.4 |  |
|  | 5 | 93.9 | 9.3 | 9.85 | -6.1 |  |
| 2-METHYLAMINO-1-PHENYLBUTANE | 0.025 | 56.4 | 30.7 | 54.54 | -43.6 | 0.125 |
|  | 0.125 | 100.2 | 13.0 | 12.98 | 0.2 |  |
|  | 1.25 | 99.3 | 11.9 | 11.94 | -0.7 |  |
|  | 5 | 98.6 | 10.6 | 10.79 | -1.4 |  |
| 2-METHYLMETHCATHINONE (2-MMC) | 0.025 | 104.6 | 18.2 | 17.35 | 4.6 | 0.125 |
|  | 0.125 | 93.3 | 5.4 | 5.76 | -6.7 |  |
|  | 1.25 | 97.5 | 10.4 | 10.67 | -2.5 |  |
|  | 5 | 102.0 | 14.2 | 13.89 | 2.0 |  |
| 2-METHYL-PBP | 0.025 | 124.7 | 41.6 | 33.37 | 24.7 | 0.125 |
|  | 0.125 | 96.8 | 9.2 | 9.53 | -3.2 |  |
|  | 1.25 | 91.4 | 11.9 | 12.98 | -8.6 |  |
|  | 5 | 107.0 | 6.7 | 6.23 | 7.0 |  |
| 2-METHYL-PPP | 0.025 | 106.0 | 23.0 | 21.71 | 6.0 | 0.125 |
|  | 0.125 | 92.9 | 9.5 | 10.21 | -7.1 |  |
|  | 1.25 | 91.2 | 13.9 | 15.26 | -8.8 |  |
|  | 5 | 105.4 | 9.1 | 8.65 | 5.4 |  |
| 3.4-DICHLOROMETHYLPHENIDATE (3.4-CTMP) | 0.025 | 115.9 | 29.4 | 25.41 | 15.9 | 0.125 |
|  | 0.125 | 102.3 | 15.1 | 14.80 | 2.3 |  |
|  | 1.25 | 94.6 | 13.3 | 14.02 | -5.4 |  |
|  | 5 | 115.5 | 14.3 | 12.41 | 15.5 |  |
| 3.4-DIMETHOXY-ALPHA-PVP | 0.025 | 80.6 | 14.8 | 18.35 | -19.4 | 0.125 |
|  | 0.125 | 106.6 | 8.9 | 8.38 | 6.6 |  |
|  | 1.25 | 107.3 | 8.2 | 7.67 | 7.3 |  |
|  | 5 | 119.5 | 9.9 | 8.29 | 19.5 |  |
| 3.4-DIMETHYLMETHCATHINONE (3.4-DMMC) | 0.025 | 89.8 | 26.1 | 29.06 | -10.2 | 0.125 |
|  | 0.125 | 108.3 | 19.0 | 17.55 | 8.3 |  |
|  | 1.25 | 100.4 | 13.5 | 13.42 | 0.4 |  |
|  | 5 | 109.7 | 11.6 | 10.54 | 9.7 |  |
| 3.4-DMEC | 0.025 | 89.4 | 29.7 | 33.15 | -10.6 | 0.125 |
|  | 0.125 | 97.5 | 8.6 | 8.80 | -2.5 |  |
|  | 1.25 | 106.2 | 13.6 | 12.84 | 6.2 |  |
|  | 5 | 111.5 | 16.0 | 14.35 | 11.5 |  |
| 3.4-DMMA | 0.025 | 112.1 | 21.3 | 19.04 | 12.1 | 0.125 |
|  | 0.125 | 105.7 | 12.1 | 11.40 | 5.7 |  |
|  | 1.25 | 116.4 | 11.1 | 9.56 | 16.4 |  |
|  | 5 | 117.4 | 7.5 | 6.42 | 17.4 |  |
| 3.4-EDMA | 0.025 | 101.9 | 14.7 | 14.42 | 1.9 | 0.125 |
|  | 0.125 | 95.8 | 7.4 | 7.74 | -4.2 |  |
|  | 1.25 | 97.6 | 12.6 | 12.91 | -2.4 |  |
|  | 5 | 110.2 | 12.8 | 11.62 | 10.2 |  |
| 3.4-EDMC | 0.025 | 94.8 | 21.3 | 22.43 | -5.2 | 0.125 |
|  | 0.125 | 100.6 | 12.3 | 12.24 | 0.6 |  |
|  | 1.25 | 89.7 | 9.1 | 10.12 | -10.3 |  |
|  | 5 | 101.7 | 7.4 | 7.26 | 1.7 |  |
| 3.4-MDMA (ECSTAZY) | 0.025 | 86.9 | 12.6 | 14.50 | -13.1 | 0.125 |
|  | 0.125 | 95.6 | 13.3 | 13.87 | -4.4 |  |
|  | 1.25 | 114.2 | 7.7 | 6.72 | 14.2 |  |
|  | 5 | 116.8 | 11.9 | 10.19 | 16.8 |  |
| 3.4-MDPA | 0.025 | 134.5 | 29.3 | 21.82 | 34.5 | 0.125 |
|  | 0.125 | 111.6 | 9.5 | 8.48 | 11.6 |  |
|  | 1.25 | 102.0 | 8.3 | 8.14 | 2.0 |  |
|  | 5 | 119.7 | 14.1 | 11.76 | 19.7 |  |
| 3.4-MDPHP | 0.025 | 84.5 | 26.7 | 31.57 | -15.5 | 0.125 |
|  | 0.125 | 88.7 | 9.4 | 10.56 | -11.3 |  |
|  | 1.25 | 91.4 | 11.0 | 12.05 | -8.6 |  |
|  | 5 | 108.6 | 14.9 | 13.71 | 8.6 |  |
| 3.4-METHYLENDIOXYPYROVALERONE | 0.025 | 83.6 | 16.0 | 19.13 | -16.4 | 0.125 |
|  | 0.125 | 101.4 | 15.7 | 15.49 | 1.4 |  |
|  | 1.25 | 107.1 | 8.4 | 7.83 | 7.1 |  |
|  | 5 | 117.2 | 8.9 | 7.60 | 17.2 |  |
| 3.4-METHYLENEDIOXY_PV9 | 0.025 | 107.9 | 5.5 | 5.14 | 7.9 | 0.125 |
|  | 0.125 | 113.5 | 6.6 | 5.83 | 13.5 |  |
|  | 1.25 | 96.5 | 7.5 | 7.80 | -3.5 |  |
|  | 5 | 109.4 | 11.2 | 10.25 | 9.4 |  |
| 30C-NBOMe | 0.025 | 96.0 | 10.4 | 10.80 | -4.0 | 0.125 |
|  | 0.125 | 95.4 | 9.0 | 9.41 | -4.6 |  |
|  | 1.25 | 101.0 | 18.3 | 18.12 | 1.0 |  |
|  | 5 | 116.9 | 9.8 | 8.39 | 16.9 |  |
| 3-BROMOAMPHETAMINE | 0.025 | 149.7 | 55.0 | 36.72 | 49.7 | 0.125 |
|  | 0.125 | 114.9 | 11.5 | 9.99 | 14.9 |  |
|  | 1.25 | 117.9 | 11.8 | 10.02 | 17.9 |  |
|  | 5 | 112.3 | 21.2 | 18.87 | 12.3 |  |
| 3-BROMOMETHAMPHETAMINE | 0.025 | 106.8 | 10.1 | 9.44 | 6.8 | 0.125 |
|  | 0.125 | 106.2 | 14.8 | 13.89 | 6.2 |  |
|  | 1.25 | 105.5 | 16.7 | 15.81 | 5.5 |  |
|  | 5 | 109.7 | 13.8 | 12.60 | 9.7 |  |
| 3-BROMOMETHCATHINONE (3-BMC) | 0.025 | 90.6 | 15.8 | 17.47 | -9.4 | 0.125 |
|  | 0.125 | 98.4 | 6.8 | 6.90 | -1.6 |  |
|  | 1.25 | 94.3 | 6.0 | 6.36 | -5.7 |  |
|  | 5 | 97.4 | 8.0 | 8.23 | -2.6 |  |
| 3-CAF | 0.025 | 118.8 | 13.6 | 11.48 | 18.8 | 0.125 |
|  | 0.125 | 118.0 | 5.9 | 4.97 | 18.0 |  |
|  | 1.25 | 181.2 | 21.3 | 11.73 | 81.2 |  |
| 3C-B-FLY | 0.025 | 84.3 | 26.1 | 30.91 | -15.7 | 0.125 |
|  | 0.125 | 93.5 | 14.1 | 15.04 | -6.5 |  |
|  | 1.25 | 93.0 | 12.8 | 13.76 | -7.0 |  |
|  | 5 | 108.2 | 5.8 | 5.36 | 8.2 |  |
| 3-CHLOROAMPHETAMINE | 0.025 | 99.6 | 56.3 | 56.47 | -0.4 | 0.125 |
|  | 0.125 | 86.6 | 16.9 | 19.51 | -13.4 |  |
|  | 1.25 | 96.8 | 12.4 | 12.79 | -3.2 |  |
|  | 5 | 105.9 | 11.2 | 10.54 | 5.9 |  |
| 3-CHLOROMETHCATHINONE (3-CMC) | 0.025 | 96.1 | 14.9 | 15.48 | -3.9 | 0.125 |
|  | 0.125 | 105.5 | 9.9 | 9.40 | 5.5 |  |
|  | 1.25 | 98.8 | 12.7 | 12.85 | -1.2 |  |
|  | 5 | 93.0 | 11.2 | 12.06 | -7.0 |  |
| 3-CHLOROPHENYLPIPERAZINE (mCPP) | 0.025 | 105.6 | 21.9 | 20.76 | 5.6 | 0.125 |
|  | 0.125 | 89.4 | 4.8 | 5.38 | -10.6 |  |
|  | 1.25 | 94.7 | 9.3 | 9.82 | -5.3 |  |
|  | 5 | 90.6 | 9.3 | 10.23 | -9.4 |  |
| 3C-P | 0.025 | 108.8 | 12.7 | 11.68 | 8.8 | 0.125 |
|  | 0.125 | 85.6 | 9.3 | 10.90 | -14.4 |  |
|  | 1.25 | 99.8 | 17.4 | 17.45 | -0.2 |  |
|  | 5 | 99.1 | 6.3 | 6.37 | -0.9 |  |
| 3-DESOXY-3.4-MDPV | 0.025 | 97.0 | 13.9 | 14.35 | -3.0 | 0.125 |
|  | 0.125 | 92.8 | 9.2 | 9.88 | -7.2 |  |
|  | 1.25 | 92.3 | 10.6 | 11.45 | -7.7 |  |
|  | 5 | 92.4 | 11.1 | 12.07 | -7.6 |  |
| 3-ETHYLMETHCATHINONE (3-EMC) | 0.025 | 97.2 | 7.0 | 7.23 | -2.8 | 0.125 |
|  | 0.125 | 97.4 | 11.7 | 12.02 | -2.6 |  |
|  | 1.25 | 109.4 | 15.3 | 13.98 | 9.4 |  |
|  | 5 | 100.4 | 6.1 | 6.06 | 0.4 |  |
| 3-FEC | 0.025 | 149.7 | 16.1 | 10.78 | 49.7 | 0.125 |
|  | 0.125 | 117.1 | 10.2 | 8.70 | 17.1 |  |
|  | 1.25 | 103.7 | 6.1 | 5.89 | 3.7 |  |
|  | 5 | 104.3 | 8.9 | 8.57 | 4.3 |  |
| 3-FLUORO-ALFA-PPP | 0.025 | 116.1 | 9.5 | 8.18 | 16.1 | 0.125 |
|  | 0.125 | 99.5 | 7.6 | 7.67 | -0.5 |  |
|  | 1.25 | 104.4 | 9.3 | 8.91 | 4.4 |  |
|  | 5 | 111.3 | 11.9 | 10.65 | 11.3 |  |
| 3-FLUOROAMPHETAMINE | 0.025 | 72.6 | 9.0 | 12.37 | -27.4 | 0.125 |
|  | 0.125 | 92.0 | 13.8 | 15.05 | -8.0 |  |
|  | 1.25 | 99.2 | 5.5 | 5.59 | -0.8 |  |
|  | 5 | 107.9 | 17.1 | 15.84 | 7.9 |  |
| 3-FLUOROMETHAMPHETAMINE (3-FMA) | 0.025 | 127.1 | 17.0 | 13.42 | 27.1 | 0.125 |
|  | 0.125 | 106.1 | 7.7 | 7.27 | 6.1 |  |
|  | 1.25 | 96.7 | 7.0 | 7.26 | -3.3 |  |
|  | 5 | 103.7 | 4.1 | 3.93 | 3.7 |  |
| 3-FLUOROMETHCATHINONE (3-FMC) | 0.025 | 96.4 | 17.1 | 17.76 | -3.6 | 0.125 |
|  | 0.125 | 95.2 | 3.8 | 3.96 | -4.8 |  |
|  | 1.25 | 95.3 | 7.6 | 7.94 | -4.7 |  |
|  | 5 | 94.7 | 5.7 | 6.02 | -5.3 |  |
| 3-HYDROXYBROMAZEPAM | 0.025 | 87.5 | 33.2 | 37.98 | -12.5 | 0.125 |
|  | 0.125 | 83.4 | 12.8 | 15.30 | -16.6 |  |
|  | 1.25 | 90.5 | 10.0 | 11.06 | -9.5 |  |
|  | 5 | 98.2 | 8.2 | 8.32 | -1.8 |  |
| 3-HYDROXYFLUNITRAZEPAM | 0.125 | 93.6 | 17.6 | 18.81 | -6.4 | 0.125 |
|  | 1.25 | 97.9 | 12.4 | 12.66 | -2.1 |  |
|  | 5 | 110.3 | 6.7 | 6.04 | 10.3 |  |
| 3-IODOAMPHETAMINE | 0.025 | 92.2 | 25.1 | 27.27 | -7.8 | 0.125 |
|  | 0.125 | 119.6 | 7.1 | 5.96 | 19.6 |  |
|  | 1.25 | 109.5 | 11.9 | 10.89 | 9.5 |  |
|  | 5 | 101.5 | 15.6 | 15.36 | 1.5 |  |
| 3-MEC | 0.025 | 125.1 | 29.0 | 23.15 | 25.1 | 0.125 |
|  | 0.125 | 105.1 | 11.9 | 11.33 | 5.1 |  |
|  | 1.25 | 92.2 | 12.3 | 13.35 | -7.8 |  |
|  | 5 | 104.1 | 13.3 | 12.74 | 4.1 |  |
| 3-MeOMC | 0.025 | 93.9 | 10.7 | 11.42 | -6.1 | 0.125 |
|  | 0.125 | 91.1 | 8.8 | 9.65 | -8.9 |  |
|  | 1.25 | 89.7 | 4.7 | 5.20 | -10.3 |  |
|  | 5 | 88.7 | 8.7 | 9.85 | -11.3 |  |
| 3-METHOXYAMPHETAMINE (3-MA) | 0.025 | 134.1 | 20.4 | 15.23 | 34.1 | 0.125 |
|  | 0.125 | 94.0 | 18.1 | 19.31 | -6.0 |  |
|  | 1.25 | 109.6 | 6.1 | 5.58 | 9.6 |  |
|  | 5 | 109.3 | 10.8 | 9.87 | 9.3 |  |
| 3-METHOXYPHENCYCLIDINE | 0.025 | 99.9 | 21.5 | 21.54 | -0.1 | 0.125 |
|  | 0.125 | 110.8 | 11.2 | 10.11 | 10.8 |  |
|  | 1.25 | 85.3 | 6.2 | 7.31 | -14.7 |  |
|  | 5 | 107.2 | 6.3 | 5.88 | 7.2 |  |
| 3-METHYLMETHCATHINONE (3-MMC) | 0.025 | 100.4 | 7.9 | 7.91 | 0.4 | 0.125 |
|  | 0.125 | 95.4 | 8.4 | 8.77 | -4.6 |  |
|  | 1.25 | 108.8 | 5.9 | 5.40 | 8.8 |  |
|  | 5 | 105.3 | 9.1 | 8.64 | 5.3 |  |
| 3-METHYL-PBP | 0.025 | 103.9 | 14.3 | 13.76 | 3.9 | 0.125 |
|  | 0.125 | 105.0 | 5.8 | 5.55 | 5.0 |  |
|  | 1.25 | 99.1 | 12.2 | 12.35 | -0.9 |  |
|  | 5 | 105.4 | 7.6 | 7.23 | 5.4 |  |
| 3-METHYL-PPP | 0.025 | 109.0 | 27.1 | 24.87 | 9.0 | 0.125 |
|  | 0.125 | 106.0 | 9.0 | 8.52 | 6.0 |  |
|  | 1.25 | 103.2 | 8.9 | 8.62 | 3.2 |  |
|  | 5 | 108.6 | 12.3 | 11.36 | 8.6 |  |
| 4.4'-DMAR | 0.025 | 82.0 | 11.6 | 14.09 | -18.0 | 0.125 |
|  | 0.125 | 95.0 | 11.6 | 12.25 | -5.0 |  |
|  | 1.25 | 107.9 | 6.7 | 6.20 | 7.9 |  |
|  | 5 | 115.0 | 13.8 | 11.97 | 15.0 |  |
| 4-AcO-DET | 0.025 | 86.4 | 17.9 | 20.66 | -13.6 | 0.125 |
|  | 0.125 | 109.6 | 9.4 | 8.56 | 9.6 |  |
|  | 1.25 | 94.5 | 18.0 | 19.03 | -5.5 |  |
|  | 5 | 100.3 | 11.7 | 11.71 | 0.3 |  |
| 4-AcO-DMT | 0.025 | 108.5 | 23.9 | 21.98 | 8.5 | 0.125 |
|  | 0.125 | 108.3 | 11.2 | 10.32 | 8.3 |  |
|  | 1.25 | 91.0 | 14.9 | 16.37 | -9.0 |  |
|  | 5 | 85.5 | 10.4 | 12.22 | -14.5 |  |
| 4-AcO-MET | 0.025 | 72.2 | 31.6 | 43.79 | -27.8 | 0.125 |
|  | 0.125 | 105.1 | 10.9 | 10.34 | 5.1 |  |
|  | 1.25 | 96.8 | 13.8 | 14.30 | -3.2 |  |
|  | 5 | 108.4 | 12.5 | 11.54 | 8.4 |  |
| 4-APB | 0.025 | 110.7 | 12.9 | 11.61 | 10.7 | 0.125 |
|  | 0.125 | 94.5 | 10.1 | 10.71 | -5.5 |  |
|  | 1.25 | 109.7 | 20.1 | 18.33 | 9.7 |  |
|  | 5 | 115.0 | 14.6 | 12.67 | 15.0 |  |
| 4-APDB | 0.025 | 163.7 | 110.3 | 67.38 | 63.7 | 0.125 |
|  | 0.125 | 117.4 | 12.6 | 10.75 | 17.4 |  |
|  | 1.25 | 98.0 | 12.2 | 12.42 | -2.0 |  |
|  | 5 | 105.8 | 12.7 | 11.97 | 5.8 |  |
| 4-BROMO-2.5-DMMA | 0.025 | 105.3 | 21.6 | 20.52 | 5.3 | 0.125 |
|  | 0.125 | 102.8 | 16.3 | 15.84 | 2.8 |  |
|  | 1.25 | 105.3 | 17.0 | 16.16 | 5.3 |  |
|  | 5 | 115.5 | 14.6 | 12.66 | 15.5 |  |
| 4-BROMOAMPHETAMINE | 0.025 | 109.8 | 45.6 | 41.55 | 9.8 | 0.125 |
|  | 0.125 | 98.1 | 11.1 | 11.31 | -1.9 |  |
|  | 1.25 | 103.9 | 7.4 | 7.14 | 3.9 |  |
|  | 5 | 111.0 | 16.1 | 14.53 | 11.0 |  |
| 4-BROMOMETHAMPHETAMINE | 0.025 | 130.7 | 18.2 | 13.93 | 30.7 | 0.125 |
|  | 0.125 | 102.5 | 14.3 | 13.98 | 2.5 |  |
|  | 1.25 | 86.5 | 7.3 | 8.45 | -13.5 |  |
|  | 5 | 101.4 | 14.6 | 14.39 | 1.4 |  |
| 4-BROMOMETHCATHINONE (BREFEDRONE) | 0.025 | 138.7 | 55.3 | 39.84 | 38.7 | 0.125 |
|  | 0.125 | 85.6 | 15.9 | 18.63 | -14.4 |  |
|  | 1.25 | 103.9 | 11.1 | 10.69 | 3.9 |  |
|  | 5 | 119.8 | 11.8 | 9.83 | 19.8 |  |
| 4-CAB | 0.025 | 89.4 | 23.5 | 26.25 | -10.7 | 0.125 |
|  | 0.125 | 103.1 | 10.9 | 10.55 | 3.1 |  |
|  | 1.25 | 100.7 | 11.4 | 11.33 | 0.7 |  |
|  | 5 | 114.2 | 5.5 | 4.79 | 14.2 |  |
| 4-CEC | 0.025 | 66.3 | 34.2 | 51.48 | -33.7 | 0.125 |
|  | 0.125 | 97.2 | 19.4 | 19.92 | -2.8 |  |
|  | 1.25 | 93.9 | 3.4 | 3.64 | -6.1 |  |
|  | 5 | 97.9 | 8.2 | 8.37 | -2.1 |  |
| 4-CHLORO-ALPHA-PPP | 0.025 | 105.6 | 15.5 | 14.71 | 5.6 | 0.125 |
|  | 0.125 | 92.3 | 7.5 | 8.17 | -7.7 |  |
|  | 1.25 | 102.5 | 10.8 | 10.49 | 2.5 |  |
|  | 5 | 110.0 | 8.4 | 7.68 | 10.0 |  |
| 4-CHLORO-ALPHA-PVP | 0.025 | 100.0 | 16.1 | 16.08 | 0.0 | 0.125 |
|  | 0.125 | 97.8 | 9.4 | 9.59 | -2.2 |  |
|  | 1.25 | 81.8 | 7.8 | 9.50 | -18.2 |  |
|  | 5 | 96.3 | 4.3 | 4.45 | -3.7 |  |
| 4-CHLOROAMPHETAMINE | 0.025 | 103.5 | 39.1 | 37.75 | 3.5 | 0.125 |
|  | 0.125 | 104.0 | 7.4 | 7.08 | 4.0 |  |
|  | 1.25 | 80.7 | 2.7 | 3.33 | -19.3 |  |
|  | 5 | 110.1 | 18.7 | 17.00 | 10.1 |  |
| 4-CHLOROMETHCATHINONE (4-CMC) | 0.025 | 89.7 | 17.4 | 19.38 | -10.4 | 0.125 |
|  | 0.125 | 96.5 | 15.4 | 15.92 | -3.5 |  |
|  | 1.25 | 92.9 | 11.7 | 12.59 | -7.1 |  |
|  | 5 | 95.7 | 12.3 | 12.89 | -4.3 |  |
| 4-CMA | 0.025 | 123.6 | 10.8 | 8.77 | 23.6 | 0.125 |
|  | 0.125 | 102.9 | 11.6 | 11.31 | 2.9 |  |
|  | 1.25 | 102.8 | 12.8 | 12.49 | 2.8 |  |
|  | 5 | 107.5 | 13.2 | 12.27 | 7.5 |  |
| 4-EAPB | 0.025 | 85.1 | 5.9 | 6.95 | -14.9 | 0.125 |
|  | 0.125 | 91.3 | 6.7 | 7.30 | -8.7 |  |
|  | 1.25 | 97.4 | 11.9 | 12.18 | -2.6 |  |
|  | 5 | 100.1 | 8.2 | 8.19 | 0.1 |  |
| 4-ETHYL-N.N-DMC | 0.025 | 80.0 | 17.1 | 21.43 | -20.0 | 0.125 |
|  | 0.125 | 92.2 | 8.4 | 9.06 | -7.8 |  |
|  | 1.25 | 103.5 | 7.3 | 7.06 | 3.5 |  |
|  | 5 | 106.4 | 6.7 | 6.34 | 6.4 |  |
| 4-FEC | 0.025 | 100.3 | 15.4 | 15.32 | 0.3 | 0.125 |
|  | 0.125 | 104.8 | 10.3 | 9.84 | 4.8 |  |
|  | 1.25 | 90.3 | 4.1 | 4.57 | -9.7 |  |
|  | 5 | 89.8 | 4.8 | 5.30 | -10.2 |  |
| 4-FLUORO BUPHEDRONE | 0.025 | 81.6 | 14.6 | 17.90 | -18.4 | 0.125 |
|  | 0.125 | 106.4 | 10.4 | 9.82 | 6.4 |  |
|  | 1.25 | 102.8 | 10.2 | 9.90 | 2.8 |  |
|  | 5 | 107.4 | 10.2 | 9.52 | 7.4 |  |
| 4-FLUORO PENTEDRONE | 0.025 | 108.1 | 21.5 | 19.85 | 8.1 | 0.125 |
|  | 0.125 | 116.3 | 12.8 | 11.00 | 16.3 |  |
|  | 1.25 | 94.1 | 13.4 | 14.21 | -5.9 |  |
|  | 5 | 109.1 | 7.3 | 6.74 | 9.1 |  |
| 4-FLUORO PV8 | 0.025 | 108.5 | 6.5 | 5.97 | 8.5 | 0.125 |
|  | 0.125 | 97.7 | 7.1 | 7.26 | -2.3 |  |
|  | 1.25 | 98.2 | 8.5 | 8.69 | -1.8 |  |
|  | 5 | 110.3 | 7.0 | 6.36 | 10.3 |  |
| 4-FLUORO PV9 | 0.025 | 104.9 | 8.8 | 8.40 | 4.9 | 0.125 |
|  | 0.125 | 104.1 | 5.1 | 4.95 | 4.1 |  |
|  | 1.25 | 92.3 | 7.8 | 8.39 | -7.7 |  |
|  | 5 | 98.7 | 8.7 | 8.80 | -1.3 |  |
| 4-FLUORO-ALFA-PPP | 0.025 | 111.1 | 31.6 | 28.48 | 11.1 | 0.125 |
|  | 0.125 | 108.3 | 10.0 | 9.20 | 8.3 |  |
|  | 1.25 | 97.7 | 8.1 | 8.25 | -2.3 |  |
|  | 5 | 108.6 | 11.8 | 10.89 | 8.6 |  |
| 4-FLUOROMETHAMPHETAMINE (4-FMA) | 0.025 | 103.1 | 7.8 | 7.59 | 3.1 | 0.125 |
|  | 0.125 | 101.9 | 6.8 | 6.66 | 1.9 |  |
|  | 1.25 | 91.7 | 10.8 | 11.78 | -8.3 |  |
|  | 5 | 99.3 | 13.7 | 13.84 | -0.7 |  |
| 4-FLUOROMETHCATHINONE (4-FMC) | 0.025 | 107.3 | 10.3 | 9.65 | 7.3 | 0.125 |
|  | 0.125 | 97.5 | 13.5 | 13.86 | -2.5 |  |
|  | 1.25 | 99.9 | 4.3 | 4.28 | -0.1 |  |
|  | 5 | 102.5 | 5.9 | 5.76 | 2.5 |  |
| 4F-PVP | 0.025 | 112.5 | 12.3 | 10.97 | 12.5 | 0.125 |
|  | 0.125 | 105.7 | 7.2 | 6.79 | 5.7 |  |
|  | 1.25 | 101.8 | 10.4 | 10.18 | 1.8 |  |
|  | 5 | 115.8 | 7.7 | 6.64 | 15.8 |  |
| 4-HYDROXY DiPT | 0.025 | 97.9 | 28.9 | 29.56 | -2.1 | 0.125 |
|  | 0.125 | 99.1 | 15.5 | 15.66 | -0.9 |  |
|  | 1.25 | 102.6 | 15.3 | 14.94 | 2.6 |  |
|  | 5 | 113.4 | 8.7 | 7.68 | 13.4 |  |
| 4-HYDROXYMIDAZOLAM | 0.025 | 34.7 | 26.5 | 76.51 | -65.3 | 1.25 |
|  | 0.125 | 42.5 | 16.8 | 39.57 | -57.5 |  |
|  | 1.25 | 90.7 | 11.7 | 12.90 | -9.3 |  |
|  | 5 | 86.2 | 12.5 | 14.46 | -13.8 |  |
| 4-IODOAMPHETAMINE | 0.025 | 182.6 | 61.4 | 33.60 | 82.6 | 0.125 |
|  | 0.125 | 95.9 | 16.8 | 17.55 | -4.1 |  |
|  | 1.25 | 101.4 | 19.5 | 19.24 | 1.4 |  |
|  | 5 | 111.4 | 7.7 | 6.92 | 11.4 |  |
| 4-MAPB | 0.025 | 92.6 | 20.8 | 22.42 | -7.4 | 0.125 |
|  | 0.125 | 113.2 | 18.7 | 16.52 | 13.2 |  |
|  | 1.25 | 93.3 | 8.5 | 9.14 | -6.7 |  |
|  | 5 | 105.7 | 17.5 | 16.51 | 5.7 |  |
| 4-MEAP | 0.025 | 99.4 | 17.3 | 17.36 | -0.6 | 0.125 |
|  | 0.125 | 105.8 | 14.3 | 13.50 | 5.8 |  |
|  | 1.25 | 96.8 | 12.7 | 13.14 | -3.2 |  |
|  | 5 | 112.4 | 12.0 | 10.70 | 12.4 |  |
| 4-MEO-ALPHA-PVP | 0.025 | 74.8 | 23.2 | 30.97 | -25.2 | 0.125 |
|  | 0.125 | 99.7 | 7.8 | 7.82 | -0.3 |  |
|  | 1.25 | 118.5 | 17.3 | 14.61 | 18.5 |  |
|  | 5 | 111.0 | 9.3 | 8.36 | 11.0 |  |
| 4-MeOPBP | 0.025 | 120.9 | 11.0 | 9.10 | 20.9 | 0.125 |
|  | 0.125 | 93.5 | 9.7 | 10.32 | -6.5 |  |
|  | 1.25 | 105.0 | 6.4 | 6.10 | 5.0 |  |
|  | 5 | 111.9 | 6.9 | 6.14 | 11.9 |  |
| 4-METHOXY PHENCYCLIDINE | 0.025 | 98.5 | 11.7 | 11.88 | -1.5 | 0.125 |
|  | 0.125 | 103.0 | 12.3 | 11.92 | 3.0 |  |
|  | 1.25 | 112.4 | 6.6 | 5.90 | 12.4 |  |
|  | 5 | 107.5 | 6.7 | 6.26 | 7.5 |  |
| 4-METHOXY PV8 | 0.025 | 104.1 | 9.9 | 9.56 | 4.1 | 0.125 |
|  | 0.125 | 101.5 | 6.7 | 6.62 | 1.5 |  |
|  | 1.25 | 91.4 | 3.5 | 3.83 | -8.6 |  |
|  | 5 | 109.2 | 15.3 | 14.05 | 9.2 |  |
| 4-METHOXY PV9 | 0.025 | 99.2 | 11.5 | 11.57 | -0.8 | 0.125 |
|  | 0.125 | 101.2 | 7.9 | 7.80 | 1.2 |  |
|  | 1.25 | 87.4 | 8.8 | 10.08 | -12.6 |  |
|  | 5 | 110.7 | 17.4 | 15.73 | 10.7 |  |
| 4-METHOXYMETHAMPHETAMINE (PMMA) | 0.025 | 101.0 | 33.6 | 33.23 | 1.0 | 0.125 |
|  | 0.125 | 96.6 | 8.4 | 8.74 | -3.4 |  |
|  | 1.25 | 93.2 | 8.1 | 8.72 | -6.8 |  |
|  | 5 | 106.1 | 9.7 | 9.18 | 6.1 |  |
| 4-METHYL PENTEDRONE | 0.025 | 100.9 | 5.5 | 5.49 | 0.9 | 0.125 |
|  | 0.125 | 97.8 | 10.8 | 11.07 | -2.2 |  |
|  | 1.25 | 87.5 | 4.6 | 5.30 | -12.5 |  |
|  | 5 | 95.5 | 15.0 | 15.70 | -4.5 |  |
| 4-METHYL-ALPHA-ETHYLAMINOBUTIOPHENONE | 0.025 | 60.3 | 11.4 | 18.88 | -39.8 | 0.125 |
|  | 0.125 | 88.3 | 13.3 | 15.04 | -11.7 |  |
|  | 1.25 | 98.7 | 10.2 | 10.36 | -1.3 |  |
|  | 5 | 108.0 | 14.5 | 13.43 | 8.0 |  |
| 4-METHYLAMPHETAMINE | 0.025 | 103.0 | 49.3 | 47.92 | 3.0 | 0.125 |
|  | 0.125 | 91.2 | 5.4 | 5.91 | -8.8 |  |
|  | 1.25 | 105.1 | 14.2 | 13.54 | 5.1 |  |
|  | 5 | 118.4 | 16.7 | 14.10 | 18.4 |  |
| 4-METHYLCATHINONE | 0.025 | 88.5 | 29.8 | 33.72 | -11.5 | 0.125 |
|  | 0.125 | 93.0 | 6.6 | 7.07 | -7.0 |  |
|  | 1.25 | 115.2 | 17.2 | 14.93 | 15.2 |  |
|  | 5 | 105.0 | 8.7 | 8.32 | 5.0 |  |
| 4-METHYLMETHAMPHETAMINE (4-MMA) | 0.025 | 99.0 | 19.7 | 19.92 | -1.1 | 0.125 |
|  | 0.125 | 99.2 | 8.3 | 8.35 | -0.8 |  |
|  | 1.25 | 115.5 | 11.0 | 9.52 | 15.5 |  |
|  | 5 | 114.7 | 5.8 | 5.06 | 14.7 |  |
| 4-METHYLMETHCATHINONE (4-MMC) | 0.025 | 109.6 | 5.6 | 5.09 | 9.6 | 0.125 |
|  | 0.125 | 102.6 | 7.2 | 6.99 | 2.6 |  |
|  | 1.25 | 109.7 | 14.4 | 13.16 | 9.7 |  |
|  | 5 | 113.4 | 9.1 | 8.03 | 13.4 |  |
| 4-METHYL-N.N-DMC | 0.025 | 125.2 | 35.5 | 28.37 | 25.2 | 0.125 |
|  | 0.125 | 93.1 | 10.3 | 11.07 | -6.9 |  |
|  | 1.25 | 105.4 | 7.4 | 7.01 | 5.4 |  |
|  | 5 | 118.2 | 9.0 | 7.62 | 18.2 |  |
| 4-METHYL-N-METHYLBUPHEDRONE | 0.025 | 132.2 | 35.4 | 26.75 | 32.2 | 0.125 |
|  | 0.125 | 99.0 | 12.0 | 12.14 | -1.0 |  |
|  | 1.25 | 97.6 | 6.9 | 7.04 | -2.4 |  |
|  | 5 | 106.1 | 7.4 | 6.95 | 6.1 |  |
| 4-METHYL-N-METHYLHEXANOPHENONE | 0.025 | 82.4 | 13.4 | 16.27 | -17.6 | 0.125 |
|  | 0.125 | 90.6 | 10.7 | 11.82 | -9.4 |  |
|  | 1.25 | 90.7 | 16.8 | 18.47 | -9.3 |  |
|  | 5 | 98.0 | 11.0 | 11.20 | -2.0 |  |
| 4-METHYL-PBP | 0.025 | 106.4 | 18.2 | 17.14 | 6.4 | 0.125 |
|  | 0.125 | 107.5 | 7.4 | 6.91 | 7.5 |  |
|  | 1.25 | 117.3 | 13.6 | 11.63 | 17.3 |  |
|  | 5 | 113.8 | 6.9 | 6.07 | 13.8 |  |
| 4-METHYL-PHP | 0.025 | 106.1 | 14.0 | 13.16 | 6.1 | 0.125 |
|  | 0.125 | 101.1 | 2.5 | 2.49 | 1.1 |  |
|  | 1.25 | 97.6 | 9.6 | 9.80 | -2.4 |  |
|  | 5 | 109.5 | 7.1 | 6.49 | 9.5 |  |
| 4-METHYL-α-ETHYLTRYPTAMINE | 0.025 | 109.8 | 56.0 | 51.00 | 9.8 | 0.125 |
|  | 0.125 | 104.3 | 10.7 | 10.22 | 4.3 |  |
|  | 1.25 | 109.8 | 11.8 | 10.74 | 9.8 |  |
|  | 5 | 100.9 | 11.7 | 11.58 | 0.9 |  |
| 5-APB | 0.125 | 93.2 | 16.7 | 17.89 | -6.8 | 0.125 |
|  | 1.25 | 82.6 | 11.6 | 14.02 | -17.4 |  |
|  | 5 | 105.7 | 13.7 | 12.94 | 5.7 |  |
| 5-APDB | 0.125 | 112.4 | 21.9 | 19.46 | 12.4 | 0.125 |
|  | 1.25 | 93.1 | 10.0 | 10.78 | -6.9 |  |
|  | 5 | 93.6 | 13.4 | 14.37 | -6.4 |  |
| 5-APDI | 0.125 | 106.6 | 9.0 | 8.49 | 6.6 | 0.125 |
|  | 1.25 | 101.3 | 17.5 | 17.25 | 1.3 |  |
|  | 5 | 99.0 | 17.2 | 17.40 | -1.0 |  |
| 5-CHLORO AB-PINACA | 0.025 | 96.7 | 10.0 | 10.34 | -3.3 | 0.025 |
|  | 0.125 | 103.6 | 5.7 | 5.45 | 3.6 |  |
|  | 1.25 | 111.1 | 20.2 | 18.14 | 11.1 |  |
| 5-CHLORO-NNEI | 0.025 | 97.5 | 17.1 | 17.53 | -2.5 | 0.025 |
|  | 0.125 | 106.1 | 4.2 | 3.97 | 6.1 |  |
|  | 1.25 | 117.2 | 8.2 | 7.00 | 17.2 |  |
| 5-EAPB | 0.025 | 46.6 | 53.3 | 114.26 | -53.4 | 0.125 |
|  | 0.125 | 86.7 | 6.1 | 7.01 | -13.4 |  |
|  | 1.25 | 94.2 | 17.2 | 18.23 | -5.8 |  |
|  | 5 | 101.1 | 9.1 | 9.02 | 1.1 |  |
| 5F-ABICA | 0.025 | 106.2 | 6.6 | 6.21 | 6.2 | 0.025 |
|  | 0.125 | 107.3 | 9.2 | 8.58 | 7.3 |  |
|  | 1.25 | 95.7 | 10.6 | 11.03 | -4.3 |  |
| 5F-AB-PINACA | 0.025 | 118.3 | 8.1 | 6.86 | 18.3 | 0.025 |
|  | 0.125 | 104.3 | 9.1 | 8.75 | 4.3 |  |
|  | 1.25 | 110.8 | 3.4 | 3.04 | 10.8 |  |
| 5F-ADB (5F-MDMB-PINACA) | 0.025 | 87.3 | 15.3 | 17.50 | -12.7 | 0.025 |
|  | 0.125 | 106.4 | 6.8 | 6.38 | 6.4 |  |
|  | 1.25 | 104.3 | 7.6 | 7.29 | 4.3 |  |
| 5F-AMB (5F-AMB-PINACA) | 0.025 | 103.5 | 1.1 | 1.05 | 3.5 | 0.025 |
|  | 0.125 | 99.3 | 3.4 | 3.43 | -0.7 |  |
|  | 1.25 | 101.2 | 9.0 | 8.92 | 1.2 |  |
| 5F-APICA (STS-135) | 0.025 | 118.1 | 9.4 | 7.98 | 18.1 | 0.025 |
|  | 0.125 | 110.7 | 8.4 | 7.63 | 10.7 |  |
|  | 1.25 | 115.1 | 9.8 | 8.48 | 15.1 |  |
| 5F-APINACA (AKB-48-5F) | 0.025 | 115.2 | 9.2 | 8.02 | 15.2 | 0.025 |
|  | 0.125 | 116.2 | 14.2 | 12.23 | 16.2 |  |
|  | 1.25 | 114.3 | 20.3 | 17.75 | 14.3 |  |
| 5-F-JWH-018 ADAMANTYL ANALOG | 0.025 | 109.7 | 9.9 | 9.02 | 9.7 | 0.025 |
|  | 0.125 | 111.8 | 10.3 | 9.20 | 11.8 |  |
|  | 1.25 | 113.4 | 8.2 | 7.23 | 13.4 |  |
| 5-FLUORO MN-24 (5-FLUORO NNEI) | 0.025 | 111.3 | 7.0 | 6.33 | 11.3 | 0.025 |
|  | 0.125 | 106.7 | 4.2 | 3.93 | 6.7 |  |
|  | 1.25 | 98.8 | 8.5 | 8.60 | -1.2 |  |
| 5-FLUORO THJ | 0.025 | 99.4 | 8.3 | 8.34 | -0.6 | 0.025 |
|  | 0.125 | 102.6 | 15.2 | 14.81 | 2.6 |  |
|  | 1.25 | 116.3 | 17.1 | 14.67 | 16.3 |  |
| 5-FLUORO-2-ADB-PINACA_ISOMER_2 | 0.025 | 108.9 | 15.9 | 14.62 | 8.9 | 0.025 |
|  | 0.125 | 110.7 | 9.4 | 8.48 | 10.7 |  |
|  | 1.25 | 106.9 | 8.3 | 7.77 | 6.9 |  |
| 5-FLUORO-CUMYL-PICA | 0.025 | 112.0 | 10.1 | 8.99 | 12.0 | 0.025 |
|  | 0.125 | 114.1 | 5.1 | 4.43 | 14.1 |  |
|  | 1.25 | 102.4 | 11.5 | 11.27 | 2.4 |  |
| 5-FLUORO-MN-18 | 0.025 | 93.9 | 8.1 | 8.66 | -6.1 | 0.025 |
|  | 0.125 | 99.3 | 3.9 | 3.97 | -0.7 |  |
|  | 1.25 | 113.7 | 15.4 | 13.51 | 13.7 |  |
| 5-FLUORO-MN-21 (5-FLUORO-PCN) | 0.025 | 87.8 | 9.9 | 11.33 | -12.3 | 0.025 |
|  | 0.125 | 92.0 | 3.1 | 3.42 | -8.0 |  |
|  | 1.25 | 85.2 | 14.9 | 17.47 | -14.8 |  |
| 5-FLUORO-SDB-006 | 0.025 | 113.1 | 6.4 | 5.70 | 13.1 | 0.025 |
|  | 0.125 | 101.5 | 6.0 | 5.96 | 1.5 |  |
|  | 1.25 | 110.9 | 8.3 | 7.49 | 10.9 |  |
| 5F-NPB-22 | 0.025 | 90.1 | 14.5 | 16.09 | -9.9 | 0.025 |
|  | 0.125 | 116.8 | 13.9 | 11.88 | 16.8 |  |
|  | 1.25 | 113.7 | 12.0 | 10.56 | 13.7 |  |
| 5F-PB-22 | 0.025 | 106.9 | 5.1 | 4.79 | 6.9 | 0.025 |
|  | 0.125 | 113.7 | 9.9 | 8.74 | 13.7 |  |
|  | 1.25 | 91.8 | 11.2 | 12.17 | -8.2 |  |
| 5-F-PENTYL-3-PYRIDINOYLINDOLE | 0.025 | 97.1 | 11.6 | 11.95 | -2.9 | 0.025 |
|  | 0.125 | 107.4 | 6.7 | 6.26 | 7.4 |  |
|  | 1.25 | 93.1 | 10.9 | 11.72 | -6.9 |  |
| 5F-SDB-005 | 0.025 | 87.5 | 11.2 | 12.80 | -12.5 | 0.025 |
|  | 0.125 | 112.9 | 4.6 | 4.09 | 12.9 |  |
|  | 1.25 | 113.6 | 10.7 | 9.42 | 13.6 |  |
| 5-HYDROXY DMT | 0.025 | 174.4 | 24.9 | 14.28 | 74.4 | 0.125 |
|  | 0.125 | 113.2 | 7.0 | 6.21 | 13.2 |  |
|  | 1.25 | 109.2 | 4.3 | 3.93 | 9.2 |  |
|  | 5 | 115.2 | 4.7 | 4.09 | 15.2 |  |
| 5-IT | 0.025 | 106.6 | 66.3 | 62.18 | 6.6 | 0.125 |
|  | 0.125 | 82.9 | 13.3 | 16.09 | -17.1 |  |
|  | 1.25 | 98.2 | 11.3 | 11.52 | -1.8 |  |
|  | 5 | 102.7 | 10.3 | 10.06 | 2.7 |  |
| 5-MAPB | 0.025 | 114.3 | 9.9 | 8.65 | 14.3 | 0.125 |
|  | 0.125 | 96.2 | 10.0 | 10.37 | -3.8 |  |
|  | 1.25 | 108.0 | 10.7 | 9.90 | 8.0 |  |
|  | 5 | 119.6 | 14.0 | 11.72 | 19.6 |  |
| 5-MAPDB | 0.025 | 106.8 | 9.7 | 9.07 | 6.8 | 0.125 |
|  | 0.125 | 93.3 | 9.4 | 10.05 | -6.7 |  |
|  | 1.25 | 88.6 | 6.5 | 7.31 | -11.4 |  |
|  | 5 | 95.7 | 10.8 | 11.26 | -4.3 |  |
| 5-MeO-ALPHA-ET | 0.025 | 65.1 | 27.7 | 42.57 | -34.9 | 0.125 |
|  | 0.125 | 97.6 | 18.6 | 19.06 | -2.4 |  |
|  | 1.25 | 119.6 | 9.3 | 7.80 | 19.6 |  |
|  | 5 | 114.3 | 8.8 | 7.73 | 14.3 |  |
| 5-MeO-DALT | 0.025 | 98.1 | 10.2 | 10.41 | -1.9 | 0.125 |
|  | 0.125 | 98.9 | 7.3 | 7.42 | -1.1 |  |
|  | 1.25 | 100.3 | 8.1 | 8.04 | 0.3 |  |
|  | 5 | 114.6 | 11.2 | 9.80 | 14.6 |  |
| 5-METHOXY AMT | 0.025 | 110.3 | 35.3 | 31.99 | 10.3 | 0.125 |
|  | 0.125 | 98.8 | 12.8 | 12.97 | -1.2 |  |
|  | 1.25 | 108.4 | 12.6 | 11.67 | 8.4 |  |
|  | 5 | 109.9 | 16.8 | 15.28 | 9.9 |  |
| 5-METHOXY DiPT | 0.025 | 120.2 | 16.5 | 13.70 | 20.2 | 0.125 |
|  | 0.125 | 97.3 | 6.7 | 6.86 | -2.7 |  |
|  | 1.25 | 98.2 | 9.6 | 9.80 | -1.8 |  |
|  | 5 | 97.7 | 9.0 | 9.22 | -2.3 |  |
| 5-METHOXY DMT | 0.125 | 118.7 | 9.3 | 7.80 | 18.7 | 0.125 |
|  | 1.25 | 91.9 | 10.0 | 10.88 | -8.1 |  |
|  | 5 | 115.9 | 21.5 | 18.52 | 15.9 |  |
| 5-METHOXY METHYLONE | 0.025 | 69.1 | 12.2 | 17.68 | -30.9 | 0.125 |
|  | 0.125 | 105.5 | 6.4 | 6.08 | 5.5 |  |
|  | 1.25 | 94.7 | 14.2 | 14.95 | -5.3 |  |
|  | 5 | 99.7 | 9.3 | 9.29 | -0.3 |  |
| 5-METHOXY MiPT | 0.125 | 96.5 | 16.6 | 17.20 | -3.5 | 0.125 |
|  | 1.25 | 104.9 | 19.9 | 18.97 | 4.9 |  |
|  | 5 | 118.4 | 14.8 | 12.53 | 18.4 |  |
| 6-APB | 0.125 | 97.2 | 17.1 | 17.57 | -2.8 | 0.125 |
|  | 1.25 | 98.0 | 9.7 | 9.86 | -2.0 |  |
|  | 5 | 112.5 | 6.8 | 6.04 | 12.5 |  |
| 6-APDB | 0.025 | 185.7 | 139.0 | 74.85 | 85.7 | 0.125 |
|  | 0.125 | 107.9 | 17.5 | 16.20 | 7.9 |  |
|  | 1.25 | 94.0 | 16.1 | 17.17 | -6.0 |  |
|  | 5 | 117.4 | 3.9 | 3.28 | 17.4 |  |
| 6-BROMO-MDMA | 0.025 | 109.4 | 9.9 | 9.10 | 9.4 | 0.125 |
|  | 0.125 | 110.0 | 5.9 | 5.38 | 10.0 |  |
|  | 1.25 | 92.3 | 16.4 | 17.73 | -7.7 |  |
|  | 5 | 102.2 | 17.7 | 17.32 | 2.2 |  |
| 6-CHLORO-MDMA | 0.025 | 94.8 | 15.1 | 15.91 | -5.2 | 0.125 |
|  | 0.125 | 105.8 | 11.4 | 10.77 | 5.8 |  |
|  | 1.25 | 97.3 | 8.7 | 8.96 | -2.7 |  |
|  | 5 | 101.5 | 10.8 | 10.69 | 1.5 |  |
| 6-EAPB | 0.025 | 89.2 | 19.0 | 21.29 | -10.8 | 0.125 |
|  | 0.125 | 104.9 | 9.6 | 9.13 | 4.9 |  |
|  | 1.25 | 102.2 | 8.6 | 8.37 | 2.2 |  |
|  | 5 | 109.6 | 9.6 | 8.79 | 9.6 |  |
| 6-IT | 0.025 | 157.1 | 62.6 | 39.85 | 57.1 | 0.125 |
|  | 0.125 | 110.1 | 6.6 | 6.04 | 10.1 |  |
|  | 1.25 | 102.8 | 4.8 | 4.66 | 2.8 |  |
|  | 5 | 105.4 | 8.0 | 7.61 | 5.4 |  |
| 6-MAM | 0.025 | 136.6 | 23.3 | 17.04 | 36.6 | 0.125 |
|  | 0.125 | 89.0 | 11.4 | 12.81 | -11.0 |  |
|  | 1.25 | 96.6 | 7.1 | 7.39 | -3.4 |  |
|  | 5 | 104.0 | 10.9 | 10.50 | 4.0 |  |
| 6-MAPB | 0.025 | 109.6 | 11.9 | 10.84 | 9.6 | 0.125 |
|  | 0.125 | 93.1 | 11.4 | 12.27 | -7.0 |  |
|  | 1.25 | 99.7 | 8.5 | 8.52 | -0.3 |  |
|  | 5 | 107.0 | 10.1 | 9.47 | 7.0 |  |
| 7-AMINOCLONAZEPAM | 0.025 | 83.3 | 44.3 | 53.18 | -16.7 | 0.125 |
|  | 0.125 | 92.5 | 11.8 | 12.81 | -7.6 |  |
|  | 1.25 | 101.5 | 9.1 | 8.95 | 1.5 |  |
|  | 5 | 96.9 | 7.2 | 7.42 | -3.1 |  |
| 7-AMINODESMETHYLFLUNITRAZEPAM | 0.025 | 100.3 | 13.7 | 13.70 | 0.3 | 0.125 |
|  | 0.125 | 88.1 | 7.1 | 8.06 | -11.9 |  |
|  | 1.25 | 88.0 | 13.7 | 15.60 | -12.0 |  |
|  | 5 | 89.9 | 5.4 | 5.96 | -10.1 |  |
| 7-AMINOFLUNITRAZEPAM | 0.025 | 89.6 | 25.8 | 28.75 | -10.4 | 0.125 |
|  | 0.125 | 92.1 | 13.8 | 15.00 | -7.9 |  |
|  | 1.25 | 92.0 | 11.4 | 12.40 | -8.0 |  |
|  | 5 | 119.8 | 13.5 | 11.25 | 19.8 |  |
| 7-AMINONITRAZEPAM | 0.025 | 117.3 | 31.3 | 26.70 | 17.3 | 0.125 |
|  | 0.125 | 88.6 | 11.5 | 12.99 | -11.4 |  |
|  | 1.25 | 91.4 | 7.7 | 8.47 | -8.6 |  |
|  | 5 | 104.2 | 3.4 | 3.30 | 4.2 |  |
| 7-APB | 0.025 | 117.7 | 26.6 | 22.62 | 17.7 | 0.125 |
|  | 0.125 | 104.1 | 16.0 | 15.34 | 4.1 |  |
|  | 1.25 | 88.1 | 9.4 | 10.68 | -11.9 |  |
|  | 5 | 107.7 | 13.4 | 12.42 | 7.7 |  |
| 7-APDB | 0.025 | 258.0 | 163.1 | 63.20 | 158.0 | 0.125 |
|  | 0.125 | 104.9 | 17.6 | 16.80 | 4.9 |  |
|  | 1.25 | 110.0 | 17.5 | 15.95 | 10.0 |  |
|  | 5 | 112.0 | 15.1 | 13.48 | 12.0 |  |
| A-796260 | 0.025 | 106.8 | 12.7 | 11.86 | 6.8 | 0.025 |
|  | 0.125 | 101.1 | 5.3 | 5.27 | 1.1 |  |
|  | 1.25 | 86.8 | 9.5 | 10.97 | -13.2 |  |
| A-834735 | 0.025 | 97.5 | 10.9 | 11.15 | -2.5 | 0.025 |
|  | 0.125 | 106.2 | 8.3 | 7.78 | 6.2 |  |
|  | 1.25 | 113.5 | 5.4 | 4.73 | 13.5 |  |
| A-836339 | 0.025 | 102.9 | 11.8 | 11.46 | 2.9 | 0.025 |
|  | 0.125 | 114.4 | 7.9 | 6.89 | 14.4 |  |
|  | 1.25 | 97.0 | 9.3 | 9.61 | -3.0 |  |
| AB-001 | 0.025 | 111.0 | 12.8 | 11.58 | 11.0 | 0.025 |
|  | 0.125 | 114.2 | 4.2 | 3.68 | 14.2 |  |
|  | 1.25 | 118.4 | 6.0 | 5.11 | 18.4 |  |
| AB005 | 0.025 | 101.6 | 13.7 | 13.50 | 1.6 | 0.025 |
|  | 0.125 | 103.4 | 12.0 | 11.60 | 3.4 |  |
|  | 1.25 | 87.2 | 10.4 | 11.92 | -12.8 |  |
| AB-CHMINACA | 0.025 | 110.7 | 9.3 | 8.44 | 10.7 | 0.025 |
|  | 0.125 | 111.4 | 4.3 | 3.86 | 11.4 |  |
|  | 1.25 | 113.8 | 9.1 | 7.97 | 13.8 |  |
| AB-FUBINACA | 0.025 | 141.1 | 51.2 | 36.28 | 41.1 | 0.125 |
|  | 0.125 | 104.9 | 15.5 | 14.75 | 4.9 |  |
|  | 1.25 | 105.5 | 7.5 | 7.08 | 5.5 |  |
| AB-FUBINACA 2-FLUOROBENZYL ISOMER | 0.025 | 104.9 | 7.0 | 6.69 | 4.9 | 0.025 |
|  | 0.125 | 110.3 | 7.9 | 7.15 | 10.3 |  |
|  | 1.25 | 104.4 | 11.9 | 11.38 | 4.4 |  |
| AB-FUBINACA 3-FLUOROBENZYL ISOMER | 0.025 | 99.8 | 11.0 | 11.00 | -0.2 | 0.025 |
|  | 0.125 | 107.7 | 9.5 | 8.84 | 7.7 |  |
|  | 1.25 | 99.5 | 12.8 | 12.88 | -0.5 |  |
| AB-PINACA | 0.025 | 100.5 | 8.1 | 8.06 | 0.5 | 0.025 |
|  | 0.125 | 114.4 | 11.1 | 9.70 | 14.4 |  |
|  | 1.25 | 110.2 | 7.5 | 6.78 | 10.2 |  |
| ADB-FUBINACA | 0.025 | 111.1 | 12.2 | 10.94 | 11.1 | 0.025 |
|  | 0.125 | 112.4 | 11.6 | 10.35 | 12.4 |  |
|  | 1.25 | 107.3 | 4.8 | 4.45 | 7.3 |  |
| ADB-PINACA | 0.025 | 107.8 | 5.1 | 4.75 | 7.8 | 0.025 |
|  | 0.125 | 107.2 | 6.4 | 5.93 | 7.2 |  |
|  | 1.25 | 100.5 | 15.2 | 15.17 | 0.5 |  |
| ADB-PINACA ISOMER_1 | 0.025 | 107.4 | 13.2 | 12.25 | 7.4 | 0.025 |
|  | 0.125 | 101.5 | 19.8 | 19.48 | 1.5 |  |
|  | 1.25 | 102.4 | 10.8 | 10.59 | 2.4 |  |
| ADB-PINACA ISOMER_2 | 0.025 | 109.4 | 13.9 | 12.67 | 9.4 | 0.025 |
|  | 0.125 | 99.4 | 16.4 | 16.49 | -0.6 |  |
|  | 1.25 | 103.4 | 12.8 | 12.40 | 3.4 |  |
| ADB-PINACA ISOMER_3 | 0.025 | 88.3 | 7.1 | 8.00 | -11.8 | 0.025 |
|  | 0.125 | 99.6 | 13.5 | 13.56 | -0.4 |  |
|  | 1.25 | 100.0 | 5.3 | 5.29 | 0.0 |  |
| ADB-PINACA ISOMER_4 | 0.025 | 101.3 | 6.5 | 6.46 | 1.3 | 0.025 |
|  | 0.125 | 105.3 | 5.8 | 5.52 | 5.3 |  |
|  | 1.25 | 94.8 | 9.0 | 9.44 | -5.2 |  |
| AH-7921 | 0.025 | 91.1 | 10.7 | 11.77 | -8.9 | 0.125 |
|  | 0.125 | 94.3 | 8.4 | 8.96 | -5.7 |  |
|  | 1.25 | 90.4 | 4.5 | 4.94 | -9.6 |  |
|  | 5 | 103.4 | 14.4 | 13.97 | 3.4 |  |
| AKB48_N-(4-FLUOROBENZYL)_ANALOG | 0.025 | 96.9 | 16.9 | 17.41 | -3.1 | 0.025 |
|  | 0.125 | 111.7 | 9.0 | 8.07 | 11.7 |  |
|  | 1.25 | 181.7 | 22.9 | 12.58 | 81.7 |  |
| ALFA-ETHYLAMINOPENTIOPHENONE | 0.025 | 86.5 | 36.8 | 42.52 | -13.5 | 0.125 |
|  | 0.125 | 104.5 | 9.1 | 8.69 | 4.5 |  |
|  | 1.25 | 101.0 | 6.3 | 6.29 | 1.0 |  |
|  | 5 | 102.7 | 6.7 | 6.54 | 2.7 |  |
| ALFA-PROPYLAMINOPENTIOPHENONE | 0.025 | 109.8 | 11.3 | 10.29 | 9.8 | 0.125 |
|  | 0.125 | 102.5 | 11.6 | 11.27 | 2.5 |  |
|  | 1.25 | 116.0 | 6.6 | 5.68 | 16.0 |  |
|  | 5 | 117.7 | 13.2 | 11.25 | 17.7 |  |
| ALLYLESCALINE | 0.125 | 101.0 | 20.0 | 19.80 | 1.0 | 0.125 |
|  | 1.25 | 99.1 | 9.9 | 10.00 | -0.9 |  |
|  | 5 | 116.4 | 8.9 | 7.68 | 16.4 |  |
| ALPHA-DIMETHYLAMINOPENTIOPHENONE | 0.025 | 86.6 | 18.8 | 21.71 | -13.4 | 0.125 |
|  | 0.125 | 98.0 | 11.6 | 11.86 | -2.0 |  |
|  | 1.25 | 95.7 | 6.7 | 7.02 | -4.3 |  |
|  | 5 | 110.1 | 16.3 | 14.78 | 10.1 |  |
| ALPHA-ETHYLAMINOHEXANOPHENONE | 0.025 | 91.5 | 36.0 | 39.31 | -8.5 | 0.125 |
|  | 0.125 | 116.6 | 14.9 | 12.75 | 16.6 |  |
|  | 1.25 | 104.6 | 15.6 | 14.90 | 4.6 |  |
|  | 5 | 95.9 | 14.3 | 14.91 | -4.1 |  |
| ALPHA-ETHYLTRYPTAMINE | 0.025 | 105.7 | 44.2 | 41.84 | 5.7 | 0.125 |
|  | 0.125 | 95.6 | 19.1 | 19.95 | -4.4 |  |
|  | 1.25 | 116.8 | 15.6 | 13.37 | 16.8 |  |
|  | 5 | 117.3 | 17.2 | 14.65 | 17.3 |  |
| ALPHA-METHYLTRYPTAMINE | 1.25 | 114.5 | 9.1 | 7.99 | 14.5 | 1.25 |
|  | 5 | 119.0 | 16.2 | 13.59 | 19.0 |  |
| ALPHA-PHP | 0.025 | 101.8 | 15.9 | 15.63 | 1.7 | 0.125 |
|  | 0.125 | 119.5 | 9.3 | 7.78 | 19.5 |  |
|  | 1.25 | 105.4 | 9.2 | 8.74 | 5.4 |  |
|  | 5 | 107.0 | 11.1 | 10.39 | 7.0 |  |
| ALPHA-PHTALIMIDOPROPIOPHENONE | 0.025 | 102.3 | 20.9 | 20.46 | 2.3 | 0.125 |
|  | 0.125 | 118.5 | 4.9 | 4.14 | 18.5 |  |
|  | 1.25 | 105.0 | 9.8 | 9.30 | 5.0 |  |
|  | 5 | 112.9 | 14.9 | 13.20 | 12.9 |  |
| ALPHA-PIPBP | 0.025 | 118.1 | 22.4 | 18.97 | 18.1 | 0.125 |
|  | 0.125 | 104.1 | 11.1 | 10.70 | 4.1 |  |
|  | 1.25 | 101.1 | 4.0 | 4.01 | 1.1 |  |
|  | 5 | 108.5 | 11.6 | 10.67 | 8.5 |  |
| ALPHA-PPP | 0.025 | 109.9 | 14.9 | 13.51 | 9.9 | 0.125 |
|  | 0.125 | 95.3 | 12.1 | 12.66 | -4.8 |  |
|  | 1.25 | 93.9 | 7.5 | 7.98 | -6.1 |  |
|  | 5 | 98.4 | 5.1 | 5.17 | -1.6 |  |
| ALPHA-PVP | 0.025 | 122.7 | 17.8 | 14.53 | 22.7 | 0.125 |
|  | 0.125 | 97.5 | 11.1 | 11.34 | -2.5 |  |
|  | 1.25 | 90.8 | 9.4 | 10.32 | -9.2 |  |
|  | 5 | 90.0 | 10.7 | 11.91 | -10.0 |  |
| ALPHA-PVT | 0.025 | 112.6 | 20.3 | 18.04 | 12.6 | 0.125 |
|  | 0.125 | 110.9 | 6.3 | 5.71 | 10.9 |  |
|  | 1.25 | 93.5 | 14.0 | 14.95 | -6.5 |  |
|  | 5 | 116.1 | 15.5 | 13.36 | 16.1 |  |
| ALPHA-PYRROLIDINOBUTHIOPHENONE | 0.025 | 102.9 | 11.5 | 11.17 | 2.9 | 0.125 |
|  | 0.125 | 94.7 | 10.6 | 11.22 | -5.3 |  |
|  | 1.25 | 98.0 | 4.8 | 4.92 | -2.0 |  |
|  | 5 | 105.9 | 11.9 | 11.23 | 5.9 |  |
| ALPRAZOLAM | 0.025 | 105.3 | 14.9 | 14.14 | 5.3 | 0.125 |
|  | 0.125 | 87.7 | 7.0 | 8.03 | -12.3 |  |
|  | 1.25 | 94.2 | 11.7 | 12.37 | -5.8 |  |
|  | 5 | 119.8 | 12.1 | 10.07 | 19.8 |  |
| AM1220 | 0.025 | 98.7 | 15.1 | 15.34 | -1.3 | 0.025 |
|  | 0.125 | 94.9 | 8.7 | 9.16 | -5.1 |  |
|  | 1.25 | 92.5 | 6.1 | 6.61 | -7.5 |  |
| AM-1248 | 0.025 | 99.1 | 12.5 | 12.65 | -1.0 | 0.025 |
|  | 0.125 | 112.4 | 4.0 | 3.59 | 12.4 |  |
|  | 1.25 | 112.2 | 9.8 | 8.70 | 12.2 |  |
| AM-2201 | 0.025 | 116.4 | 7.9 | 6.75 | 16.4 | 0.025 |
|  | 0.125 | 110.1 | 3.8 | 3.43 | 10.1 |  |
|  | 1.25 | 108.4 | 10.2 | 9.42 | 8.4 |  |
| AM2201 8-QUINOLINYL CARBOXAMIDE | 0.025 | 96.9 | 15.8 | 16.31 | -3.1 | 0.025 |
|  | 0.125 | 94.8 | 10.9 | 11.49 | -5.2 |  |
|  | 1.25 | 116.4 | 19.3 | 16.54 | 16.4 |  |
| AM2201 BENZIMIDAZOLE ANALOG | 0.025 | 105.6 | 12.2 | 11.56 | 5.6 | 0.025 |
|  | 0.125 | 108.6 | 6.0 | 5.57 | 8.6 |  |
|  | 1.25 | 109.3 | 9.7 | 8.88 | 9.3 |  |
| AM2232 | 0.025 | 111.1 | 9.1 | 8.20 | 11.1 | 0.025 |
|  | 0.125 | 100.4 | 7.9 | 7.88 | 0.4 |  |
|  | 1.25 | 103.4 | 10.1 | 9.79 | 3.4 |  |
| AMITRIPTYLINE | 0.025 | 103.1 | 18.6 | 18.04 | 3.1 | 0.125 |
|  | 0.125 | 102.8 | 7.6 | 7.43 | 2.8 |  |
|  | 1.25 | 92.3 | 8.4 | 9.06 | -7.7 |  |
|  | 5 | 105.3 | 10.6 | 10.05 | 5.3 |  |
| AMPHETAMINE | 0.025 | 101.1 | 14.9 | 14.75 | 1.1 | 0.125 |
|  | 0.125 | 113.1 | 16.5 | 14.62 | 13.1 |  |
|  | 1.25 | 89.7 | 11.6 | 12.93 | -10.3 |  |
|  | 5 | 104.3 | 9.2 | 8.82 | 4.3 |  |
| ATENOLOL | 0.025 | 152.5 | 32.5 | 21.32 | 52.5 | 0.125 |
|  | 0.125 | 119.9 | 13.0 | 10.86 | 19.9 |  |
|  | 1.25 | 114.4 | 12.0 | 10.48 | 14.4 |  |
|  | 5 | 120.0 | 13.4 | 11.20 | 20.0 |  |
| AZACYCLONOL | 0.025 | 138.0 | 12.6 | 9.15 | 38.0 | 0.125 |
|  | 0.125 | 103.6 | 16.8 | 16.20 | 3.6 |  |
|  | 1.25 | 111.8 | 12.2 | 10.92 | 11.8 |  |
|  | 5 | 93.0 | 10.4 | 11.13 | -7.0 |  |
| BB-22 | 0.025 | 101.0 | 11.7 | 11.60 | 1.0 | 0.025 |
|  | 0.125 | 112.1 | 8.0 | 7.15 | 12.1 |  |
|  | 1.25 | 107.6 | 6.4 | 5.91 | 7.6 |  |
| BDB | 0.125 | 100.1 | 14.1 | 14.12 | 0.1 | 0.125 |
|  | 1.25 | 104.0 | 11.1 | 10.64 | 4.0 |  |
|  | 5 | 102.9 | 17.4 | 16.94 | 2.9 |  |
| BENOCYCLIDINE | 0.025 | 109.7 | 10.7 | 9.78 | 9.7 | 0.125 |
|  | 0.125 | 99.7 | 9.4 | 9.46 | -0.3 |  |
|  | 1.25 | 94.7 | 10.7 | 11.34 | -5.3 |  |
|  | 5 | 110.5 | 8.5 | 7.73 | 10.5 |  |
| BENZEDRONE | 0.025 | 106.2 | 9.5 | 8.96 | 6.2 | 0.125 |
|  | 0.125 | 96.0 | 5.8 | 6.09 | -4.0 |  |
|  | 1.25 | 94.0 | 7.0 | 7.45 | -6.0 |  |
|  | 5 | 108.8 | 14.3 | 13.14 | 8.8 |  |
| BENZOCAINE | 0.025 | 129.4 | 72.2 | 55.78 | 29.4 | 0.125 |
|  | 0.125 | 102.1 | 10.0 | 9.76 | 2.1 |  |
|  | 1.25 | 109.5 | 18.5 | 16.93 | 9.5 |  |
|  | 5 | 117.3 | 9.2 | 7.85 | 17.3 |  |
| BENZOYLECGONINE | 0.025 | 107.8 | 17.6 | 16.32 | 7.8 | 0.025 |
|  | 0.125 | 101.3 | 13.8 | 13.65 | 1.3 |  |
|  | 1.25 | 89.4 | 9.3 | 10.45 | -10.6 |  |
|  | 5 | 109.4 | 19.3 | 17.61 | 9.4 |  |
| BENZYDAMINE | 0.025 | 101.2 | 9.3 | 9.20 | 1.2 | 0.125 |
|  | 0.125 | 93.5 | 5.4 | 5.79 | -6.5 |  |
|  | 1.25 | 93.8 | 8.3 | 8.80 | -6.2 |  |
|  | 5 | 99.0 | 10.6 | 10.70 | -1.0 |  |
| bk-MDA | 0.025 | 117.5 | 6.4 | 5.42 | 17.5 | 0.125 |
|  | 0.125 | 101.2 | 5.3 | 5.20 | 1.2 |  |
|  | 1.25 | 97.4 | 12.2 | 12.51 | -2.6 |  |
|  | 5 | 112.1 | 9.9 | 8.80 | 12.1 |  |
| BMPEA | 0.025 | 171.2 | 60.0 | 35.05 | 71.2 | 0.125 |
|  | 0.125 | 114.1 | 20.4 | 17.88 | 14.1 |  |
|  | 1.25 | 113.9 | 11.1 | 9.72 | 13.9 |  |
|  | 5 | 105.2 | 10.5 | 10.01 | 5.2 |  |
| BROMO-DRAGON-FLY | 0.025 | 83.5 | 15.7 | 18.81 | -16.5 | 0.125 |
|  | 0.125 | 95.6 | 17.4 | 18.24 | -4.4 |  |
|  | 1.25 | 84.9 | 10.0 | 11.77 | -15.1 |  |
|  | 5 | 106.4 | 10.8 | 10.12 | 6.4 |  |
| BUPRENORPHINE | 0.025 | 94.8 | 23.6 | 24.88 | -5.2 | 0.125 |
|  | 0.125 | 106.3 | 9.5 | 8.97 | 6.3 |  |
|  | 1.25 | 80.3 | 7.2 | 8.92 | -19.7 |  |
|  | 5 | 98.3 | 13.4 | 13.62 | -1.7 |  |
| BUPROPION | 0.025 | 117.4 | 37.7 | 32.09 | 17.4 | 0.125 |
|  | 0.125 | 113.2 | 16.8 | 14.85 | 13.2 |  |
|  | 1.25 | 107.8 | 4.2 | 3.94 | 7.8 |  |
|  | 5 | 112.0 | 2.4 | 2.15 | 12.0 |  |
| BUTYLONE (bk-MBDB) | 0.025 | 105.7 | 23.5 | 22.21 | 5.7 | 0.125 |
|  | 0.125 | 101.1 | 13.8 | 13.62 | 1.1 |  |
|  | 1.25 | 98.5 | 9.2 | 9.39 | -1.5 |  |
|  | 5 | 115.2 | 19.8 | 17.20 | 15.2 |  |
| CAMFETAMINE | 0.025 | 106.1 | 17.9 | 16.86 | 6.1 | 0.125 |
|  | 0.125 | 102.1 | 19.9 | 19.54 | 2.1 |  |
|  | 1.25 | 116.8 | 22.0 | 18.80 | 16.8 |  |
|  | 5 | 108.6 | 9.3 | 8.53 | 8.6 |  |
| CANNABIDIOL | 0.025 | 40.9 | 13.8 | 33.78 | -59.1 | 0.125 |
|  | 0.125 | 95.8 | 14.1 | 14.76 | -4.2 |  |
|  | 1.25 | 115.3 | 10.9 | 9.42 | 15.3 |  |
| CARBAMAZEPINE | 0.025 | 97.1 | 6.8 | 6.98 | -2.9 | 0.125 |
|  | 0.125 | 110.2 | 5.3 | 4.77 | 10.2 |  |
|  | 1.25 | 103.2 | 15.7 | 15.22 | 3.2 |  |
|  | 5 | 103.1 | 8.6 | 8.31 | 3.1 |  |
| CATHINE | 0.125 | 98.4 | 11.5 | 11.64 | -1.6 | 0.125 |
|  | 1.25 | 105.6 | 6.2 | 5.88 | 5.6 |  |
|  | 5 | 110.9 | 6.7 | 6.01 | 10.9 |  |
| CATHINONE | 0.025 | 142.9 | 30.4 | 21.26 | 42.9 | 0.125 |
|  | 0.125 | 108.6 | 4.9 | 4.51 | 8.6 |  |
|  | 1.25 | 102.3 | 11.6 | 11.31 | 2.3 |  |
|  | 5 | 108.8 | 6.0 | 5.53 | 8.8 |  |
| CB-13 | 0.025 | 106.0 | 5.4 | 5.13 | 6.0 | 0.025 |
|  | 0.125 | 109.6 | 4.5 | 4.08 | 9.6 |  |
|  | 1.25 | 118.2 | 7.3 | 6.20 | 18.2 |  |
| CBL-018 | 0.025 | 104.2 | 14.3 | 13.72 | 4.1 | 0.025 |
|  | 0.125 | 97.3 | 4.8 | 4.91 | -2.7 |  |
|  | 1.25 | 110.1 | 9.5 | 8.61 | 10.1 |  |
| CI2201 | 0.025 | 122.6 | 10.1 | 8.28 | 22.6 | 0.125 |
|  | 0.125 | 114.0 | 5.6 | 4.88 | 14.0 |  |
|  | 1.25 | 117.8 | 13.0 | 11.02 | 17.8 |  |
|  | 5 | 155.2 | 25.9 | 16.69 | 55.2 |  |
| CLOBAZAM | 0.025 | 131.3 | 14.3 | 10.93 | 31.3 | 0.125 |
|  | 0.125 | 105.6 | 15.2 | 14.42 | 5.6 |  |
|  | 1.25 | 108.7 | 6.3 | 5.80 | 8.7 |  |
|  | 5 | 113.7 | 15.0 | 13.23 | 13.7 |  |
| CLOMIPRAMINE | 0.025 | 111.5 | 8.1 | 7.24 | 11.5 | 0.125 |
|  | 0.125 | 110.1 | 7.3 | 6.60 | 10.1 |  |
|  | 1.25 | 81.8 | 10.3 | 12.60 | -18.2 |  |
|  | 5 | 114.8 | 11.7 | 10.17 | 14.8 |  |
| CLONAZEPAM | 0.025 | 95.2 | 8.4 | 8.83 | -4.8 | 0.125 |
|  | 0.125 | 101.9 | 14.3 | 13.99 | 1.9 |  |
|  | 1.25 | 107.3 | 7.8 | 7.25 | 7.3 |  |
|  | 5 | 117.5 | 7.9 | 6.73 | 17.5 |  |
| COCAETHYLENE | 0.025 | 102.3 | 15.0 | 14.69 | 2.3 | 0.025 |
|  | 0.125 | 91.6 | 3.7 | 4.03 | -8.4 |  |
|  | 1.25 | 109.1 | 7.7 | 7.10 | 9.1 |  |
|  | 5 | 115.2 | 13.9 | 12.08 | 15.2 |  |
| COCAINE | 0.025 | 96.9 | 19.3 | 19.91 | -3.1 | 0.125 |
|  | 0.125 | 109.0 | 17.6 | 16.12 | 9.0 |  |
|  | 1.25 | 104.5 | 10.1 | 9.68 | 4.5 |  |
|  | 5 | 110.4 | 12.8 | 11.57 | 10.4 |  |
| CODEINE | 0.025 | 125.2 | 24.2 | 19.37 | 25.2 | 0.125 |
|  | 0.125 | 103.3 | 10.6 | 10.29 | 3.3 |  |
|  | 1.25 | 103.8 | 7.7 | 7.42 | 3.8 |  |
|  | 5 | 111.5 | 6.8 | 6.13 | 11.5 |  |
| CUMYL-PICA | 0.025 | 98.8 | 6.8 | 6.88 | -1.2 | 0.025 |
|  | 0.125 | 108.7 | 5.1 | 4.68 | 8.7 |  |
|  | 1.25 | 117.3 | 10.7 | 9.09 | 17.3 |  |
| D2PM | 0.025 | 112.3 | 17.0 | 15.16 | 12.3 | 0.125 |
|  | 0.125 | 110.7 | 7.9 | 7.15 | 10.7 |  |
|  | 1.25 | 108.4 | 9.5 | 8.79 | 8.4 |  |
|  | 5 | 113.3 | 14.7 | 13.00 | 13.3 |  |
| DEMOXEPAM | 0.125 | 101.1 | 8.4 | 8.33 | 1.1 | 0.125 |
|  | 1.25 | 99.0 | 11.8 | 11.89 | -1.0 |  |
|  | 5 | 118.3 | 12.3 | 10.39 | 18.3 |  |
| DESALKYLFLURAZEPAM | 0.125 | 89.5 | 17.3 | 19.38 | -10.5 | 0.125 |
|  | 1.25 | 99.3 | 8.7 | 8.80 | -0.7 |  |
|  | 5 | 98.4 | 7.2 | 7.30 | -1.6 |  |
| DESCHLOROETIZOLAM | 0.025 | 104.6 | 14.4 | 13.80 | 4.6 | 0.125 |
|  | 0.125 | 91.7 | 14.5 | 15.78 | -8.3 |  |
|  | 1.25 | 102.2 | 7.5 | 7.35 | 2.2 |  |
|  | 5 | 105.9 | 15.0 | 14.12 | 5.9 |  |
| DESCHLORO-N-ETHYL-KETAMINE | 0.025 | 116.1 | 14.4 | 12.41 | 16.1 | 0.125 |
|  | 0.125 | 109.1 | 13.5 | 12.38 | 9.1 |  |
|  | 1.25 | 101.8 | 9.8 | 9.65 | 1.8 |  |
|  | 5 | 112.6 | 12.8 | 11.42 | 12.6 |  |
| DESIPRAMINE | 0.025 | 93.8 | 9.2 | 9.83 | -6.3 | 0.125 |
|  | 0.125 | 117.5 | 8.7 | 7.44 | 17.5 |  |
|  | 1.25 | 94.4 | 10.1 | 10.67 | -5.6 |  |
|  | 5 | 115.0 | 11.3 | 9.85 | 15.0 |  |
| DESMETHYLDIAZEPAM (NORDIAZEPAM) | 0.025 | 46.9 | 23.9 | 50.94 | -53.1 | 0.125 |
|  | 0.125 | 98.3 | 13.2 | 13.39 | -1.7 |  |
|  | 1.25 | 109.8 | 12.7 | 11.57 | 9.8 |  |
|  | 5 | 113.7 | 6.7 | 5.85 | 13.7 |  |
| DESMETHYLFLUNITRAZEPAM | 0.025 | 91.4 | 17.3 | 18.94 | -8.6 | 0.125 |
|  | 0.125 | 82.9 | 4.6 | 5.49 | -17.1 |  |
|  | 1.25 | 80.8 | 8.0 | 9.90 | -19.2 |  |
|  | 5 | 80.7 | 3.9 | 4.87 | -19.3 |  |
| DESOMORPHINE | 0.025 | 131.1 | 23.4 | 17.89 | 31.1 | 0.125 |
|  | 0.125 | 95.0 | 8.9 | 9.39 | -5.0 |  |
|  | 1.25 | 101.4 | 13.5 | 13.31 | 1.4 |  |
|  | 5 | 106.4 | 12.8 | 12.04 | 6.4 |  |
| DESOXY-D2PM | 0.025 | 125.5 | 15.6 | 12.41 | 25.5 | 0.125 |
|  | 0.125 | 94.0 | 8.6 | 9.18 | -6.0 |  |
|  | 1.25 | 94.4 | 11.5 | 12.15 | -5.6 |  |
|  | 5 | 100.6 | 13.9 | 13.85 | 0.6 |  |
| DESOXYPIPRADROL (2-DPMP) | 0.025 | 99.8 | 12.1 | 12.14 | -0.2 | 0.125 |
|  | 0.125 | 91.2 | 6.6 | 7.23 | -8.8 |  |
|  | 1.25 | 115.1 | 7.7 | 6.72 | 15.1 |  |
|  | 5 | 119.9 | 10.5 | 8.73 | 19.9 |  |
| DEXTROMETHORPHAN | 0.025 | 101.0 | 22.8 | 22.53 | 1.0 | 0.125 |
|  | 0.125 | 95.0 | 9.8 | 10.32 | -5.0 |  |
|  | 1.25 | 105.0 | 9.9 | 9.44 | 5.0 |  |
|  | 5 | 115.6 | 4.0 | 3.49 | 15.6 |  |
| DIAZEPAM | 0.025 | 94.0 | 8.2 | 8.69 | -6.0 | 0.125 |
|  | 0.125 | 88.3 | 7.0 | 7.96 | -11.8 |  |
|  | 1.25 | 101.6 | 9.1 | 8.91 | 1.6 |  |
|  | 5 | 110.4 | 10.6 | 9.58 | 10.4 |  |
| DICLAZEPAM | 0.025 | 79.9 | 30.2 | 37.77 | -20.1 | 0.125 |
|  | 0.125 | 89.9 | 10.3 | 11.48 | -10.1 |  |
|  | 1.25 | 109.9 | 11.7 | 10.65 | 9.9 |  |
|  | 5 | 118.5 | 6.6 | 5.53 | 18.5 |  |
| DICLOFENSINE | 0.025 | 120.3 | 13.0 | 10.84 | 20.3 | 0.125 |
|  | 0.125 | 97.5 | 8.7 | 8.91 | -2.5 |  |
|  | 1.25 | 94.5 | 8.6 | 9.06 | -5.5 |  |
|  | 5 | 118.6 | 10.3 | 8.65 | 18.6 |  |
| DIETHYLCATHINONE (AMFEPRAMONE) | 0.025 | 131.1 | 57.2 | 43.61 | 31.1 | 0.125 |
|  | 0.125 | 103.5 | 6.3 | 6.09 | 3.5 |  |
|  | 1.25 | 109.9 | 6.5 | 5.89 | 9.9 |  |
|  | 5 | 114.1 | 12.5 | 10.98 | 14.1 |  |
| DIHYDROCODEINE | 0.025 | 116.9 | 23.2 | 19.87 | 16.9 | 0.125 |
|  | 0.125 | 107.0 | 5.0 | 4.71 | 7.0 |  |
|  | 1.25 | 119.0 | 10.4 | 8.75 | 19.0 |  |
|  | 5 | 117.1 | 7.9 | 6.74 | 17.1 |  |
| DIMETHOCAINE | 0.025 | 110.7 | 8.5 | 7.68 | 10.7 | 0.125 |
|  | 0.125 | 101.5 | 8.8 | 8.71 | 1.5 |  |
|  | 1.25 | 109.5 | 6.4 | 5.81 | 9.5 |  |
|  | 5 | 119.4 | 14.6 | 12.20 | 19.4 |  |
| DIMETHYLONE (bk-MDDMA) | 0.025 | 111.0 | 22.8 | 20.58 | 11.0 | 0.125 |
|  | 0.125 | 93.8 | 8.3 | 8.79 | -6.2 |  |
|  | 1.25 | 98.9 | 14.1 | 14.26 | -1.1 |  |
|  | 5 | 99.7 | 7.9 | 7.90 | -0.3 |  |
| DIPHENHYDRAMINE | 0.025 | 100.0 | 17.6 | 17.56 | 0.0 | 0.125 |
|  | 0.125 | 102.9 | 8.7 | 8.48 | 2.9 |  |
|  | 1.25 | 100.2 | 8.8 | 8.80 | 0.2 |  |
|  | 5 | 106.1 | 6.5 | 6.14 | 6.1 |  |
| DIPHENIDINE | 0.025 | 107.2 | 13.4 | 12.47 | 7.1 | 0.125 |
|  | 0.125 | 105.7 | 7.1 | 6.71 | 5.7 |  |
|  | 1.25 | 94.2 | 8.6 | 9.14 | -5.8 |  |
|  | 5 | 111.6 | 11.2 | 10.07 | 11.6 |  |
| DiPT | 0.125 | 131.7 | 101.5 | 77.07 | 31.7 | 1.25 |
|  | 1.25 | 112.2 | 6.9 | 6.15 | 12.2 |  |
|  | 5 | 119.1 | 9.5 | 7.97 | 19.1 |  |
| DL-4662 | 0.025 | 95.6 | 22.8 | 23.80 | -4.4 | 0.125 |
|  | 0.125 | 100.7 | 9.8 | 9.71 | 0.7 |  |
|  | 1.25 | 107.5 | 13.4 | 12.48 | 7.5 |  |
|  | 5 | 104.5 | 10.4 | 9.99 | 4.5 |  |
| DOI | 0.025 | 103.2 | 31.1 | 30.17 | 3.2 | 0.125 |
|  | 0.125 | 95.4 | 11.7 | 12.26 | -4.6 |  |
|  | 1.25 | 118.7 | 17.2 | 14.45 | 18.7 |  |
|  | 5 | 117.0 | 4.1 | 3.53 | 17.0 |  |
| DOTHIEPIN | 0.025 | 107.9 | 18.1 | 16.81 | 7.9 | 0.125 |
|  | 0.125 | 103.9 | 7.2 | 6.92 | 3.9 |  |
|  | 1.25 | 88.5 | 8.4 | 9.52 | -11.5 |  |
|  | 5 | 101.8 | 13.7 | 13.45 | 1.8 |  |
| DOXEPIN | 0.025 | 114.6 | 19.2 | 16.72 | 14.6 | 0.125 |
|  | 0.125 | 111.3 | 11.3 | 10.15 | 11.3 |  |
|  | 1.25 | 100.2 | 12.6 | 12.56 | 0.2 |  |
|  | 5 | 112.0 | 8.2 | 7.33 | 12.0 |  |
| DOXYLAMINE | 0.025 | 40.6 | 46.2 | 113.81 | -59.4 | 1.25 |
|  | 0.125 | 53.2 | 25.3 | 47.46 | -46.8 |  |
|  | 1.25 | 100.3 | 8.8 | 8.82 | 0.3 |  |
|  | 5 | 119.8 | 4.0 | 3.37 | 19.8 |  |
| EAM-2201 | 0.025 | 113.2 | 9.6 | 8.52 | 13.2 | 0.025 |
|  | 0.125 | 116.3 | 7.6 | 6.57 | 16.3 |  |
|  | 1.25 | 118.8 | 21.2 | 17.84 | 18.8 |  |
| EDDP | 0.025 | 98.0 | 11.9 | 12.14 | -2.0 | 0.025 |
|  | 0.125 | 106.8 | 7.9 | 7.39 | 6.8 |  |
|  | 1.25 | 91.7 | 6.6 | 7.18 | -8.3 |  |
|  | 5 | 100.9 | 7.4 | 7.36 | 0.9 |  |
| EG-2201 | 0.025 | 98.3 | 18.3 | 18.63 | -1.7 | 0.025 |
|  | 0.125 | 97.8 | 14.5 | 14.78 | -2.2 |  |
|  | 1.25 | 105.9 | 8.6 | 8.10 | 5.9 |  |
| ERGOMETRINE | 0.025 | 115.6 | 5.7 | 4.94 | 15.6 | 0.125 |
|  | 0.125 | 99.1 | 6.6 | 6.67 | -0.9 |  |
|  | 1.25 | 89.1 | 15.4 | 17.24 | -10.9 |  |
|  | 5 | 94.6 | 5.9 | 6.23 | -5.4 |  |
| ESTAZOLAM | 0.025 | 103.9 | 32.8 | 31.57 | 3.9 | 0.125 |
|  | 0.125 | 112.7 | 11.3 | 10.04 | 12.7 |  |
|  | 1.25 | 102.0 | 19.3 | 18.89 | 2.0 |  |
|  | 5 | 119.7 | 3.1 | 2.56 | 19.7 |  |
| ETAQUALONE | 0.025 | 96.3 | 10.0 | 10.41 | -3.8 | 0.125 |
|  | 0.125 | 109.8 | 10.6 | 9.63 | 9.8 |  |
|  | 1.25 | 119.8 | 5.6 | 4.63 | 19.8 |  |
|  | 5 | 118.7 | 8.8 | 7.38 | 18.7 |  |
| ETHCATHINONE METABOLITE | 0.125 | 95.0 | 17.7 | 18.58 | -5.0 | 0.125 |
|  | 1.25 | 106.8 | 16.7 | 15.60 | 6.8 |  |
|  | 5 | 114.3 | 8.8 | 7.74 | 14.3 |  |
| ETHYLONE (bk-MDEA) | 0.025 | 80.1 | 22.1 | 27.58 | -19.9 | 0.125 |
|  | 0.125 | 94.6 | 5.6 | 5.91 | -5.4 |  |
|  | 1.25 | 97.1 | 9.1 | 9.34 | -2.9 |  |
|  | 5 | 106.6 | 12.6 | 11.83 | 6.6 |  |
| ETHYLPHENIDATE | 0.025 | 112.3 | 14.2 | 12.68 | 12.3 | 0.125 |
|  | 0.125 | 93.9 | 5.4 | 5.73 | -6.1 |  |
|  | 1.25 | 100.1 | 12.6 | 12.54 | 0.1 |  |
|  | 5 | 96.4 | 9.1 | 9.40 | -3.6 |  |
| ETIZOLAM | 0.025 | 92.2 | 16.6 | 18.00 | -7.8 | 0.125 |
|  | 0.125 | 100.3 | 11.3 | 11.22 | 0.3 |  |
|  | 1.25 | 108.1 | 19.2 | 17.77 | 8.1 |  |
|  | 5 | 118.9 | 6.3 | 5.32 | 18.9 |  |
| EUTYLONE (bk-EBDB) | 0.025 | 101.3 | 13.6 | 13.39 | 1.3 | 0.125 |
|  | 0.125 | 110.9 | 11.6 | 10.50 | 10.9 |  |
|  | 1.25 | 109.1 | 15.3 | 14.02 | 9.1 |  |
|  | 5 | 112.2 | 11.0 | 9.82 | 12.2 |  |
| FDU-PB-22 | 0.025 | 80.9 | 29.1 | 35.99 | -19.1 | 0.125 |
|  | 0.125 | 99.1 | 7.4 | 7.51 | -0.9 |  |
|  | 1.25 | 115.6 | 7.8 | 6.79 | 15.6 |  |
| FENFLURAMINE | 0.025 | 111.4 | 23.0 | 20.66 | 11.4 | 0.125 |
|  | 0.125 | 115.8 | 9.8 | 8.51 | 15.8 |  |
|  | 1.25 | 100.7 | 13.0 | 12.89 | 0.7 |  |
|  | 5 | 118.0 | 22.9 | 19.39 | 18.0 |  |
| FENTANYL | 0.025 | 102.4 | 22.6 | 22.05 | 2.4 | 0.125 |
|  | 0.125 | 111.8 | 12.0 | 10.70 | 11.8 |  |
|  | 1.25 | 94.8 | 7.0 | 7.35 | -5.2 |  |
|  | 5 | 103.7 | 19.7 | 19.00 | 3.7 |  |
| FLUBROMAZEPAM | 0.025 | 104.9 | 28.3 | 26.99 | 4.8 | 0.125 |
|  | 0.125 | 93.6 | 11.5 | 12.28 | -6.4 |  |
|  | 1.25 | 95.9 | 8.0 | 8.31 | -4.1 |  |
|  | 5 | 107.2 | 11.9 | 11.11 | 7.2 |  |
| FLUDIAZEPAM | 0.025 | 106.0 | 26.9 | 25.35 | 6.0 | 0.125 |
|  | 0.125 | 87.5 | 11.9 | 13.63 | -12.5 |  |
|  | 1.25 | 102.8 | 2.3 | 2.19 | 2.8 |  |
|  | 5 | 115.7 | 9.8 | 8.51 | 15.7 |  |
| FLUMAZENIL | 0.025 | 101.1 | 10.8 | 10.65 | 1.1 | 0.125 |
|  | 0.125 | 100.8 | 2.7 | 2.67 | 0.8 |  |
|  | 1.25 | 101.8 | 10.3 | 10.16 | 1.8 |  |
|  | 5 | 115.2 | 16.0 | 13.90 | 15.2 |  |
| FLUNITRAZEPAM | 0.025 | 95.5 | 14.9 | 15.58 | -4.5 | 0.125 |
|  | 0.125 | 103.3 | 9.8 | 9.51 | 3.3 |  |
|  | 1.25 | 103.8 | 7.0 | 6.77 | 3.8 |  |
|  | 5 | 118.8 | 12.0 | 10.14 | 18.8 |  |
| FLUOXETINE | 0.025 | 111.9 | 14.3 | 12.75 | 11.9 | 0.125 |
|  | 0.125 | 94.8 | 6.7 | 7.05 | -5.2 |  |
|  | 1.25 | 102.5 | 8.4 | 8.21 | 2.5 |  |
|  | 5 | 118.0 | 12.3 | 10.44 | 18.0 |  |
| FLURAZEPAM | 0.025 | 108.0 | 12.7 | 11.81 | 7.9 | 0.125 |
|  | 0.125 | 100.4 | 6.3 | 6.24 | 0.4 |  |
|  | 1.25 | 91.3 | 8.2 | 8.97 | -8.7 |  |
|  | 5 | 103.8 | 10.7 | 10.34 | 3.8 |  |
| FUB-144 | 0.025 | 100.3 | 6.3 | 6.28 | 0.3 | 0.025 |
|  | 0.125 | 101.8 | 13.8 | 13.58 | 1.8 |  |
|  | 1.25 | 116.2 | 16.0 | 13.78 | 16.2 |  |
| FUB-JWH-018 | 0.025 | 105.8 | 3.3 | 3.16 | 5.8 | 0.025 |
|  | 0.125 | 103.8 | 8.5 | 8.22 | 3.8 |  |
|  | 1.25 | 109.8 | 21.7 | 19.76 | 9.8 |  |
| FUB-NPB-22 | 0.025 | 109.4 | 14.8 | 13.54 | 9.4 | 0.025 |
|  | 0.125 | 108.2 | 8.6 | 7.94 | 8.2 |  |
|  | 1.25 | 102.0 | 17.5 | 17.20 | 2.0 |  |
| FUB-PB-22 | 0.025 | 239.2 | 151.8 | 63.45 | 139.2 | - |
|  | 0.125 | 146.1 | 118.4 | 81.04 | 46.1 |  |
|  | 1.25 | 116.6 | 19.0 | 16.28 | 16.6 |  |
| FURANYLFENTANYL (Fu-F) | 0.025 | 83.4 | 14.6 | 17.49 | -16.6 | 0.125 |
|  | 0.125 | 104.9 | 14.1 | 13.44 | 4.9 |  |
|  | 1.25 | 107.3 | 15.8 | 14.72 | 7.3 |  |
|  | 5 | 114.7 | 10.3 | 8.94 | 14.7 |  |
| HARMALINE | 0.025 | 82.1 | 19.7 | 24.01 | -17.9 | 0.125 |
|  | 0.125 | 100.8 | 8.3 | 8.28 | 0.8 |  |
|  | 1.25 | 89.8 | 9.7 | 10.77 | -10.2 |  |
|  | 5 | 97.0 | 12.5 | 12.90 | -3.0 |  |
| HARMINE | 0.025 | 141.5 | 30.8 | 21.75 | 41.5 | 0.125 |
|  | 0.125 | 92.1 | 18.4 | 20.03 | -7.9 |  |
|  | 1.25 | 94.4 | 9.4 | 9.92 | -5.6 |  |
|  | 5 | 107.8 | 7.5 | 6.99 | 7.8 |  |
| HU-210 | 0.025 | 84.6 | 9.1 | 10.80 | -15.5 | 0.025 |
|  | 0.125 | 115.6 | 18.6 | 16.09 | 15.6 |  |
|  | 1.25 | 118.8 | 5.3 | 4.42 | 18.8 |  |
| HYDROCODONE | 0.025 | 136.8 | 25.6 | 18.68 | 36.8 | 0.125 |
|  | 0.125 | 103.3 | 13.5 | 13.11 | 3.3 |  |
|  | 1.25 | 100.5 | 10.2 | 10.15 | 0.5 |  |
|  | 5 | 103.4 | 11.3 | 10.90 | 3.4 |  |
| IBOGAINE | 0.125 | 65.0 | 26.4 | 40.69 | -35.0 | 1.25 |
|  | 1.25 | 97.4 | 12.9 | 13.23 | -2.6 |  |
|  | 5 | 118.0 | 6.6 | 5.62 | 18.0 |  |
| IMIPRAMINE | 0.025 | 102.9 | 13.9 | 13.54 | 2.9 | 0.125 |
|  | 0.125 | 114.9 | 10.9 | 9.45 | 14.9 |  |
|  | 1.25 | 90.6 | 6.3 | 6.92 | -9.4 |  |
|  | 5 | 115.6 | 10.0 | 8.62 | 15.6 |  |
| ISOPENTEDRONE | 0.025 | 103.1 | 52.7 | 51.12 | 3.1 | 0.125 |
|  | 0.125 | 94.0 | 14.4 | 15.28 | -6.0 |  |
|  | 1.25 | 95.7 | 9.5 | 9.88 | -4.3 |  |
|  | 5 | 106.6 | 5.0 | 4.70 | 6.6 |  |
| JWH 018 BENZIMIDAZOLE ANALOG | 0.025 | 97.7 | 17.4 | 17.83 | -2.3 | 0.025 |
|  | 0.125 | 98.5 | 14.4 | 14.64 | -1.5 |  |
|  | 1.25 | 106.3 | 14.2 | 13.39 | 6.3 |  |
| JWH-011 | 0.025 | 111.1 | 9.9 | 8.92 | 11.1 | 0.025 |
|  | 0.125 | 110.3 | 9.5 | 8.58 | 10.3 |  |
|  | 1.25 | 116.7 | 2.9 | 2.49 | 16.7 |  |
| JWH-016 | 0.025 | 116.8 | 6.0 | 5.16 | 16.8 | 0.025 |
|  | 0.125 | 103.9 | 5.8 | 5.55 | 3.9 |  |
|  | 1.25 | 118.5 | 7.5 | 6.30 | 18.5 |  |
| JWH-018 | 0.025 | 98.7 | 10.4 | 10.54 | -1.3 | 0.025 |
|  | 0.125 | 98.4 | 4.8 | 4.86 | -1.6 |  |
|  | 1.25 | 113.1 | 16.8 | 14.85 | 13.1 |  |
| JWH-018 ADAMANTYL CARBOXAMIDE | 0.025 | 101.0 | 10.6 | 10.54 | 1.0 | 0.025 |
|  | 0.125 | 115.9 | 9.8 | 8.44 | 15.9 |  |
|  | 1.25 | 117.8 | 9.9 | 8.40 | 17.8 |  |
| JWH-020 | 0.025 | 100.5 | 10.9 | 10.90 | 0.5 | 0.025 |
|  | 0.125 | 105.4 | 5.6 | 5.27 | 5.4 |  |
|  | 1.25 | 115.6 | 7.8 | 6.79 | 15.6 |  |
| JWH-022 | 0.025 | 104.7 | 7.6 | 7.22 | 4.7 | 0.025 |
|  | 0.125 | 109.5 | 6.9 | 6.29 | 9.5 |  |
|  | 1.25 | 114.0 | 9.4 | 8.23 | 14.0 |  |
| JWH-031 | 0.025 | 91.5 | 9.4 | 10.26 | -8.5 | 0.025 |
|  | 0.125 | 102.3 | 3.0 | 2.92 | 2.3 |  |
|  | 1.25 | 117.0 | 12.4 | 10.61 | 17.0 |  |
| JWH-071 | 0.025 | 111.5 | 6.9 | 6.14 | 11.5 | 0.025 |
|  | 0.125 | 106.4 | 5.5 | 5.21 | 6.4 |  |
|  | 1.25 | 109.8 | 12.7 | 11.57 | 9.8 |  |
| JWH-073 | 0.025 | 96.6 | 10.6 | 10.93 | -3.4 | 0.025 |
|  | 0.125 | 107.6 | 9.5 | 8.79 | 7.6 |  |
|  | 1.25 | 112.9 | 10.9 | 9.67 | 12.9 |  |
| JWH-080 | 0.025 | 110.6 | 17.2 | 15.52 | 10.6 | 0.025 |
|  | 0.125 | 106.8 | 5.5 | 5.11 | 6.8 |  |
|  | 1.25 | 118.4 | 5.9 | 4.99 | 18.4 |  |
| JWH-081 | 0.025 | 114.7 | 13.1 | 11.40 | 14.7 | 0.025 |
|  | 0.125 | 111.9 | 7.9 | 7.05 | 11.9 |  |
|  | 1.25 | 113.1 | 14.8 | 13.08 | 13.1 |  |
| JWH-098 | 0.025 | 107.8 | 12.3 | 11.37 | 7.8 | 0.025 |
|  | 0.125 | 105.8 | 8.0 | 7.57 | 5.8 |  |
|  | 1.25 | 114.6 | 7.5 | 6.53 | 14.6 |  |
| JWH-116 | 0.025 | 97.5 | 7.1 | 7.24 | -2.5 | 0.025 |
|  | 0.125 | 108.5 | 3.8 | 3.53 | 8.5 |  |
|  | 1.25 | 112.8 | 13.3 | 11.79 | 12.8 |  |
| JWH-122 | 0.025 | 97.3 | 8.6 | 8.83 | -2.7 | 0.025 |
|  | 0.125 | 104.7 | 6.5 | 6.19 | 4.7 |  |
|  | 1.25 | 115.3 | 10.9 | 9.42 | 15.3 |  |
| JWH-145 | 0.025 | 113.4 | 6.3 | 5.58 | 13.4 | 0.025 |
|  | 0.125 | 108.9 | 6.1 | 5.56 | 8.9 |  |
|  | 1.25 | 110.1 | 17.2 | 15.64 | 10.1 |  |
| JWH-146 | 0.025 | 105.1 | 8.5 | 8.07 | 5.1 | 0.025 |
|  | 0.125 | 108.8 | 4.5 | 4.14 | 8.8 |  |
|  | 1.25 | 116.2 | 16.2 | 13.98 | 16.2 |  |
| JWH-147 | 0.025 | 110.3 | 12.1 | 11.00 | 10.3 | 0.025 |
|  | 0.125 | 105.9 | 9.4 | 8.85 | 5.9 |  |
|  | 1.25 | 116.4 | 15.6 | 13.38 | 16.4 |  |
| JWH-149 | 0.025 | 110.2 | 12.0 | 10.88 | 10.2 | 0.025 |
|  | 0.125 | 115.9 | 11.5 | 9.95 | 15.9 |  |
|  | 1.25 | 116.7 | 12.7 | 10.91 | 16.7 |  |
| JWH-167 | 0.025 | 100.0 | 9.3 | 9.30 | 0.0 | 0.025 |
|  | 0.125 | 104.9 | 7.4 | 7.02 | 4.8 |  |
|  | 1.25 | 112.3 | 9.5 | 8.49 | 12.3 |  |
| JWH-175 | 0.025 | 97.4 | 4.0 | 4.12 | -2.6 | 0.025 |
|  | 0.125 | 100.8 | 2.5 | 2.47 | 0.8 |  |
|  | 1.25 | 118.4 | 5.6 | 4.72 | 18.4 |  |
| JWH-182 | 0.025 | 95.4 | 7.0 | 7.36 | -4.6 | 0.025 |
|  | 0.125 | 104.3 | 10.3 | 9.88 | 4.3 |  |
|  | 1.25 | 119.9 | 7.3 | 6.07 | 19.9 |  |
| JWH-193 | 0.025 | 106.2 | 17.6 | 16.62 | 6.2 | 0.025 |
|  | 0.125 | 106.5 | 8.6 | 8.05 | 6.5 |  |
|  | 1.25 | 109.0 | 11.0 | 10.06 | 9.0 |  |
| JWH-198 | 0.025 | 88.2 | 8.0 | 9.07 | -11.8 | 0.025 |
|  | 0.125 | 109.7 | 8.5 | 7.78 | 9.7 |  |
|  | 1.25 | 114.8 | 6.2 | 5.43 | 14.8 |  |
| JWH-200 | 0.025 | 102.8 | 8.3 | 8.07 | 2.8 | 0.025 |
|  | 0.125 | 98.4 | 10.3 | 10.50 | -1.6 |  |
|  | 1.25 | 91.7 | 15.7 | 17.09 | -8.3 |  |
| JWH-201 | 0.025 | 106.8 | 16.1 | 15.04 | 6.8 | 0.025 |
|  | 0.125 | 109.9 | 11.6 | 10.53 | 9.9 |  |
|  | 1.25 | 119.4 | 18.4 | 15.40 | 19.4 |  |
| JWH-213 | 0.025 | 105.2 | 10.6 | 10.04 | 5.1 | 0.025 |
|  | 0.125 | 114.7 | 6.9 | 6.04 | 14.7 |  |
|  | 1.25 | 114.0 | 8.1 | 7.12 | 14.0 |  |
| JWH-307 | 0.025 | 104.9 | 8.3 | 7.91 | 4.9 | 0.025 |
|  | 0.125 | 119.4 | 7.0 | 5.87 | 19.4 |  |
|  | 1.25 | 116.1 | 11.6 | 10.00 | 16.1 |  |
| JWH-309 | 0.025 | 105.6 | 3.4 | 3.18 | 5.6 | 0.025 |
|  | 0.125 | 111.4 | 4.8 | 4.33 | 11.4 |  |
|  | 1.25 | 107.3 | 12.2 | 11.34 | 7.3 |  |
| JWH-368 | 0.025 | 110.1 | 11.3 | 10.25 | 10.1 | 0.025 |
|  | 0.125 | 106.4 | 9.2 | 8.66 | 6.4 |  |
|  | 1.25 | 110.0 | 17.5 | 15.93 | 10.0 |  |
| JWH-369 | 0.025 | 101.6 | 5.8 | 5.69 | 1.6 | 0.025 |
|  | 0.125 | 114.2 | 6.3 | 5.50 | 14.2 |  |
|  | 1.25 | 118.4 | 11.9 | 10.04 | 18.4 |  |
| JWH-412 | 0.025 | 109.0 | 10.4 | 9.54 | 9.0 | 0.025 |
|  | 0.125 | 114.9 | 6.1 | 5.35 | 14.9 |  |
|  | 1.25 | 119.7 | 20.6 | 17.25 | 19.7 |  |
| JWH-424 | 0.025 | 107.3 | 17.5 | 16.29 | 7.3 | 0.025 |
|  | 0.125 | 107.4 | 6.1 | 5.72 | 7.4 |  |
|  | 1.25 | 115.5 | 15.8 | 13.66 | 15.5 |  |
| KETAMINE | 0.025 | 86.2 | 10.9 | 12.67 | -13.8 | 0.125 |
|  | 0.125 | 114.2 | 12.5 | 10.91 | 14.2 |  |
|  | 1.25 | 106.9 | 16.2 | 15.18 | 6.9 |  |
|  | 5 | 111.9 | 14.6 | 13.02 | 11.9 |  |
| LAMPA | 0.025 | 99.4 | 23.3 | 23.43 | -0.6 | 0.125 |
|  | 0.125 | 80.1 | 6.2 | 7.72 | -19.9 |  |
|  | 1.25 | 84.6 | 8.7 | 10.27 | -15.4 |  |
|  | 5 | 99.9 | 5.9 | 5.86 | -0.1 |  |
| LIDOCAINE | 0.025 | 93.7 | 13.5 | 14.38 | -6.3 | 0.125 |
|  | 0.125 | 114.3 | 7.9 | 6.89 | 14.3 |  |
|  | 1.25 | 108.8 | 9.9 | 9.07 | 8.8 |  |
|  | 5 | 114.1 | 18.1 | 15.87 | 14.1 |  |
| LOPRAZOLAM | 0.125 | 82.0 | 12.8 | 15.66 | -18.0 | 0.125 |
|  | 1.25 | 102.1 | 12.3 | 12.02 | 2.1 |  |
|  | 5 | 104.7 | 9.3 | 8.91 | 4.7 |  |
| LORAZEPAM | 0.025 | 145.2 | 67.8 | 46.73 | 45.2 | 0.125 |
|  | 0.125 | 95.7 | 17.1 | 17.90 | -4.3 |  |
|  | 1.25 | 93.0 | 8.3 | 8.91 | -7.0 |  |
|  | 5 | 107.4 | 12.4 | 11.53 | 7.4 |  |
| LORMETAZEPAM | 0.025 | 82.1 | 25.8 | 31.37 | -17.9 | 0.125 |
|  | 0.125 | 108.7 | 18.7 | 17.18 | 8.7 |  |
|  | 1.25 | 104.0 | 7.6 | 7.26 | 4.0 |  |
|  | 5 | 113.5 | 7.8 | 6.85 | 13.5 |  |
| LSD | 0.025 | 101.4 | 31.7 | 31.23 | 1.4 | 0.125 |
|  | 0.125 | 104.9 | 11.2 | 10.71 | 4.9 |  |
|  | 1.25 | 81.3 | 9.6 | 11.75 | -18.7 |  |
|  | 5 | 99.1 | 8.2 | 8.31 | -0.9 |  |
| MAM-2201 | 0.025 | 107.8 | 18.1 | 16.83 | 7.8 | 0.025 |
|  | 0.125 | 113.0 | 9.5 | 8.39 | 13.0 |  |
|  | 1.25 | 116.7 | 12.6 | 10.77 | 16.7 |  |
| MAPROTILINE | 0.025 | 123.1 | 19.4 | 15.79 | 23.1 | 0.125 |
|  | 0.125 | 104.6 | 11.8 | 11.24 | 4.6 |  |
|  | 1.25 | 90.1 | 8.8 | 9.81 | -9.9 |  |
|  | 5 | 116.5 | 11.7 | 10.01 | 16.5 |  |
| MBDB | 0.025 | 138.3 | 9.3 | 6.73 | 38.3 | 0.125 |
|  | 0.125 | 108.0 | 3.8 | 3.47 | 8.0 |  |
|  | 1.25 | 113.6 | 8.4 | 7.39 | 13.6 |  |
|  | 5 | 114.8 | 13.8 | 12.00 | 14.8 |  |
| MDAT | 0.125 | 98.1 | 17.8 | 18.19 | -1.9 | 0.125 |
|  | 1.25 | 99.9 | 15.4 | 15.40 | -0.1 |  |
|  | 5 | 103.0 | 15.1 | 14.66 | 3.0 |  |
| MDBP | 0.025 | 185.9 | 25.7 | 13.80 | 85.9 | 0.125 |
|  | 0.125 | 119.2 | 6.9 | 5.81 | 19.2 |  |
|  | 1.25 | 91.2 | 13.1 | 14.38 | -8.8 |  |
|  | 5 | 90.1 | 7.8 | 8.63 | -9.9 |  |
| MDEA | 0.025 | 113.1 | 12.6 | 11.15 | 13.1 | 0.125 |
|  | 0.125 | 104.6 | 6.2 | 5.89 | 4.6 |  |
|  | 1.25 | 101.4 | 8.5 | 8.36 | 1.4 |  |
|  | 5 | 103.6 | 10.7 | 10.36 | 3.6 |  |
| MDMB-CHMICA (MMB-CHMINACA) | 0.025 | 111.3 | 5.3 | 4.76 | 11.3 | 0.025 |
|  | 0.125 | 114.6 | 5.5 | 4.82 | 14.6 |  |
|  | 1.25 | 119.4 | 13.0 | 10.88 | 19.4 |  |
| MDMB-CHMINACA | 0.025 | 98.7 | 7.0 | 7.09 | -1.3 | 0.025 |
|  | 0.125 | 112.8 | 10.0 | 8.87 | 12.8 |  |
|  | 1.25 | 117.5 | 16.9 | 14.34 | 17.5 |  |
| MDPBP | 0.025 | 113.8 | 18.5 | 16.27 | 13.8 | 0.125 |
|  | 0.125 | 104.3 | 12.7 | 12.17 | 4.3 |  |
|  | 1.25 | 99.5 | 10.3 | 10.32 | -0.5 |  |
|  | 5 | 113.4 | 6.6 | 5.82 | 13.4 |  |
| MECLONAZEPAM | 0.025 | 117.1 | 9.5 | 8.11 | 17.1 | 0.125 |
|  | 0.125 | 94.0 | 10.3 | 10.95 | -6.0 |  |
|  | 1.25 | 104.4 | 8.8 | 8.43 | 4.4 |  |
|  | 5 | 119.1 | 5.8 | 4.87 | 19.1 |  |
| MEDAZEPAM | 0.025 | 83.2 | 20.6 | 24.82 | -16.8 | 0.125 |
|  | 0.125 | 82.8 | 4.8 | 5.74 | -17.2 |  |
|  | 1.25 | 88.7 | 3.6 | 4.02 | -11.3 |  |
|  | 5 | 116.2 | 15.7 | 13.50 | 16.2 |  |
| MEMANITINE | 1.25 | 105.0 | 12.0 | 11.46 | 5.0 | 1.25 |
|  | 5 | 108.4 | 17.6 | 16.20 | 8.4 |  |
| MEPERIDINE | 0.025 | 107.9 | 24.6 | 22.77 | 7.9 | 0.125 |
|  | 0.125 | 92.7 | 17.6 | 19.03 | -7.3 |  |
|  | 1.25 | 101.8 | 11.6 | 11.35 | 1.8 |  |
|  | 5 | 117.2 | 15.0 | 12.82 | 17.2 |  |
| MEPHTETRAMINE (MTTA) | 0.025 | 73.7 | 24.0 | 32.58 | -26.3 | 0.125 |
|  | 0.125 | 96.6 | 9.9 | 10.22 | -3.4 |  |
|  | 1.25 | 86.4 | 3.8 | 4.40 | -13.6 |  |
|  | 5 | 99.4 | 9.8 | 9.82 | -0.6 |  |
| MEPIRAPIM | 0.025 | 88.0 | 11.8 | 13.46 | -12.1 | 0.025 |
|  | 0.125 | 102.9 | 7.8 | 7.54 | 2.9 |  |
|  | 1.25 | 93.7 | 6.4 | 6.83 | -6.3 |  |
| MEPROBAMATE | 0.025 | 82.9 | 69.4 | 83.76 | -17.1 | 0.125 |
|  | 0.125 | 114.4 | 11.6 | 10.12 | 14.4 |  |
|  | 1.25 | 82.2 | 11.7 | 14.29 | -17.8 |  |
|  | 5 | 94.0 | 9.5 | 10.12 | -6.0 |  |
| MESCALINE | 0.025 | 1212.9 | 631.2 | 52.04 | 1112.9 | 1.25 |
|  | 0.125 | 294.9 | 103.1 | 34.95 | 194.9 |  |
|  | 1.25 | 99.8 | 13.2 | 13.25 | -0.2 |  |
|  | 5 | 96.3 | 8.4 | 8.71 | -3.7 |  |
| METHADONE | 0.025 | 109.9 | 12.1 | 11.02 | 9.9 | 0.125 |
|  | 0.125 | 107.8 | 9.6 | 8.90 | 7.8 |  |
|  | 1.25 | 101.5 | 7.2 | 7.10 | 1.5 |  |
|  | 5 | 116.9 | 6.0 | 5.17 | 16.9 |  |
| METHAMPHETAMINE | 0.025 | 149.1 | 69.8 | 46.80 | 49.1 | 0.125 |
|  | 0.125 | 98.9 | 15.8 | 16.00 | -1.1 |  |
|  | 1.25 | 87.4 | 14.1 | 16.11 | -12.6 |  |
|  | 5 | 103.9 | 12.9 | 12.45 | 3.9 |  |
| METHANDIENONE | 0.025 | 101.5 | 15.9 | 15.64 | 1.5 | 0.125 |
|  | 0.125 | 109.1 | 7.8 | 7.12 | 9.1 |  |
|  | 1.25 | 104.0 | 12.3 | 11.81 | 4.0 |  |
|  | 5 | 110.0 | 10.9 | 9.94 | 10.0 |  |
| METHAQUALONE | 0.025 | 100.7 | 24.8 | 24.58 | 0.7 | 0.125 |
|  | 0.125 | 119.5 | 4.3 | 3.60 | 19.5 |  |
|  | 1.25 | 103.8 | 11.6 | 11.18 | 3.8 |  |
|  | 5 | 96.3 | 4.3 | 4.42 | -3.7 |  |
| METHCATHINONE | 0.025 | 109.5 | 19.8 | 18.06 | 9.5 | 0.125 |
|  | 0.125 | 97.6 | 6.5 | 6.68 | -2.4 |  |
|  | 1.25 | 95.0 | 5.7 | 6.03 | -5.0 |  |
|  | 5 | 106.0 | 10.9 | 10.32 | 6.0 |  |
| METHEDRONE (bk-PMMA) | 0.025 | 88.2 | 6.7 | 7.64 | -11.8 | 0.125 |
|  | 0.125 | 101.8 | 9.3 | 9.09 | 1.8 |  |
|  | 1.25 | 113.8 | 10.6 | 9.33 | 13.8 |  |
|  | 5 | 108.7 | 10.7 | 9.80 | 8.7 |  |
| METHOHEXITAL | 0.125 | 92.5 | 17.0 | 18.37 | -7.5 | 0.125 |
|  | 1.25 | 96.6 | 11.8 | 12.25 | -3.4 |  |
|  | 5 | 114.5 | 9.3 | 8.16 | 14.5 |  |
| METHOXETAMINE | 0.025 | 90.7 | 30.5 | 33.66 | -9.4 | 0.125 |
|  | 0.125 | 109.8 | 16.4 | 14.91 | 9.8 |  |
|  | 1.25 | 113.4 | 8.6 | 7.57 | 13.4 |  |
|  | 5 | 118.7 | 7.7 | 6.53 | 18.7 |  |
| METHOXPHENIDINE | 0.025 | 77.4 | 38.6 | 49.93 | -22.7 | 0.125 |
|  | 0.125 | 91.2 | 8.4 | 9.19 | -8.8 |  |
|  | 1.25 | 99.5 | 11.7 | 11.73 | -0.5 |  |
|  | 5 | 114.3 | 10.1 | 8.79 | 14.3 |  |
| METHYLHEXANAMINE | 0.025 | 286.6 | 231.6 | 80.83 | 186.6 | 0.125 |
|  | 0.125 | 114.5 | 13.8 | 12.01 | 14.5 |  |
|  | 1.25 | 99.2 | 10.1 | 10.22 | -0.8 |  |
|  | 5 | 111.8 | 17.9 | 16.01 | 11.8 |  |
| METHYLONE (bk-MDMA) | 0.025 | 105.7 | 6.5 | 6.15 | 5.7 | 0.125 |
|  | 0.125 | 98.7 | 8.1 | 8.20 | -1.3 |  |
|  | 1.25 | 97.9 | 8.6 | 8.81 | -2.1 |  |
|  | 5 | 99.7 | 11.0 | 11.06 | -0.3 |  |
| METHYLPHENIDATE | 0.025 | 110.4 | 10.7 | 9.66 | 10.4 | 0.125 |
|  | 0.125 | 114.1 | 19.8 | 17.38 | 14.1 |  |
|  | 1.25 | 94.9 | 12.2 | 12.84 | -5.1 |  |
|  | 5 | 99.3 | 3.6 | 3.63 | -0.7 |  |
| MEXEDRONE | 0.025 | 89.8 | 11.9 | 13.29 | -10.2 | 0.125 |
|  | 0.125 | 102.6 | 13.4 | 13.04 | 2.6 |  |
|  | 1.25 | 102.1 | 5.5 | 5.35 | 2.1 |  |
|  | 5 | 114.2 | 10.1 | 8.82 | 14.2 |  |
| MIANSERIN | 0.025 | 85.3 | 10.4 | 12.18 | -14.8 | 0.125 |
|  | 0.125 | 90.9 | 9.1 | 10.05 | -9.1 |  |
|  | 1.25 | 84.2 | 6.0 | 7.10 | -15.8 |  |
|  | 5 | 103.7 | 10.8 | 10.38 | 3.7 |  |
| MIDAZOLAM | 0.025 | 108.3 | 12.0 | 11.10 | 8.3 | 0.125 |
|  | 0.125 | 96.2 | 8.3 | 8.62 | -3.8 |  |
|  | 1.25 | 81.2 | 2.2 | 2.66 | -18.8 |  |
|  | 5 | 102.3 | 6.6 | 6.48 | 2.3 |  |
| MITRAGYNINE | 0.025 | 82.3 | 20.5 | 24.96 | -17.7 | 0.125 |
|  | 0.125 | 93.4 | 13.0 | 13.90 | -6.6 |  |
|  | 1.25 | 100.6 | 6.6 | 6.54 | 0.6 |  |
|  | 5 | 114.9 | 9.2 | 8.05 | 14.9 |  |
| MMAI | 0.125 | 105.2 | 8.3 | 7.90 | 5.2 | 0.125 |
|  | 1.25 | 93.0 | 11.9 | 12.82 | -7.0 |  |
|  | 5 | 100.6 | 12.0 | 11.93 | 0.6 |  |
| MMB018 | 0.025 | 104.9 | 6.1 | 5.85 | 4.9 | 0.025 |
|  | 0.125 | 105.9 | 8.9 | 8.42 | 5.9 |  |
|  | 1.25 | 117.2 | 9.9 | 8.45 | 17.2 |  |
| MMB2201 | 0.025 | 107.0 | 4.9 | 4.60 | 7.0 | 0.025 |
|  | 0.125 | 108.2 | 5.3 | 4.87 | 8.2 |  |
|  | 1.25 | 110.4 | 11.8 | 10.68 | 10.4 |  |
| MN-18 | 0.025 | 100.1 | 12.3 | 12.26 | 0.1 | 0.025 |
|  | 0.125 | 102.7 | 11.2 | 10.87 | 2.7 |  |
|  | 1.25 | 113.5 | 7.0 | 6.14 | 13.5 |  |
| MN-25 | 0.025 | 100.2 | 17.5 | 17.44 | 0.1 | 0.025 |
|  | 0.125 | 98.1 | 9.5 | 9.68 | -1.9 |  |
|  | 1.25 | 105.5 | 7.3 | 6.90 | 5.5 |  |
| MN-25-2-METHYL DERIVATIVE | 0.025 | 99.3 | 7.9 | 7.99 | -0.7 | 0.025 |
|  | 0.125 | 105.7 | 6.5 | 6.10 | 5.7 |  |
|  | 1.25 | 104.2 | 8.8 | 8.46 | 4.2 |  |
| MO-CHMINACA | 0.025 | 113.2 | 11.9 | 10.54 | 13.2 | 0.025 |
|  | 0.125 | 115.5 | 8.9 | 7.68 | 15.5 |  |
|  | 1.25 | 116.9 | 11.4 | 9.71 | 16.9 |  |
| MORPHINE | 0.025 | 142.7 | 12.2 | 8.55 | 42.7 | 0.125 |
|  | 0.125 | 111.0 | 6.1 | 5.50 | 11.0 |  |
|  | 1.25 | 114.9 | 4.9 | 4.30 | 14.9 |  |
|  | 5 | 119.7 | 3.8 | 3.15 | 19.7 |  |
| MT-45 | 0.025 | 93.7 | 18.5 | 19.77 | -6.3 | 0.125 |
|  | 0.125 | 91.6 | 5.2 | 5.62 | -8.4 |  |
|  | 1.25 | 114.6 | 9.8 | 8.54 | 14.6 |  |
|  | 5 | 119.0 | 16.2 | 13.62 | 19.0 |  |
| N-(3-METHYLBENZYL)PIPERAZINE | 0.025 | 155.6 | 15.8 | 10.18 | 55.6 | 0.125 |
|  | 0.125 | 109.1 | 5.1 | 4.65 | 9.1 |  |
|  | 1.25 | 82.6 | 1.9 | 2.30 | -17.4 |  |
|  | 5 | 96.7 | 15.1 | 15.58 | -3.3 |  |
| N.N-DIETHYLPHENETHYLAMINE | 0.025 | 105.3 | 14.8 | 14.04 | 5.3 | 0.125 |
|  | 0.125 | 104.0 | 10.1 | 9.73 | 4.0 |  |
|  | 1.25 | 93.6 | 6.2 | 6.63 | -6.4 |  |
|  | 5 | 105.4 | 6.1 | 5.75 | 5.4 |  |
| N.N-DIMETHYLPENTYLONE (bk-DMBDP) | 0.025 | 102.5 | 11.9 | 11.64 | 2.5 | 0.125 |
|  | 0.125 | 107.9 | 14.2 | 13.18 | 7.9 |  |
|  | 1.25 | 95.5 | 12.2 | 12.76 | -4.5 |  |
|  | 5 | 112.4 | 10.5 | 9.37 | 12.4 |  |
| N.N-DMT | 0.125 | 101.6 | 19.2 | 18.85 | 1.6 | 0.125 |
|  | 1.25 | 96.6 | 13.1 | 13.54 | -3.4 |  |
|  | 5 | 104.7 | 9.0 | 8.63 | 4.7 |  |
| N-ACETYL-3.4-MDMC | 0.025 | 123.2 | 19.7 | 16.00 | 23.2 | 0.125 |
|  | 0.125 | 91.5 | 6.3 | 6.87 | -8.5 |  |
|  | 1.25 | 101.0 | 9.9 | 9.84 | 1.0 |  |
|  | 5 | 110.3 | 15.8 | 14.29 | 10.3 |  |
| NAPHYRONE | 0.025 | 110.0 | 13.9 | 12.67 | 10.0 | 0.125 |
|  | 0.125 | 112.5 | 6.4 | 5.66 | 12.5 |  |
|  | 1.25 | 101.6 | 4.8 | 4.77 | 1.6 |  |
|  | 5 | 107.3 | 3.9 | 3.64 | 7.3 |  |
| NAPHYRONE-1-NAPHTYL ISOMER | 0.025 | 99.1 | 14.4 | 14.56 | -0.9 | 0.125 |
|  | 0.125 | 106.4 | 8.0 | 7.51 | 6.4 |  |
|  | 1.25 | 99.8 | 9.4 | 9.45 | -0.2 |  |
|  | 5 | 118.2 | 13.2 | 11.18 | 18.2 |  |
| N-BENZYLNORBUTYLONE | 0.025 | 75.0 | 6.8 | 9.11 | -25.0 | 0.125 |
|  | 0.125 | 96.4 | 12.2 | 12.71 | -3.6 |  |
|  | 1.25 | 90.9 | 10.8 | 11.91 | -9.1 |  |
|  | 5 | 97.6 | 12.5 | 12.85 | -2.4 |  |
| N-ETHYL-4-METHOXYAMPHETAMNE | 0.025 | 98.6 | 18.0 | 18.30 | -1.5 | 0.125 |
|  | 0.125 | 107.0 | 13.6 | 12.75 | 6.9 |  |
|  | 1.25 | 105.3 | 8.8 | 8.31 | 5.3 |  |
|  | 5 | 112.1 | 10.3 | 9.19 | 12.1 |  |
| N-ETHYLBUPHEDRONE | 0.025 | 96.9 | 19.6 | 20.25 | -3.1 | 0.125 |
|  | 0.125 | 103.8 | 8.5 | 8.21 | 3.8 |  |
|  | 1.25 | 104.6 | 8.8 | 8.38 | 4.6 |  |
|  | 5 | 113.9 | 11.9 | 10.46 | 13.9 |  |
| N-ETHYL-N-METHYLCATHINONE | 0.025 | 96.6 | 27.8 | 28.76 | -3.4 | 0.125 |
|  | 0.125 | 97.6 | 9.1 | 9.37 | -2.4 |  |
|  | 1.25 | 98.6 | 9.8 | 9.95 | -1.4 |  |
|  | 5 | 106.7 | 15.2 | 14.22 | 6.7 |  |
| N-ETHYLNORDAZEPAM | 0.025 | 82.7 | 12.2 | 14.75 | -17.3 | 0.125 |
|  | 0.125 | 89.9 | 7.1 | 7.93 | -10.1 |  |
|  | 1.25 | 89.2 | 6.9 | 7.75 | -10.8 |  |
|  | 5 | 96.5 | 9.1 | 9.45 | -3.5 |  |
| N-ETHYLNORKETAMINE | 0.025 | 107.8 | 25.2 | 23.42 | 7.8 | 0.125 |
|  | 0.125 | 110.4 | 19.8 | 17.94 | 10.4 |  |
|  | 1.25 | 114.9 | 10.5 | 9.15 | 14.9 |  |
|  | 5 | 114.7 | 6.0 | 5.27 | 14.7 |  |
| N-ETHYLOXAZEPAM | 0.025 | 91.5 | 12.9 | 14.13 | -8.5 | 0.125 |
|  | 0.125 | 95.6 | 13.6 | 14.19 | -4.4 |  |
|  | 1.25 | 103.3 | 8.1 | 7.82 | 3.3 |  |
|  | 5 | 117.5 | 7.5 | 6.40 | 17.5 |  |
| N-ETHYLPENTYLONE | 0.025 | 113.2 | 12.2 | 10.82 | 13.2 | 0.125 |
|  | 0.125 | 109.1 | 10.9 | 9.95 | 9.1 |  |
|  | 1.25 | 91.8 | 10.6 | 11.55 | -8.2 |  |
|  | 5 | 105.3 | 11.1 | 10.53 | 5.3 |  |
| NIMETAZEPAM | 0.025 | 107.9 | 10.6 | 9.84 | 7.9 | 0.125 |
|  | 0.125 | 98.8 | 8.1 | 8.20 | -1.2 |  |
|  | 1.25 | 102.1 | 9.2 | 9.02 | 2.1 |  |
|  | 5 | 108.3 | 13.6 | 12.59 | 8.3 |  |
| NITRAZEPAM | 0.025 | 101.5 | 14.2 | 14.01 | 1.5 | 0.125 |
|  | 0.125 | 89.2 | 8.6 | 9.67 | -10.8 |  |
|  | 1.25 | 94.4 | 4.1 | 4.38 | -5.6 |  |
|  | 5 | 100.1 | 7.0 | 7.04 | 0.1 |  |
| NM2201 | 0.025 | 104.0 | 17.6 | 16.97 | 4.0 | 0.025 |
|  | 0.125 | 104.7 | 8.4 | 8.07 | 4.7 |  |
|  | 1.25 | 113.0 | 13.0 | 11.54 | 13.0 |  |
| N-METHYL-2AI | 0.025 | 111.5 | 7.9 | 7.06 | 11.5 | 0.125 |
|  | 0.125 | 98.5 | 5.7 | 5.75 | -1.5 |  |
|  | 1.25 | 96.5 | 7.8 | 8.08 | -3.5 |  |
|  | 5 | 93.0 | 10.4 | 11.22 | -7.0 |  |
| N-METHYL-PEA | 0.025 | 120.5 | 9.6 | 7.98 | 20.5 | 0.125 |
|  | 0.125 | 105.1 | 4.1 | 3.87 | 5.1 |  |
|  | 1.25 | 99.4 | 6.5 | 6.54 | -0.6 |  |
|  | 5 | 103.0 | 2.7 | 2.62 | 3.0 |  |
| N-METHYLTRYPTAMINE | 0.025 | 98.3 | 17.6 | 17.87 | -1.7 | 0.125 |
|  | 0.125 | 98.7 | 6.7 | 6.75 | -1.3 |  |
|  | 1.25 | 95.7 | 8.2 | 8.57 | -4.3 |  |
|  | 5 | 89.7 | 14.1 | 15.76 | -10.3 |  |
| NN-DMC | 0.025 | 101.2 | 14.6 | 14.43 | 1.1 | 0.125 |
|  | 0.125 | 101.4 | 4.1 | 4.03 | 1.4 |  |
|  | 1.25 | 99.6 | 4.4 | 4.44 | -0.4 |  |
|  | 5 | 104.4 | 8.8 | 8.40 | 4.4 |  |
| NNEI | 0.025 | 104.0 | 16.3 | 15.70 | 4.0 | 0.025 |
|  | 0.125 | 105.8 | 5.7 | 5.41 | 5.8 |  |
|  | 1.25 | 114.3 | 8.8 | 7.73 | 14.3 |  |
| NORCLOBAZAM | 0.125 | 87.5 | 12.0 | 13.68 | -12.5 | 0.125 |
|  | 1.25 | 110.2 | 9.5 | 8.66 | 10.2 |  |
|  | 5 | 117.2 | 6.8 | 5.83 | 17.2 |  |
| NORCLOMIPRAMINE | 0.025 | 105.3 | 15.4 | 14.60 | 5.3 | 0.125 |
|  | 0.125 | 106.1 | 9.1 | 8.58 | 6.1 |  |
|  | 1.25 | 83.7 | 11.8 | 14.15 | -16.3 |  |
|  | 5 | 104.1 | 15.7 | 15.10 | 4.1 |  |
| NORKETAMINE | 0.125 | 82.3 | 15.4 | 18.68 | -17.8 | 0.125 |
|  | 1.25 | 91.9 | 7.9 | 8.60 | -8.1 |  |
|  | 5 | 108.1 | 10.7 | 9.86 | 8.1 |  |
| NORTRIPTYLINE | 0.025 | 104.2 | 19.4 | 18.62 | 4.2 | 0.125 |
|  | 0.125 | 108.1 | 6.7 | 6.20 | 8.1 |  |
|  | 1.25 | 94.4 | 9.7 | 10.27 | -5.6 |  |
|  | 5 | 106.7 | 12.2 | 11.46 | 6.7 |  |
| N-PROPYLAMPHETAMINE | 0.025 | 89.2 | 30.0 | 33.65 | -10.8 | 0.125 |
|  | 0.125 | 90.5 | 17.0 | 18.77 | -9.5 |  |
|  | 1.25 | 108.7 | 17.5 | 16.08 | 8.7 |  |
|  | 5 | 110.1 | 12.2 | 11.09 | 10.1 |  |
| NRG-3 | 0.025 | 101.7 | 13.9 | 13.66 | 1.7 | 0.125 |
|  | 0.125 | 86.5 | 4.1 | 4.69 | -13.5 |  |
|  | 1.25 | 87.4 | 6.5 | 7.39 | -12.6 |  |
|  | 5 | 105.3 | 7.1 | 6.71 | 5.3 |  |
| o-CPP | 0.025 | 143.6 | 22.9 | 15.96 | 43.6 | 0.125 |
|  | 0.125 | 100.8 | 17.1 | 16.95 | 0.8 |  |
|  | 1.25 | 107.5 | 10.7 | 9.92 | 7.5 |  |
|  | 5 | 110.3 | 9.3 | 8.39 | 10.3 |  |
| OCTACAINE | 0.025 | 87.8 | 10.3 | 11.78 | -12.3 | 0.125 |
|  | 0.125 | 113.5 | 11.8 | 10.36 | 13.5 |  |
|  | 1.25 | 99.1 | 6.8 | 6.90 | -0.9 |  |
|  | 5 | 103.7 | 8.2 | 7.87 | 3.7 |  |
| ORG-28611 | 0.025 | 98.8 | 8.4 | 8.46 | -1.2 | 0.025 |
|  | 0.125 | 113.5 | 7.5 | 6.61 | 13.5 |  |
|  | 1.25 | 85.0 | 5.7 | 6.67 | -15.0 |  |
| OXAZEPAM | 0.025 | 78.0 | 56.5 | 72.50 | -22.0 | 0.125 |
|  | 0.125 | 93.3 | 14.8 | 15.83 | -6.7 |  |
|  | 1.25 | 94.9 | 10.6 | 11.21 | -5.1 |  |
|  | 5 | 111.3 | 6.7 | 6.06 | 11.3 |  |
| OXYMORPHONE | 0.025 | 572.2 | 362.7 | 63.39 | 472.2 | - |
|  | 0.125 | 342.5 | 14.6 | 4.27 | 242.5 |  |
|  | 1.25 | 250.4 | 237.3 | 94.77 | 150.4 |  |
|  | 5 | 521.3 | 285.2 | 54.71 | 421.3 |  |
| PARACETAMOL | 0.025 | 70.4 | 19.3 | 27.35 | -29.6 | 0.125 |
|  | 0.125 | 85.4 | 5.8 | 6.74 | -14.6 |  |
|  | 1.25 | 102.9 | 6.7 | 6.53 | 2.9 |  |
|  | 5 | 105.8 | 5.5 | 5.24 | 5.8 |  |
| PAROXETINE | 0.025 | 115.3 | 16.6 | 14.41 | 15.3 | 0.125 |
|  | 0.125 | 103.5 | 17.3 | 16.70 | 3.5 |  |
|  | 1.25 | 82.0 | 6.6 | 8.03 | -18.0 |  |
|  | 5 | 104.4 | 19.0 | 18.20 | 4.4 |  |
| PB-22 | 0.025 | 106.5 | 11.4 | 10.73 | 6.5 | 0.025 |
|  | 0.125 | 109.3 | 10.8 | 9.86 | 9.3 |  |
|  | 1.25 | 116.0 | 13.3 | 11.43 | 16.0 |  |
| PCEEA | 0.025 | 102.3 | 5.8 | 5.64 | 2.3 | 0.125 |
|  | 0.125 | 91.8 | 9.8 | 10.73 | -8.2 |  |
|  | 1.25 | 99.9 | 4.8 | 4.79 | -0.1 |  |
|  | 5 | 109.6 | 5.3 | 4.83 | 9.6 |  |
| PCMPA | 0.025 | 101.9 | 13.7 | 13.41 | 1.9 | 0.125 |
|  | 0.125 | 117.3 | 15.9 | 13.58 | 17.3 |  |
|  | 1.25 | 118.3 | 8.9 | 7.53 | 18.3 |  |
|  | 5 | 113.5 | 13.4 | 11.83 | 13.5 |  |
| PCPr | 0.125 | 95.7 | 11.7 | 12.18 | -4.3 | 0.125 |
|  | 1.25 | 116.4 | 8.6 | 7.41 | 16.4 |  |
|  | 5 | 114.4 | 2.2 | 1.91 | 14.4 |  |
| PENTEDRONE | 0.025 | 87.2 | 22.2 | 25.51 | -12.8 | 0.125 |
|  | 0.125 | 99.2 | 10.1 | 10.19 | -0.8 |  |
|  | 1.25 | 89.5 | 13.0 | 14.56 | -10.5 |  |
|  | 5 | 101.9 | 8.6 | 8.45 | 1.9 |  |
| PENTEDRONE METABOLITE | 0.025 | 114.0 | 4.8 | 4.17 | 14.0 | 0.125 |
|  | 0.125 | 107.1 | 6.6 | 6.15 | 7.1 |  |
|  | 1.25 | 109.4 | 10.3 | 9.45 | 9.4 |  |
|  | 5 | 107.4 | 15.8 | 14.68 | 7.4 |  |
| PENTYLONE (bk-MBDP) | 0.025 | 47.1 | 39.4 | 83.84 | -53.0 | 0.125 |
|  | 0.125 | 112.8 | 17.3 | 15.37 | 12.8 |  |
|  | 1.25 | 111.3 | 18.6 | 16.69 | 11.3 |  |
|  | 5 | 114.5 | 14.5 | 12.62 | 14.5 |  |
| PHENAZEPAM | 0.025 | 81.6 | 32.7 | 40.01 | -18.4 | 0.125 |
|  | 0.125 | 92.7 | 15.9 | 17.15 | -7.3 |  |
|  | 1.25 | 107.6 | 10.9 | 10.08 | 7.6 |  |
|  | 5 | 113.3 | 5.2 | 4.63 | 13.3 |  |
| PHENCYCLIDINE (PCP) | 0.025 | 99.7 | 33.1 | 33.23 | -0.4 | 0.125 |
|  | 0.125 | 113.2 | 4.9 | 4.37 | 13.2 |  |
|  | 1.25 | 116.0 | 8.6 | 7.42 | 16.0 |  |
|  | 5 | 96.7 | 11.7 | 12.13 | -3.3 |  |
| PHENTERMINE | 0.025 | 99.7 | 58.0 | 58.17 | -0.3 | 0.125 |
|  | 0.125 | 90.9 | 15.4 | 16.91 | -9.1 |  |
|  | 1.25 | 112.1 | 20.3 | 18.15 | 12.1 |  |
|  | 5 | 102.8 | 13.8 | 13.40 | 2.8 |  |
| PHENYTOIN | 0.025 | 111.8 | 18.3 | 16.39 | 11.8 | 0.125 |
|  | 0.125 | 98.6 | 10.0 | 10.13 | -1.4 |  |
|  | 1.25 | 106.1 | 9.3 | 8.72 | 6.1 |  |
|  | 5 | 116.6 | 7.7 | 6.61 | 16.6 |  |
| PRAVADOLINE | 0.025 | 95.2 | 13.4 | 14.03 | -4.8 | 0.125 |
|  | 0.125 | 100.0 | 11.2 | 11.20 | 0.0 |  |
|  | 1.25 | 90.5 | 15.5 | 17.12 | -9.5 |  |
|  | 5 | 102.3 | 14.9 | 14.55 | 2.3 |  |
| PRAZEPAM | 0.025 | 99.5 | 6.0 | 6.02 | -0.5 | 0.125 |
|  | 0.125 | 107.9 | 4.5 | 4.16 | 7.9 |  |
|  | 1.25 | 99.1 | 4.6 | 4.64 | -0.9 |  |
|  | 5 | 94.4 | 4.5 | 4.76 | -5.6 |  |
| PREGABALIN | 0.125 | 64.2 | 12.3 | 19.23 | -35.8 | 1.25 |
|  | 1.25 | 94.6 | 8.6 | 9.14 | -5.4 |  |
|  | 5 | 93.8 | 5.1 | 5.42 | -6.2 |  |
| PROCAINE | 0.025 | 118.6 | 18.8 | 15.86 | 18.6 | 0.125 |
|  | 0.125 | 117.1 | 5.2 | 4.47 | 17.1 |  |
|  | 1.25 | 116.2 | 6.5 | 5.63 | 16.2 |  |
|  | 5 | 119.2 | 7.5 | 6.27 | 19.2 |  |
| PROLINTANE | 0.025 | 123.8 | 33.8 | 27.32 | 23.8 | 0.125 |
|  | 0.125 | 111.1 | 18.0 | 16.22 | 11.1 |  |
|  | 1.25 | 107.8 | 9.2 | 8.51 | 7.8 |  |
|  | 5 | 109.9 | 11.2 | 10.23 | 9.9 |  |
| PROMETHAZINE | 0.025 | 88.9 | 4.9 | 5.46 | -11.1 | 0.125 |
|  | 0.125 | 93.1 | 9.7 | 10.45 | -6.9 |  |
|  | 1.25 | 80.1 | 3.6 | 4.55 | -19.9 |  |
|  | 5 | 96.9 | 12.6 | 13.01 | -3.1 |  |
| PROPAFENONE | 0.025 | 127.6 | 11.3 | 8.84 | 27.6 | 0.125 |
|  | 0.125 | 97.9 | 8.6 | 8.74 | -2.1 |  |
|  | 1.25 | 90.0 | 7.4 | 8.18 | -10.0 |  |
|  | 5 | 105.7 | 13.6 | 12.83 | 5.7 |  |
| PROPRANOLOL | 0.025 | 71.8 | 24.6 | 34.18 | -28.2 | 0.125 |
|  | 0.125 | 96.1 | 11.5 | 11.91 | -3.9 |  |
|  | 1.25 | 86.6 | 5.4 | 6.28 | -13.4 |  |
|  | 5 | 110.3 | 19.6 | 17.80 | 10.3 |  |
| PROPYLHEXEDRINE | 0.025 | 85.0 | 57.4 | 67.53 | -15.0 | 0.125 |
|  | 0.125 | 95.0 | 17.2 | 18.11 | -5.0 |  |
|  | 1.25 | 113.4 | 18.0 | 15.92 | 13.4 |  |
|  | 5 | 118.7 | 9.1 | 7.65 | 18.7 |  |
| PSB-SB-1202 | 0.025 | 106.6 | 18.5 | 17.32 | 6.6 | 0.025 |
|  | 0.125 | 108.6 | 13.3 | 12.28 | 8.6 |  |
|  | 1.25 | 114.4 | 7.7 | 6.74 | 14.4 |  |
| PTI-1 | 0.025 | 107.7 | 7.5 | 6.98 | 7.7 | 0.025 |
|  | 0.125 | 103.1 | 6.3 | 6.13 | 3.1 |  |
|  | 1.25 | 114.9 | 9.1 | 7.92 | 14.9 |  |
| PTI-2 | 0.025 | 100.8 | 9.4 | 9.29 | 0.8 | 0.025 |
|  | 0.125 | 101.3 | 3.0 | 2.94 | 1.3 |  |
|  | 1.25 | 118.4 | 15.6 | 13.17 | 18.4 |  |
| PV-8 | 0.025 | 97.6 | 18.8 | 19.23 | -2.5 | 0.125 |
|  | 0.125 | 108.9 | 13.7 | 12.59 | 8.9 |  |
|  | 1.25 | 91.4 | 8.1 | 8.90 | -8.6 |  |
|  | 5 | 114.3 | 5.3 | 4.67 | 14.3 |  |
| PV9 | 0.025 | 115.1 | 7.9 | 6.90 | 15.1 | 0.125 |
|  | 0.125 | 101.8 | 7.7 | 7.55 | 1.8 |  |
|  | 1.25 | 94.1 | 6.0 | 6.36 | -5.9 |  |
|  | 5 | 108.1 | 5.9 | 5.45 | 8.1 |  |
| PX-1 | 0.025 | 114.6 | 17.9 | 15.58 | 14.6 | 0.025 |
|  | 0.125 | 99.6 | 5.6 | 5.59 | -0.4 |  |
|  | 1.25 | 93.4 | 11.4 | 12.16 | -6.6 |  |
| PX-2 | 0.025 | 109.2 | 12.1 | 11.09 | 9.2 | 0.025 |
|  | 0.125 | 98.9 | 5.2 | 5.21 | -1.1 |  |
|  | 1.25 | 112.6 | 12.8 | 11.37 | 12.6 |  |
| PYRAZOLAM | 0.025 | 96.7 | 17.3 | 17.89 | -3.3 | 0.125 |
|  | 0.125 | 87.0 | 11.5 | 13.23 | -13.0 |  |
|  | 1.25 | 95.6 | 4.5 | 4.69 | -4.4 |  |
|  | 5 | 117.3 | 14.3 | 12.19 | 17.3 |  |
| QUETIAPINE | 0.025 | 84.7 | 18.0 | 21.29 | -15.4 | 0.125 |
|  | 0.125 | 88.6 | 10.3 | 11.58 | -11.4 |  |
|  | 1.25 | 88.0 | 11.7 | 13.29 | -12.0 |  |
|  | 5 | 107.7 | 20.5 | 19.05 | 7.7 |  |
| RCS-4 | 0.025 | 103.4 | 19.6 | 18.96 | 3.4 | 0.025 |
|  | 0.125 | 117.3 | 9.3 | 7.91 | 17.3 |  |
|  | 1.25 | 112.1 | 17.1 | 15.26 | 12.1 |  |
| RH-34 | 0.025 | 90.6 | 19.1 | 21.06 | -9.5 | 0.125 |
|  | 0.125 | 102.6 | 16.9 | 16.50 | 2.6 |  |
|  | 1.25 | 91.5 | 13.0 | 14.20 | -8.5 |  |
|  | 5 | 110.9 | 16.6 | 14.96 | 10.9 |  |
| R-MMC | 0.025 | 133.6 | 24.2 | 18.12 | 33.6 | 0.125 |
|  | 0.125 | 112.0 | 8.8 | 7.89 | 12.0 |  |
|  | 1.25 | 119.9 | 9.7 | 8.07 | 19.9 |  |
|  | 5 | 114.1 | 8.9 | 7.76 | 14.1 |  |
| SCOPOLAMINE | 0.025 | 112.3 | 13.7 | 12.23 | 12.3 | 0.125 |
|  | 0.125 | 110.1 | 15.8 | 14.38 | 10.1 |  |
|  | 1.25 | 101.2 | 8.5 | 8.44 | 1.2 |  |
|  | 5 | 99.1 | 11.1 | 11.23 | -0.9 |  |
| SDB-005 | 0.025 | 100.5 | 11.7 | 11.60 | 0.5 | 0.025 |
|  | 0.125 | 112.0 | 5.8 | 5.18 | 12.0 |  |
|  | 1.25 | 107.9 | 10.0 | 9.23 | 7.9 |  |
| SDB-006 | 0.025 | 97.2 | 6.2 | 6.39 | -2.8 | 0.025 |
|  | 0.125 | 115.7 | 7.8 | 6.71 | 15.7 |  |
|  | 1.25 | 112.2 | 11.6 | 10.31 | 12.2 |  |
| SERTRALINE | 0.025 | 88.4 | 23.9 | 26.99 | -11.6 | 0.125 |
|  | 0.125 | 99.1 | 10.9 | 11.00 | -0.9 |  |
|  | 1.25 | 101.8 | 18.2 | 17.92 | 1.8 |  |
|  | 5 | 118.4 | 4.7 | 4.00 | 18.4 |  |
| SILDENAFIL | 0.025 | 100.9 | 21.4 | 21.17 | 0.9 | 0.125 |
|  | 0.125 | 117.0 | 10.5 | 8.98 | 17.0 |  |
|  | 1.25 | 98.2 | 16.3 | 16.62 | -1.8 |  |
|  | 5 | 107.4 | 10.6 | 9.88 | 7.4 |  |
| STANOZOLOL | 0.025 | 3064.3 | 2512.9 | 82.01 | 2964.3 | - |
|  | 1.25 | 120.8 | 53.7 | 44.44 | 20.8 |  |
|  | 5 | 38.1 | 4.1 | 10.81 | -61.9 |  |
| TAPENTADOL | 0.025 | 116.3 | 22.1 | 18.98 | 16.3 | 0.125 |
|  | 0.125 | 101.0 | 15.6 | 15.45 | 1.0 |  |
|  | 1.25 | 98.6 | 10.8 | 10.93 | -1.4 |  |
|  | 5 | 94.1 | 7.9 | 8.42 | -5.9 |  |
| TEMAZEPAM | 0.025 | 106.7 | 17.1 | 16.05 | 6.7 | 0.125 |
|  | 0.125 | 97.9 | 15.3 | 15.65 | -2.1 |  |
|  | 1.25 | 104.3 | 6.5 | 6.27 | 4.3 |  |
|  | 5 | 112.1 | 10.4 | 9.29 | 12.1 |  |
| TETRACAINE | 0.025 | 110.8 | 14.7 | 13.28 | 10.8 | 0.125 |
|  | 0.125 | 100.2 | 9.6 | 9.59 | 0.2 |  |
|  | 1.25 | 102.2 | 8.0 | 7.81 | 2.2 |  |
|  | 5 | 118.0 | 7.6 | 6.45 | 18.0 |  |
| THC | 0.025 | 94.6 | 10.2 | 10.84 | -5.5 | 0.025 |
|  | 0.125 | 98.2 | 13.0 | 13.26 | -1.8 |  |
|  | 1.25 | 115.1 | 12.1 | 10.49 | 15.1 |  |
| THCCOOH | 0.021.25 | 83.6 | 14.1 | 16.93 | -16.4 | 0.250 |
|  | 1.25 | 90.3 | 13.6 | 15.06 | -9.7 |  |
|  | 1.25 | 101.4 | 16.0 | 15.81 | 1.4 |  |
| THIOPROPAMINE | 0.025 | 122.9 | 42.5 | 34.58 | 22.9 | 0.125 |
|  | 0.125 | 103.3 | 11.0 | 10.66 | 3.3 |  |
|  | 1.25 | 101.6 | 7.0 | 6.86 | 1.6 |  |
|  | 5 | 105.9 | 9.9 | 9.37 | 5.9 |  |
| THJ | 0.025 | 95.3 | 4.5 | 4.75 | -4.7 | 0.025 |
|  | 0.125 | 94.9 | 5.3 | 5.59 | -5.1 |  |
|  | 1.25 | 116.0 | 9.3 | 8.02 | 16.0 |  |
| THJ-018 | 0.025 | 104.9 | 18.1 | 17.28 | 4.9 | 0.025 |
|  | 0.125 | 109.1 | 5.2 | 4.78 | 9.1 |  |
|  | 1.25 | 118.1 | 12.0 | 10.20 | 18.1 |  |
| THJ-2201 (5-FLUORO THJ-018) | 0.025 | 96.8 | 12.5 | 12.94 | -3.2 | 0.025 |
|  | 0.125 | 107.6 | 8.9 | 8.23 | 7.6 |  |
|  | 1.25 | 116.8 | 17.7 | 15.14 | 16.8 |  |
| TILETAMINE | 0.025 | 91.3 | 18.6 | 20.38 | -8.7 | 0.125 |
|  | 0.125 | 98.8 | 10.9 | 11.01 | -1.2 |  |
|  | 1.25 | 109.4 | 6.2 | 5.64 | 9.4 |  |
|  | 5 | 111.6 | 12.1 | 10.88 | 11.6 |  |
| TRAMADOL | 0.025 | 107.8 | 11.0 | 10.22 | 7.8 | 0.125 |
|  | 0.125 | 101.2 | 9.1 | 9.00 | 1.2 |  |
|  | 1.25 | 100.1 | 9.5 | 9.53 | 0.1 |  |
|  | 5 | 104.1 | 8.4 | 8.05 | 4.1 |  |
| TRAZODONE | 0.025 | 93.2 | 22.2 | 23.85 | -6.8 | 0.125 |
|  | 0.125 | 86.2 | 11.3 | 13.07 | -13.8 |  |
|  | 1.25 | 88.5 | 6.0 | 6.81 | -11.5 |  |
|  | 5 | 99.0 | 9.5 | 9.55 | -1.0 |  |
| TRIAZOLAM | 0.025 | 75.3 | 20.5 | 27.26 | -24.8 | 0.125 |
|  | 0.125 | 95.2 | 13.8 | 14.55 | -4.8 |  |
|  | 1.25 | 94.9 | 5.6 | 5.90 | -5.1 |  |
|  | 5 | 112.7 | 11.9 | 10.51 | 12.7 |  |
| TRIMIPRAMINE | 0.025 | 93.5 | 12.5 | 13.34 | -6.6 | 0.125 |
|  | 0.125 | 104.4 | 8.3 | 7.91 | 4.4 |  |
|  | 1.25 | 95.2 | 17.4 | 18.27 | -4.8 |  |
|  | 5 | 111.2 | 9.6 | 8.63 | 11.2 |  |
| U-47700 | 0.025 | 99.4 | 13.4 | 13.53 | -0.6 | 0.125 |
|  | 0.125 | 104.2 | 4.1 | 3.93 | 4.2 |  |
|  | 1.25 | 101.6 | 6.0 | 5.94 | 1.6 |  |
|  | 5 | 104.5 | 11.2 | 10.74 | 4.5 |  |
| UR-144 | 0.025 | 103.0 | 17.9 | 17.37 | 3.0 | 0.025 |
|  | 0.125 | 108.6 | 7.6 | 7.02 | 8.6 |  |
|  | 1.25 | 115.2 | 14.1 | 12.21 | 15.2 |  |
| UR-144 metabolite | 0.025 | 96.7 | 17.5 | 18.07 | -3.3 | 0.025 |
|  | 0.125 | 102.4 | 9.0 | 8.81 | 2.4 |  |
|  | 1.25 | 108.7 | 9.7 | 8.96 | 8.7 |  |
| W-15 | 0.025 | 105.5 | 4.4 | 4.15 | 5.5 | 0.125 |
|  | 0.125 | 99.7 | 6.2 | 6.22 | -0.3 |  |
|  | 1.25 | 100.7 | 6.0 | 5.95 | 0.7 |  |
|  | 5 | 114.2 | 9.3 | 8.15 | 14.2 |  |
| WIN 54.461 | 0.025 | 104.4 | 8.1 | 7.79 | 4.4 | 0.025 |
|  | 0.125 | 105.5 | 11.5 | 10.93 | 5.5 |  |
|  | 1.25 | 101.5 | 8.1 | 8.00 | 1.5 |  |
| WIN 55.212-2 | 0.025 | 94.3 | 10.2 | 10.86 | -5.8 | 0.025 |
|  | 0.125 | 89.0 | 6.9 | 7.73 | -11.0 |  |
|  | 1.25 | 93.8 | 13.0 | 13.86 | -6.2 |  |
| XLR-11 | 0.025 | 98.6 | 12.7 | 12.92 | -1.4 | 0.025 |
|  | 0.125 | 101.1 | 9.6 | 9.54 | 1.1 |  |
|  | 1.25 | 108.8 | 10.3 | 9.47 | 8.8 |  |
| XLR12 | 0.025 | 101.3 | 5.5 | 5.39 | 1.3 | 0.025 |
|  | 0.125 | 103.5 | 4.9 | 4.74 | 3.5 |  |
|  | 1.25 | 109.0 | 10.9 | 9.97 | 9.0 |  |
| YANGONIN | 0.125 | 90.3 | 17.2 | 19.08 | -9.7 | 0.125 |
|  | 1.25 | 119.6 | 14.3 | 11.93 | 19.6 |  |
|  | 5 | 114.5 | 2.9 | 2.53 | 14.5 |  |
| ZALEPLON | 0.025 | 100.1 | 10.5 | 10.52 | 0.1 | 0.125 |
|  | 0.125 | 99.3 | 9.7 | 9.80 | -0.7 |  |
|  | 1.25 | 102.9 | 11.9 | 11.53 | 2.9 |  |
|  | 5 | 119.4 | 6.1 | 5.07 | 19.4 |  |
| ZOLPIDEM | 0.025 | 89.9 | 6.9 | 7.66 | -10.1 | 0.125 |
|  | 0.125 | 100.5 | 7.9 | 7.90 | 0.5 |  |
|  | 1.25 | 97.1 | 6.8 | 7.04 | -2.9 |  |
|  | 5 | 95.5 | 3.0 | 3.15 | -4.5 |  |
| ZOPICLONE | 0.025 | 34.8 | 14.6 | 42.00 | -65.3 | 0.125 |
|  | 0.125 | 99.3 | 9.1 | 9.13 | -0.7 |  |
|  | 1.25 | 112.0 | 17.1 | 15.28 | 12.0 |  |
|  | 5 | 112.3 | 15.3 | 13.66 | 12.3 |  |
| ZUCLOPENTHIXOL | 0.025 | 139.8 | 36.4 | 26.01 | 39.8 | 0.125 |
|  | 0.125 | 108.6 | 17.9 | 16.46 | 8.6 |  |
|  | 1.25 | 117.1 | 12.6 | 10.75 | 17.1 |  |
|  | 5 | 153.4 | 27.5 | 17.91 | 53.4 |  |
| α-HYDROXYMIDAZOLAM | 0.025 | 131.4 | 26.6 | 20.22 | 31.4 | 0.125 |
|  | 0.125 | 86.3 | 12.6 | 14.63 | -13.7 |  |
|  | 1.25 | 89.5 | 4.9 | 5.43 | -10.5 |  |
|  | 5 | 108.3 | 6.5 | 5.98 | 8.3 |  |
